# Distinct phosphorylation states of mammalian CaMKIIβ control the induction and maintenance of sleep

**DOI:** 10.1371/journal.pbio.3001813

**Published:** 2022-10-04

**Authors:** Daisuke Tone, Koji L. Ode, Qianhui Zhang, Hiroshi Fujishima, Rikuhiro G. Yamada, Yoshiki Nagashima, Katsuhiko Matsumoto, Zhiqing Wen, Shota Y. Yoshida, Tomoki T. Mitani, Yuki Arisato, Rei-ichiro Ohno, Maki Ukai-Tadenuma, Junko Yoshida Garçon, Mari Kaneko, Shoi Shi, Hideki Ukai, Kazunari Miyamichi, Takashi Okada, Kenta Sumiyama, Hiroshi Kiyonari, Hiroki R. Ueda

**Affiliations:** 1 Laboratory for Synthetic Biology, RIKEN Center for Biosystems Dynamics Research, Suita, Osaka, Japan; 2 Department of Systems Pharmacology, Graduate School of Medicine, The University of Tokyo, Bunkyo-ku, Tokyo, Japan; 3 Thermo Fisher Scientific K.K., Yokohama, Kanagawa, Japan; 4 Graduate school of Medicine, Osaka University, Suita, Osaka, Japan; 5 Faculty of Medicine, The University of Tokyo, Bunkyo-ku, Tokyo, Japan; 6 Laboratory for Animal Resources and Genetic Engineering, RIKEN Center for Biosystems Dynamics Research, Chuo-ku, Kobe, Hyogo, Japan; 7 Laboratory for Comparative Connections, RIKEN Center for Biosystems Dynamics Research, Chuo-ku, Kobe, Hyogo, Japan; 8 Division of Molecular and Medical Genetics, Center for Gene and Cell Therapy, The Institute of Medical Science, the University of Tokyo, Minato-city, Tokyo, Japan; 9 Laboratory for Mouse Genetic Engineering, RIKEN Center for Biosystems Dynamics Research, Suita, Osaka, Japan; Charité - Universitätsmedizin Berlin, GERMANY

## Abstract

The reduced sleep duration previously observed in *Camk2b* knockout mice revealed a role for Ca^2+^/calmodulin-dependent protein kinase II (CaMKII)β as a sleep-promoting kinase. However, the underlying mechanism by which CaMKIIβ supports sleep regulation is largely unknown. Here, we demonstrate that activation or inhibition of CaMKIIβ can increase or decrease sleep duration in mice by almost 2-fold, supporting the role of CaMKIIβ as a core sleep regulator in mammals. Importantly, we show that this sleep regulation depends on the kinase activity of CaMKIIβ. A CaMKIIβ mutant mimicking the constitutive-active (auto)phosphorylation state promotes the transition from awake state to sleep state, while mutants mimicking subsequent multisite (auto)phosphorylation states suppress the transition from sleep state to awake state. These results suggest that the phosphorylation states of CaMKIIβ differently control sleep induction and maintenance processes, leading us to propose a “phosphorylation hypothesis of sleep” for the molecular control of sleep in mammals.

## Introduction

The sleep–wake cycle, like the circadian clocks, is a physiological function that governs the organism-level behavioral rhythms and is believed to regulate synaptic function [1]. However, the molecular mechanisms regulating the daily amount of sleep and the transitions between sleep and wake phases are not fully understood. Genetic screening studies have revealed that protein kinases play an important role in sleep duration regulation. In particular, knocking out the first sleep-promoting kinases discovered, *Camk2a* and *Camk2b*, markedly reduced sleep duration in mice [2]. Subsequent phosphoproteomics studies have shown that the phosphorylation states of neuronal proteins vary with the sleep–wake cycle and in response to sleep deprivation [3–5]. The phosphoproteomics profile revealed an alteration of the phosphorylation states of Ca^2+^/calmodulin-dependent protein kinase II (CaMKII)α/CAMKIIβ and its potential substrates (e.g., Synapsin 1). These results suggest that CaMKIIα/CaMKIIβ plays an important role in mammalian sleep regulation and support the phosphorylation hypothesis of sleep (the idea that sleep is regulated by protein phosphorylation). Because knockout mice of *Camk2b* showed the most pronounced decrease in sleep duration per day compared with *Camk2a* knockout mice [2], this study will focus on CaMKIIβ.

The phosphorylation hypothesis of sleep [2,6] assumes that the neural activity associated with wakefulness acts as an *input* to activate sleep-promoting kinases such as CaMKIIα/CaMKIIβ [2], SIK1/SIK2/SIK3 [7,8], and ERK1/ERK2 [9]. Another prediction is that sleep-promoting kinases may need to *store* some form of information associated with wakefulness. This is because awakening does not immediately lead to sleep but rather *stores* a history of awakening as a sleep need. CaMKIIβ as well as other CaMKII isoforms has unique features that might make this kinase suitable for achieving the *storage* of information associated with wakefulness. A well-known mechanism of CaMKIIβ activation is the intracellular Ca^2+^ influx that occurs upon excitatory synaptic input and subsequent neuronal firing [10,11]. Intracellular Ca^2+^ binds to calmodulin (CaM), which binds to CaMKIIβ and switches its kinase domain to the exposed open and kinase-active form. The kinase-active CaMKIIβ undergoes autophosphorylation along with phosphorylation of other substrate proteins. T287 are the first residues to undergo autophosphorylation upon activation of CaMKIIβ. T287 phosphorylation switches CaMKIIα/CaMKIIβ to its kinase-active form even in the absence of Ca^2+^/CaM [12–14]. The maintained kinase activity due to T287 phosphorylation is called autonomous activity. The regulation process of CaMKII by autophosphorylation serves as a neuronal timer in a minutes timescale in fruits fly [15]. However, the effect and mechanism of CaMKIIβ on sleep regulation and duration in mammals have not been rigorously investigated.

Furthermore, the dynamics of the sleep–wake cycle are characterized not only by the duration of sleep, but also by the distribution of sleep and wake episodes. Indeed, *Camk2b* knockout mice are less likely to transition from wake to sleep and from sleep to wake [2]. This suggests that CaMKIIβ elicits the transition between wake and sleep. It should be noted that sleep duration and sleep–wake transition can be independently regulated: For example, knocking out *orexin* barely affects sleep duration but significantly increases the sleep–wake transition [16,17]. Given the physiological process of the sleep–wake cycle, it is reasonable to assume that organisms employ multiple and stepwise mechanisms to regulate sleep. It would begin with sleep induction and switch to sleep maintenance. CaMKIIβ itself undergoes multiple and stepwise changes (multisite autophosphorylation and conformational changes) [10,18]. Following the phosphorylation of T287, the activated kinase catalyzes the autophosphorylation of residues such as T306 and T307 (CaMKIIβ). Phosphorylation of these residues inhibits the binding of Ca^2+^/CaM to CaMKIIβ [19–21]. The autoregulatory mechanism of CaMKIIβ may be more complex than a 2-step regulation. It was reported that autophosphorylation can occur in multiple residues other than well-understood T287/T306/T307 with different efficiency depending on residues [22]. The dodecameric CaMKIIβ structure may have many intermediate states [23]. Although the sleep–wake cycle affects the level of such multisite autophosphorylation of CaMKIIα and CaMKIIβ [3–5,24,25], little is known about the actual function of the multisite autophosphorylation in the regulation of the sleep–wake cycle.

## Results

### Phosphorylation of CaMKIIβ regulates sleep induction

**Fig 1:**
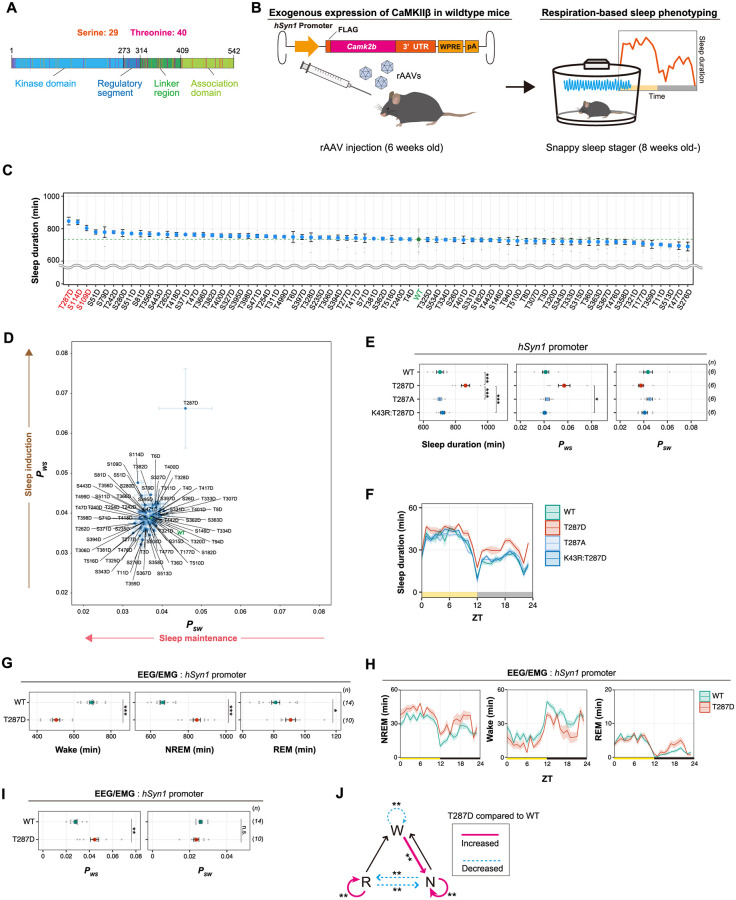
Phosphorylation of CaMKIIβ regulates sleep induction. **(A)** The 29 serine and 40 threonine residues throughout CaMKIIβ. Orange and magenta lines represent serine and threonine residues, respectively. Color-coded regions indicate the functional domains of CaMKIIβ. **(B)** Schematic diagram of AAV-based CaMKIIβ expression and respiration-based sleep phenotyping. **(C)** Daily sleep duration of mice expressing CaMKIIβ phosphomimetic mutants (*n* = 6–10) in the presence of endogenous WT CaMKIIβ. The represented value is the average of SSS measurements over 6 days. The dashed green line represents the average sleep duration of WT CaMKIIβ-expressing mice (WT, *n* = 48). Residues shown in red were selected for further analysis in an independent set of experiments. **(D)** Correlation diagram of daily *P*_*WS*_ and *P*_*SW*_ of mice expressing the CaMKIIβ phopshomimetic mutants shown in **Fig 1C**, averaged over 6 days. **(E, F)** Sleep/wake parameters **(E)** and sleep profiles **(F)** of mice expressing T287-related CaMKIIβ mutants, averaged over 6 days. Measurements are independent of those in **(C, D)**. The shaded area represents the SEM. Multiple comparison tests were performed between all individual groups. **(G-I)** Sleep parameters **(G and I)** and sleep profiles **(H)** measured by EEG/EMG recordings for mice expressing CaMKIIβ WT or the T287D mutant under the *hSyn1* promoter. **(J)** Differences in transition probabilities (between wakefulness (W), NREM sleep (N), and REM sleep (R)) between WT CaMKIIβ or T287D-expressing mice under the *hSyn1* promoter. Magenta lines and dashed blue lines indicate when the values for the T287D-expressing mice are significantly (*p* < 0.05) higher and lower, respectively. The underlying data can be found in S1 Data. Error bars: SEM, *p < 0.05, **p < 0.01, ***p < 0.001, n.s., no significance. See also S1–S5 Figs and S1 Table. AAV, adeno-associated virus; CaMKIIβ, calmodulin-dependent protein kinase IIβ; EEG, electroencephalogram; EMG, electromyogram; *hSyn1*, human *synapsin-1*; NREM, nonrapid eye movement; pA, polyA; REM, rapid eye movement; SSS, snappy sleep stager; UTR, untranslated region; WT, wild-type; ZT, zeitgeber time.

To investigate whether CaMKIIβ regulates sleep depending on the phosphorylation state of CaMKIIβ, we conducted an in vivo comprehensive phosphomimetic screening of CaMKIIβ. Mouse CaMKIIβ protein has 69 serine (S) and threonine (T) residues that can be the target of autophosphorylation (Fig 1A). Because it is still challenging to comprehensively predict phosphorylation residues based on sequence and structural information, we decided to assess the contribution of these 69 residues to sleep regulation by expressing a series of phosphomimetic mutants of CaMKIIβ, in which aspartic acid (D) replaced one of the phosphorylable 69 residues. Note that some of the mutated residues would not be the target of phosphorylation in vivo, the outcome of this screening does not directly confer the evidence of phosphorylation-dependent control.

Each of the 69 CaMKIIβ mutants was expressed under the control of human *synapsin-1* (*hSyn1*) promoter and delivered in wild-type (WT) mice brain by an adeno-associated virus (AAV) system AAV-PHP.eB [26], which allows broad gene expression throughout the brain. The whole-brain expression of H2B-mCherry reporter under the *hSyn1* promoter delivered by the AAV system was confirmed by whole-brain imaging using the CUBIC method (S2 Fig). The amount of AAV-induced CaMKIIβ expression is lower than the level of endogenous CaMKIIβ and does not affect the overall expression level of endogenous CaMKIIβ (S3A Fig). Unless otherwise indicated, we refer to mice with AAV-mediated expression of CaMKIIβ mutants simply by the mutant name (e.g., T287D mice). We measured the sleep parameters of the mice expressing mutant CaMKIIβ using a respiration-based sleep phenotyping system, snappy sleep stager (SSS) [16] (Fig 1B). Sleep measurements were started at 8 weeks old following the AAV administration at 6 weeks old. Mice expressing AAV-induced WT CaMKIIβ and untreated mice had similar daily sleep durations (733.9 ± 6.1 and 724.7 ± 4.3 min [16], respectively; all mice phenotypes are reported as mean ± SEM). In this screening, mice expressing T287D, S114D, or S109D CaMKIIβ mutants had the top 3 extended daily sleep duration (846.7 ± 23.7, 839.7 ± 14.1, or 803.4 ± 16.2 min, respectively) (Fig 1C). There was no correlation between the ensemble of sleep duration and AAV transduction efficiency among the analyzed mutants (S3B Fig), indicating that the observed sleep phenotypes can be attributed to the nature of the introduced mutations rather than to a possible difference in AAV transduction efficacy. The sleep parameter analyzed by SSS also includes transition probability from wake episode to sleep episode (*P*_*WS*_) and that from sleep episode to wake episode (*P*_*SW*_). The extended sleep duration can be due to either increased *P*_*WS*_ or decreased *P*_*SW*_. Increased *P*_*WS*_ can be interpreted as increased sleep *induction* efficiency (higher probability of switching from awake phase to sleep phase), while decreased *P*_*SW*_ can be interpreted as increased sleep *maintenance* capability (lower probability of switching from sleep phase to awake phase). The *P*_*WS*_—*P*_*SW*_ plot suggest that the top sleep extended T287D mutant had high sleep induction activity (i.e., high *P*_*WS*_) compared with WT (Fig 1D).

To confirm the reproducibility of the extended sleep duration for T287D, S114D, and S109D mice, we conducted an independent set of experiments. These confirmed the prolonged sleep duration of T287D mice (861.9 ± 26.1 min) and the increase in *P*_*WS*_ (Fig 1E). The extended sleep duration of T287D mice does not depend on the circadian timing because the mice showed increased sleep duration at most zeitgeber time of the day (Fig 1F). Consistent with this, we can observe increased *P*_*WS*_ of T287D mice both in light and dark phases, although clear statistical significance could not be obtained due to the relatively large variability of *P*_*WS*_ (S3C and S3D Fig). A closer investigation of the magnitude of increase in the amount of sleep suggested that sleep duration increases more significantly during the dark phase (Fig 1F and S3D Fig; see also S4A Fig). We speculate that this may be because there is less range for further sleep induction during the light phase, which is the native sleep phase, and the external light environment itself should also promote sleep. On the other hand, changes in the *P*_*WS*_ in T287D mice is more evident in the light phase, presumably because wake-associated signals of environmental dark environment antagonize the sleep-inducing effect of T287D mutant. The second round of evaluation did not show a significant increase in the sleep duration of S114D mice and S109D (S3E and S3F Fig). Thus, we concluded that T287D CaMKIIβ is the mutant that robustly increased sleep duration in vivo.

Replacing T287 with the nonphosphorylatable alanine (A) did not extend sleep duration (701.5 ± 9.8 min) (Fig 1E and 1F). This supports that the phosphorylation-mimicking property of D caused the sleep duration extension. Furthermore, the extended sleep duration depends on the kinase activity of CaMKIIβ, because the kinase-dead (K43R) version of the T287D mutant (i.e., K43R:T287D) did not extend sleep duration (719.8 ± 12.4 min) (Fig 1F). Given that the phosphorylation of T287 inhibits the interaction between the kinase domain and the regulatory segment of CaMKIIβ (which leads to the open and kinase-active conformation of the kinase), the normal sleep duration of K43R:T287D mice suggests that CaMKIIβ with open conformation alone is insufficient to lengthen sleep duration. We thus propose that T287-phosphorylated CaMKIIβ induces sleep via its kinase activity.

### Phosphorylation of CaMKIIβ regulates NREM sleep induction and sleep needs

To examine the detailed changes in the sleep architecture of T287D mice, we recorded electroencephalograms (EEGs) and electromyograms (EMGs). The EEG/EMG recordings revealed that T287D mice had significantly higher daily nonrapid eye movement (NREM) and REM sleep duration (Fig 1G and 1H) and *P*_*WS*_ (Fig 1I) than WT-expressing mice. The increased NREM and REM sleep duration as well as increased *P*_*WS*_ were observed in both light and dark phases (S4A Fig). The analysis of transition probabilities between wake, NREM, and REM episodes revealed a large decrease (*p* < 0.001) in wake maintenance (W to W) and increase (*p* < 0.001) in the transitions from wake to NREM (W to N) compared with WT-expressing mice (Fig 1J). These results suggest that the T287 phosphorylation of CaMKIIβ induces sleep by increasing wake to NREM transitions. Besides, we found that T287D mice have a slightly elevated trend in delta power (*p* = 0.15) and slow power (*p* = 0.07) during sleep episodes (S3B and S3C Fig). The level of EEG delta power during the NREM sleep episode has been shown to correlate with sleep need [27]. Franken and colleagues developed a mathematical model-based analysis to estimate the dynamics of sleep need [27]. We applied the same method to our EEG data. The simulation results suggest that T287D mice exhibit a significantly higher time constant for the decrease of sleep need (i.e., NREM delta power) during NREM sleep (S4D Fig). This is consistent with the idea that constitutive active T287D produces sleep need even during the NREM sleep phase, so that longer time is required for the reduction of sleep need during NREM sleep.

In a complementary approach, we demonstrated that the arousal system in T287D mice is normal by evaluating their responses to external stimuli. The novel cage environment promotes awakening by stimulating the mice’s exploratory behavior [28]. Cage exchange significantly decreased the sleep duration of T287D, WT, and PBS-administrated mice compared with the baseline duration (S4E and S4F Fig), suggesting that the sleep-extending effect of the T287D mutant is not due to abnormalities in the arousal system.

The robust sleep induction by the T287D mutant suggests that T287 phosphorylation marks the level of sleep need. This has been supported through previous studies. For example, western blotting analyses showed that the level of CaMKIIα T286/CaMKIIβ T287 phosphorylation is elevated after 6 h of sleep deprivation and then decreased during subsequent recovery sleep [4]. Moreover, the level of CaMKIIα T286 phosphorylation follows the expected sleep need along with normal sleep wake cycle: A previous study showed the circadian rhythmicity of CaMKIIα T286 phosphorylation peaking at the end of the dark (wake) phase and decreasing throughout the light (sleep) phase [5]. Consistent with this rhythmicity, another study indicated CaMKIIα T286 phosphorylation is higher at the dark (wake) phase [3]. Because several studies focus on CaMKIIα and rely on western blotting technique, we also examined whether the phosphorylation levels of CaMKIIβ T287 in the brain increased upon 6 h sleep deprivation by using a quantitative and targeted selected reaction monitoring (SRM) analysis [29]. The SRM analysis confirmed that sleep deprivation increased T287 phosphorylation of endogenous CaMKIIβ without changing the amount of total CaMKIIβ (S5A and S5B Fig). In addition, the phosphorylation level of CaMKIIα T286 and CaMKIIβ T287 correlated well, suggesting that these phosphorylation levels similarly respond to sleep deprivation (S5C and S5D Fig).

### Kinase activity of CaMKII regulates sleep

**Fig 2:**
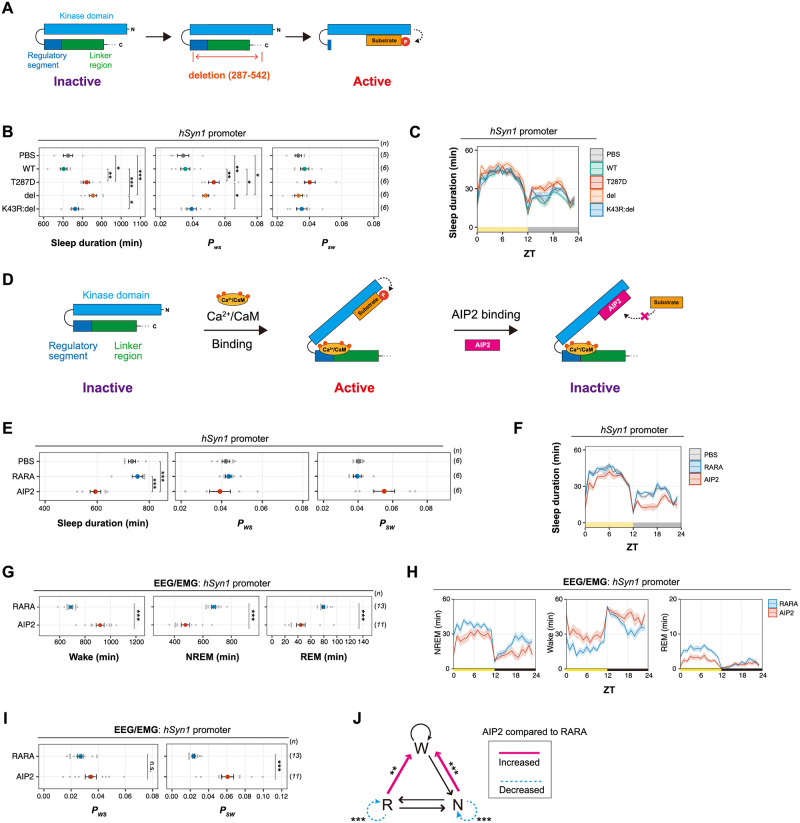
Perturbation on the kinase activity of CaMKII affects sleep duration. **(A)** Schematic diagram of CaMKIIβ activation via deletion of its C-terminus. Deletion of the regulatory segment and linker region exposes the kinase domain of the CaMKIIβ and makes the enzyme constitutively activated. **(B, C)** Sleep/wake parameters **(B)** and sleep profiles **(C)** of mice expressing the CaMKIIβ del mutant, averaged over 6 days. The shaded areas represent SEM. Multiple comparison tests were performed between all individual groups. **(D)** Schematic diagram of CaMKII inhibition by AIP2 expression. AIP2 competitively binds to the kinase domain and inhibits substrate phosphorylation. **(E, F)** Sleep/wake parameters **(E)** and sleep profiles **(F)** of mice expressing AIP2 or the RARA mutant measured by the SSS, averaged over 6 days. PBS: PBS-injected mice (*n* = 6). **(G-I)** Sleep phenotypes **(G and I)** and sleep profiles **(H)** of mice expressing AIP2 or the RARA mutant measured by EEG/EMG recordings. **(J)** Differences in transition probabilities (between wakefulness (W), NREM sleep (N), and REM sleep (R)) between mice expressing AIP2 or the RARA mutant. Magenta lines and dashed blue lines indicate when the values for the AIP2-expressing mice are significantly (*p* < 0.05) higher and lower, respectively. The underlying data can be found in S1 Data. Error bars: SEM, *p < 0.05, **p < 0.01, ***p < 0.001, n.s.: no significance. See also S6–S8 Figs. AIP2, autocamtide inhibitory peptide 2; CaM, calmodulin; CaMKII, calmodulin-dependent protein kinase II; del, deletion; EEG, electroencephalogram; EMG, electromyogram; *hSyn1*, human *synapsin-1*; NREM, nonrapid eye movement; REM, rapid eye movement; SSS, snappy sleep stager; WT, wild-type; ZT, zeitgeber time.

The 287D is well characterized as a constitutive kinase–active CaMKIIβ mutant. To confirm the importance of CaMKIIβ kinase activity in the sleep induction, we next tested another type of constitutive-active CaMKIIβ mutant. We used CaMKIIβ deletion (del) mutant that lacks the C-terminal half involving the regulatory segment, linker region, and oligomerization domain [30]. The CaMKIIβ del mutant is constitutively active due to the exposed kinase domain but does not retain T287 and subsequent residues (Fig 2A). Similar to T287D mice, mice expressing the del mutant showed an extended sleep duration and increased *P*_*WS*_ (Fig 2B and 2C). As in the case of T287D, a tendency for increased sleep duration and *P*_*WS*_ in the del mutant was observed in both light and dark phases, and the relative amount of increased sleep duration compared with WT is higher in the dark phase (Fig 2C, S6A and S6B Fig). The extended sleep duration depends on the kinase activity because mice expressing the del mutant with the K43R point mutation (K43R:del) and the WT-expressing mice had similar sleep phenotypes. These results support that the constitutive kinase activity of CaMKIIβ induces sleep.

We carried out a complementary approach by inhibiting the kinase activity of endogenous CaMKII. We used autocamtide inhibitory peptide 2 (AIP2), which inhibits the enzyme activity of CaMKIIα and CaMKIIβ by binding to the kinase domain (Fig 2D) [31,32]. Mice expressing the mCherry-fused AIP2 exhibited a decreased sleep duration and *P*_*WS*_ along with a trend toward increased *P*_*SW*_ compared with mice expressing the inactive mutant of AIP2 (RARA) (Fig 2E and 2F), demonstrating that the CaMKIIα/CaMKIIβ kinase activity is critical for normal sleep induction and maintenance. The expression of the AIP2 RARA mutant appears to have no effect on the sleep duration compared with PBS-administrated mice. These results were consistent with the phenotype of *Camk2a* or *Camk2b* knockout mice [2], except for the *P*_*SW*_ change: The genetic knockout of *Camk2a* or *Camk2b* slightly decreased *P*_*SW*_. This difference might account for the postnatal and kinase activity-targeted inhibition of CaMKIIα/CaMKIIβ by AIP2 expression. Similar to the case of T287D mutant, reduced sleep duration by AIP2 expression was observed both in light and dark phases, but the magnitude of effect in the amount of sleep is higher in the dark phase (Fig 2F, S6C and S6D Fig). This may be because the sleep-promoting effect of environmental light antagonizes the sleep inhibition effect of AIP2 expression. Unexpectedly, we also noticed that the increased *P*_*SW*_ by AIP expression is mostly observed in the dark phase (S6C and S6D Fig). We do not currently have a straightforward explanation for this observation, but some states of CaMKIIα/CaMKIIβ related to the interaction with AIP2 might be modulated by external light conditions. Further investigation of AIP2-expressed mice phenotypes, for example, observation in constant light or dark conditions, will be required for clarifying this point.

The EEG/EMG recording of mice expressing AIP2 showed a significant decrease in NREM and REM sleep duration (Fig 2G–2I). The increased transition probability from NREM/REM to wake and decreased transition to keep NREM and REM episodes in AIP2-expressing mice suggested that CaMKIIα/CaMKIIβ inhibition impaired the maintenance mechanism of NREM/REM sleep (Fig 2J). Relatively higher *P*_*SW*_ during the dark phase than during the light phase was also observed in the EEG/EMG recording (S7A Fig). There was no significant change in normalized delta power during NREM sleep (S7B and S7C Fig). Note that there were differences in the waveforms of the EEG represented by the increased power of slow-wave oscillations (0.5 Hz to 1 Hz) in all 3 states of vigilance (S7D Fig), although no difference was observed in the local field potential recordings of awaking mice cortex with the adult deletion of both *Camk2a* and *Camk2b* [33]. Consistent with the phenotype of AIP2-expressed mice, EEG/EMG analysis showed that *Camk2b* knockout mice had decreased NREM and REM duration (S8A–S8D Fig) as well as decreased *P*_*SW*_. *Camk2b* knockout mice might have a decreased delta power, although we could not conclude on this because the changes in delta power depend on the normalization procedure of the EEG power spectrum (S8E–S8H Fig). The reduced sleep duration in SSS by AIP2-expression or *Camk2b* knockout can be attributed to the reduced NREM sleep because NREM sleep constitutes the most portion of total sleep time, although CaMKIIα/CaMKIIβ may also have a role in the control of REM sleep as observed in reduced REM sleep duration in these EEG/EMG recordings.

### Phosphorylation of CaMKIIβ in excitatory neurons regulates sleep induction

**Fig 3:**
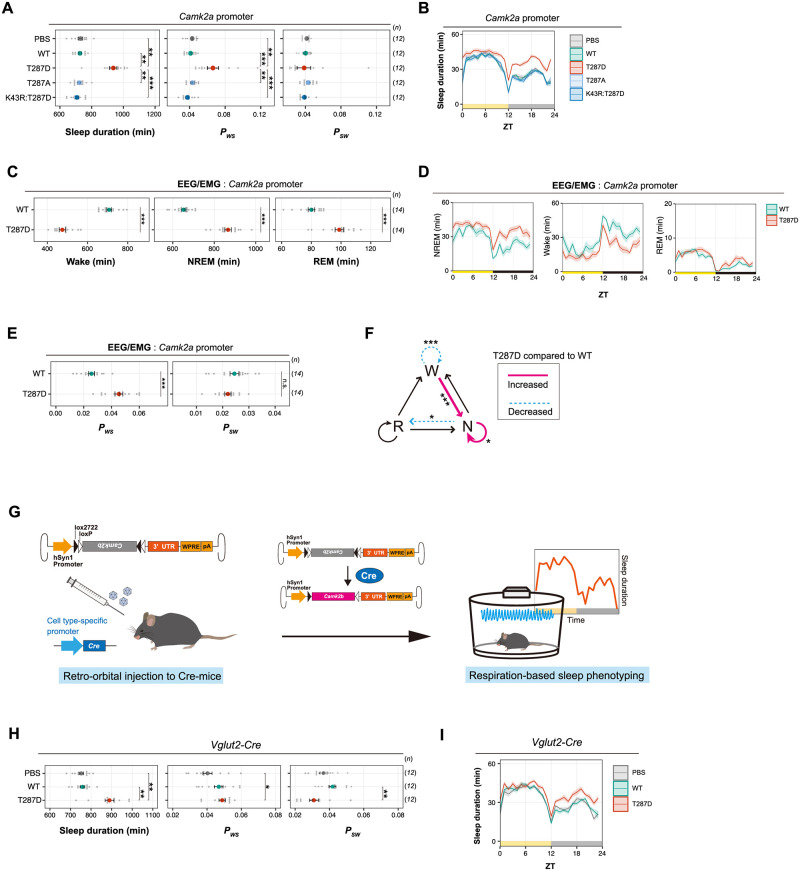
Phosphorylation of CaMKIIβ in excitatory neurons regulates sleep induction. **(A, B)** Sleep/wake parameters **(A)** and sleep profiles **(B)** of mice expressing CaMKIIβ T287-related mutants under the *Camk2a* promoter, averaged over 6 days. Shaded areas represent SEM. Multiple comparison tests were performed between all individual groups. **(C-E)** Sleep phenotypes **(C and E)** and sleep profiles **(D)** measured by EEG/EMG recordings for mice expressing CaMKIIβ (WT) or the T287D mutant under the *Camk2a* promoter. **(F)** Differences in transition probabilities (between wakefulness (W), NREM sleep (N), and REM sleep (R)) between mice expressing WT CaMKIIβ or the T287D mutant under the *Camk2a* promoter. Magenta lines and dashed blue lines indicate when the values for the T287D-expressing mice are significantly (*p* < 0.05) higher and lower, respectively. **(G)** Schematic diagram of cell type–specific expression of CaMKIIβ using AAV and Cre-mice. Cre-mediated recombination of AAV genomes results in *Camk2b* gene expression in the target cells. **(H, I)** Sleep/wake parameters **(H)** and sleep profiles **(I)** of *Vglut2-Cre*-mice administrated with AAV-DIO-*Camk2b*, averaged over 6 days. Shaded areas represent SEM. Multiple comparison tests were performed between all individual groups. The underlying data can be found in S1 Data. Error bars: SEM, *p < 0.05, **p < 0.01, ***p < 0.001, n.s.: no significance. See also S9 Fig. AAV, adeno-associated virus; CaMKIIβ, calmodulin-dependent protein kinase Iiβ; DIO, double-floxed inverted open reading frame; EEG, electroencephalogram; EMG, electromyogram; NREM, nonrapid eye movement; REM, rapid eye movement; WT, wild-type; ZT, zeitgeber time.

To investigate possible neuronal cell types responsible for the CaMKIIβ-mediated sleep induction, we expressed the CaMKIIβ mutants under the *Camk2a* promoter, which is a well-characterized promoter inducing gene expression preferentially to the excitatory neurons [34]. As Fig 3A and 3B show, the daily sleep duration of T287D mice was higher than that of WT-expressing mice, which is consistent with the results obtained with the *hSyn1* promoter. WT, T287A, K43R:T287D, and PBS-administrated mice had comparable sleep phenotypes (Fig 3A and 3B). As observed in T287D mice with the *hSyn1* promoter, T287D mice with the *Camk2a* promoter had a significantly higher *P*_*WS*_ (Fig 3A), suggesting that the T287-phosphorylated CaMKIIβ promotes the transition from wakefulness to sleep. These sleep phenotypes were also confirmed by EEG-EMG recording indicating the increased NREM/REM sleep duration (Fig 3C and 3D) and a higher *P*_*WS*_ (Fig 3E) with a higher transition rate from wake to NREM (Fig 3F). We also confirmed that T287D mice had significantly higher delta power during NREM sleep (S9A and S9B Fig) as suggested in *hSyn1*-driven expression of T287D mutant (S4B and S4C Fig), indicating elevated sleep needs. Similar to the case of *hSyn1*-driven expression, the model-based estimation of EEG delta power dynamics indicates that the expression of CaMKIIβ T287D mutant markedly suppresses the decrease of NREM delta power during NREM sleep (S9C Fig). We further reproduced the increased sleep duration by expressing the T287D mutant under the *Camk2b* promoter cloned in this study (S9D-S9F Fig).

The use of *Camk2a* promoter enriches the gene expression in excitatory neurons. However, the promoter also induces gene expression in other cell types [34]. To confirm the involvement of excitatory neurons in the CaMKIIβ-dependent sleep promotion, we then used another strategy by using AAVs carrying double-floxed inverted open reading frame (DIO) constructs and mouse lines expressing *Cre* recombinases in specific neurons (*Cre*-mice) (Fig 3G). CaMKIIβ T287D expression in *Vglut2-*specific neurons significantly increased sleep duration compared to the WT CaMKIIβ-expressing mice (Fig 3H and 3I). This result again suggests that glutamatergic excitatory neurons are involved in the sleep promotion by the CaMKIIβ T287D mutant.

**Fig 4:**
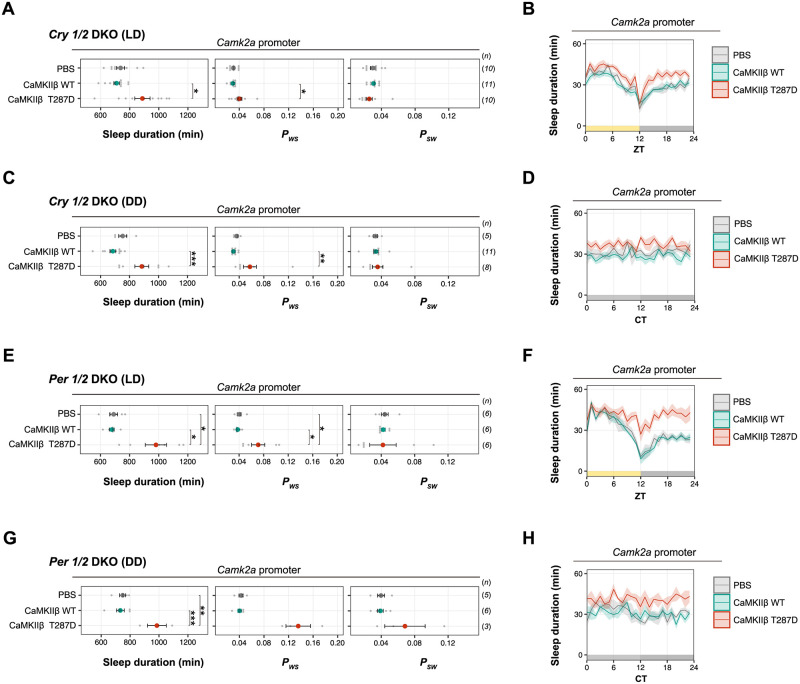
Sleep induction by CaMKIIβ T287D mutant is independent from core circadian clock genes. **(A-D)** Sleep/wake parameters and sleep profiles, averaged over 4 days, of *Cry1/2* DKO mice expressing WT CaMKIIβ or the T287D mutant under the LD condition **(A and B)** or DD **(C and D)**. Multiple comparison tests were performed between all individual groups. **(E-H)** Sleep/wake parameters and sleep profiles, averaged over 4 days, of *Per1/2* DKO mice expressing WT CaMKIIβ or the T287D mutant under the LD condition **(E and F)** or DD **(G and H)**. Multiple comparison tests were performed between all individual groups. The underlying data can be found in S1 Data. Error bars: SEM, *p < 0.05, **p < 0.01, ***p < 0.001, n.s.: no significance. CaMKIIβ, calmodulin-dependent protein kinase IIβ; CT, circadian time; DD, constant dark; DKO, double knockout; LD, light/dark; WT, wild-type; ZT, zeitgeber time.

Although we could not specify the downstream cellular or molecular targets responsible for sleep promotion, circadian core transcription repressors may not be involved in the sleep control by CaMKIIβ. We expressed CaMKIIβ T287D mutant in *Cry1*^−/−^:*Cry2*^−/−^ and *Per1*^−/−^:*Per2*^−/−^ double knockout mice (*Cry1/2* DKO and *Per1/2* DKO) using the *Camk2a* promoter. Both DKO mice lines are deficient in behavioral circadian rhythmicity in constant dark (DD) [35–38]. Under light/dark (LD) conditions, the daily sleep duration of T287D-expressing *Cry1/2* DKO and *Per1/2* DKO mice was significantly higher than that of WT CaMKIIβ-expressing mice (Fig 4A, 4B, 4E and 4F), and the relative magnitude of sleep increase is higher in the dark phase. Under DD, where both DKO mice lack a clear circadian behavioral rhythmicity, the sleep duration of T287D-expressing mice increased irrespective of circadian time across the 24 h (Fig 4C, 4D, 4G and 4H). This result also supports that the relatively higher effect on sleep duration during the dark phase observed in T287D mice is related to the interaction with environmental light conditions. The increased sleep duration under DD is associated with increased *P*_*WS*_. These results demonstrate that the sleep-inducing effect of the T287D mutant is independent of behavioral circadian rhythmicity and canonical core clock genes such as *Cry1/Cry2* or *Per1/Per2*.

### Multisite phosphorylation of CaMKIIβ regulates sleep stabilization

**Fig 5:**
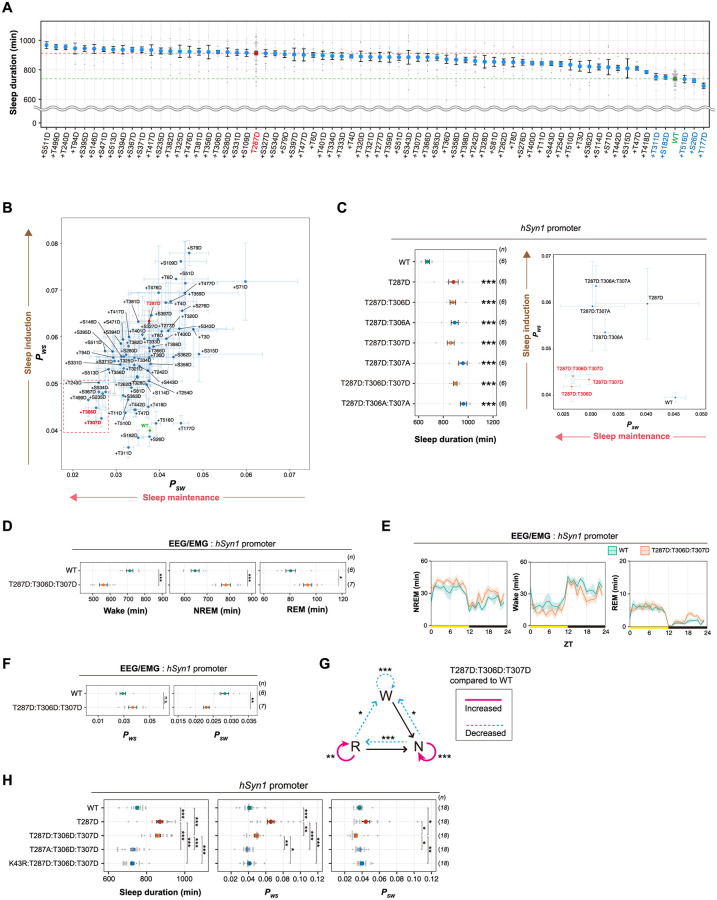
Multisite phosphorylation of CaMKIIβ regulates sleep maintenance. **(A)** Daily sleep duration, averaged over 6 days, of mice expressing CaMKIIβ double-phosphomimetic mutants (*n* = 5–12). The dashed green and red lines represent the averaged sleep duration of mice expressing WT CaMKIIβ (WT, *n* = 71) and T287D mutants (T287D, *n* = 68), respectively. The plus sign in a mutant’s name indicates a combination with T287D. Mutants marked in blue were selected for further analysis as sleep-canceling residues, related to Fig 6A. **(B)** Correlation diagram of daily *P*_*WS*_ and *P*_*SW*_ of mice expressing the CaMKIIβ double-phopshomimetic mutants shown in Fig 5A, averaged over 6 days. The mutants (including +T306, +T307 marked in red) in the dotted magenta box had extended sleep duration with lower *P*_*WS*_ and *P*_*SW*_ (i.e., higher sleep maintenance activity). **(C)** Sleep duration and correlation diagram of daily *P*_*WS*_ and *P*_*SW*_ of mice expressing CaMKIIβ mutants with D or A substitutions of T306 and/or T307 residues. For the comparisons of sleep duration, multiple testing was performed against WT CaMKIIβ-expressing mice. In the *P*_*WS*_-*P*_*SW*_ diagram, residues marked in red indicate mutants with low lower *P*_*WS*_ and *P*_*SW*_. **(D-F)** Sleep phenotypes of mice expressing WT CaMKIIβ or the T287D:T306D:T307D mutant measured by EEG/EMG recordings. **(G)** Differences in transition probabilities (between wakefulness (W), NREM sleep (N), and REM sleep (R)) between mice expressing WT CaMKIIβ or the T287D:T306D:T307D mutant. Magenta lines and dashed blue lines indicate when the values for the T287D:T306D:T307D-expressing mice are significantly (*p* < 0.05) higher and lower, respectively. **(H)** Sleep/wake parameters, averaged over 6 days, of mice expressing the CaMKIIβ T287D:T306D:T307D mutant with the K43R or T287A mutation. Multiple comparison tests were performed between all individual groups. The underlying data can be found in S1 Data. Error bars: SEM, *p < 0.05, **p < 0.01, ***p < 0.001, n.s.: no significance. See also S10 and S11 Figs. CaMKIIβ, calmodulin-dependent protein kinase IIβ; EEG, electroencephalogram; EMG, electromyogram; NREM, nonrapid eye movement; REM, rapid eye movement; WT, wild-type; ZT, zeitgeber time.

So far, we suggested CaMKIIβ phosphorylation at T287 residue exhibit sleep induction activity. A unique feedback control of CaMKIIβ activity is implemented by multisite autophosphorylation, where the autophosphorylation of T287 is followed by autophosphorylation at other residues such as T306 and T307. To survey the role of multisite phosphorylation in the sleep control, we created a series of double-phosphomimetic mutants of CaMKIIβ, in which besides T287, we mutated one of the remaining 68 S or T residues to D. Most of the analyzed these double-phosphomimetic mutants showed extended sleep duration similar to the T287D single mutant (Fig 5A and S10A Fig). However, such mutants with extended sleep duration can be subdivided into several types by using *P*_*WS*_ and *P*_*SW*_. Notably, several mutants such as T287D:T306D and T287D:T307D are located at the bottom-left corner of the *P*_*SW*_-*P*_*WS*_ plot, indicating that these mutants had lower *P*_*WS*_ and *P*_*SW*_ compared with single T287D mutants (Fig 5B). In other words, the extended sleep duration of these double mutants can lie in the increased sleep maintenance activity (i.e., decreased *P*_*SW*_) rather than sleep induction activity. Among the 7 double mutants with increased sleep maintenance activity, T287D:T306D, T287D:T307D, and T287D:S534D robustly exhibited prolonged sleep duration, unchanged *P*_*WS*_, and reduced *P*_*SW*_ compared with WT-expressing mice in the independent experiment (S10B and S10C Fig).

We focused on the sleep maintenance function of T306 and T307 because these residues are a well-known autonomous negative feedback control for CaMKIIβ kinase activation. We substituted these residues with the nonphosphorylatable residue alanine. The T287D:T306D:T307D and T287D:T306A:T307A mice both exhibited extended sleep duration compared to the WT (Fig 5C and S10D Fig). As with T287D single mutant, the prolonged sleep duration for the T287D:T306A:T307A can be explained by an increase in *P*_*WS*_ (i.e., sleep induction). On the other hand, the T287D:T306D:T307D mice showed decreased *P*_*WS*_ and *P*_*SW*_, indicating that the extended sleep duration can be explained by sleep maintenance rather than the sleep induction (Fig 5C). The proposed sleep maintenance effect is also supported by the increased sleep episode duration observed in T287D:T306D:T307D mice, while such effect is less evident in T287D mice (S10E Fig). A similar relationship can be observed between T287D:T306D and T287D:T307D mutants.

EEG/EMG recordings showed an increase in NREM and REM sleep duration (Fig 5D and 5E), a significant decrease in *P*_*SW*_, and no significant change in *P*_*WS*_ (Fig 5F), which is consistent with the SSS analysis. In both of SSS and EEG/EMG recordings, it is not clear whether there is a difference in the extent of the effect of T287D:T306D:T307D mutations on *P*_*SW*_ between light and dark phases (S10F and S11A Figs). The T287D:T306D:T307D mice had higher NREM to NREM and REM to REM transition probabilities than WT-expressing mice (Fig 5G). However, unlike T287D, this mutant did not increase the wake to NREM transition probability, suggesting that the additional phosphorylation(s) of T306 and/or T307 stabilize NREM and REM sleep. Mice expressing the T287D:T306D:T307D mutant and those expressing WT had similar delta power, but the mutant increased slow power (S11B and S11C Fig). Thus, additional phosphorylation of T306/T307 also seems to affect the quality of sleep.

To test whether the sleep maintenance function of the T287D:T306D:T307D mutant depends on its enzyme activity, we examined the sleep phenotype of mice expressing its kinase-dead version (K43R:T287D:T306D:T307D) and found that these mice did not exhibit a sleep-stabilizing phenotype (Fig 5H). Furthermore, the T287A:T306D:T307D mutant, in which T287 was replaced by a nonphosphorylatable A, also resulted in similar sleep parameters to WT. These results suggest that the sleep maintenance function of CaMKIIβ with phosphorylated T306 and T307 depends on its enzyme activity and that this function requires T287 phosphorylation. We thus propose that multisite phosphorylation of CaMKIIβ (residues T287, T306, and T307) converts the sleep-inducing effect of T287-phosphorylated CaMKIIβ into a sleep maintenance activity.

### Multisite phosphorylation of CaMKIIβ can cancel sleep induction

**Fig 6:**
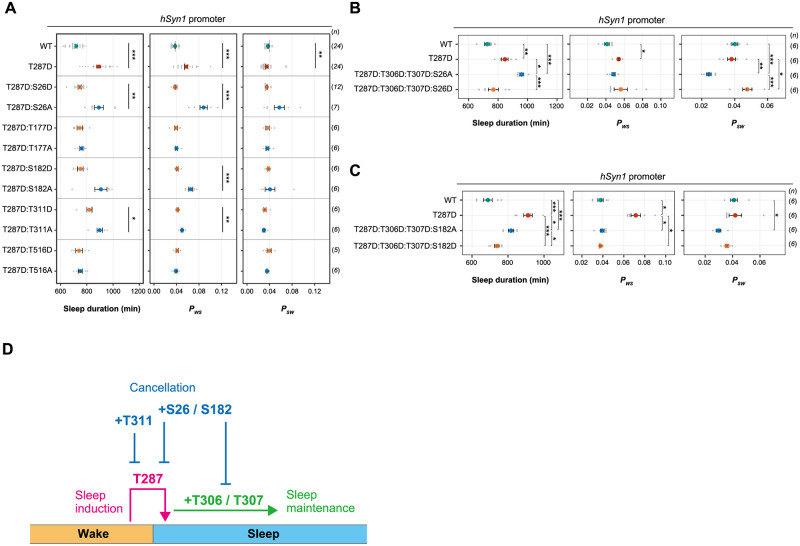
Multisite phosphorylation of CaMKIIβ can control sleep induction, maintenance, and cancellation of sleep induction/maintenance. **(A)** Sleep/wake parameters, averaged over 6 days, of mice expressing CaMKIIβ mutants with D or A substitutions of residues that cancel the sleep-inducing effect of T287D in Fig 5A. Measurements are independent from those in Fig 5A. **(B, C)** Sleep/wake parameters of mice expressing quadruple-phosphomimetic CaMKIIβ mutants related to S26 **(B)** and S182 **(C)**. Multiple comparison tests were performed between all individual groups. Error bars: SEM, *p < 0.05, **p < 0.01, ***p < 0.001. **(D)** Ordered multisite phosphorylation states of CaMKIIβ in sleep regulation. T287-phosphorylated CaMKIIβ induces sleep from wakefulness (sleep induction). Additional phosphorylation at T306 and T307 converts the sleep induction activity to the sleep maintenance activity. These sleep induction and maintenance activities are cancelled by the additional phosphorylation at S26, S182, and T311 (T311 seems to work only for cancellation of sleep induction, not for sleep maintenance). Note that this model only describes about the possible relationship between CaMKIIβ and the sleep–wake cycle without considering the difference between NREM and REM sleeps. The underlying data can be found in S1 Data. See also S12 Fig. CaMKIIβ, calmodulin-dependent protein kinase IIβ; NREM, nonrapid eye movement; REM, rapid eye movement.

The double-mutant screening also identified several mutants that exhibit a sleep phenotype similar to WT-expressing mice (Fig 5A). In other words, the additional D mutation cancels the sleep-inducing effect of T287D. We focused on the 5 mutants (T287D:S26D, T287D:T177D, T287D:S182D, T287D:T311D, and T287D:S516D) with the top 5 closest sleep parameters to WT, even if they had transduction efficiencies comparable to that of T287D (S10A Fig). To confirm that the observed phenotype of these 5 mutants came from the phosphomimetic property of D, we evaluated the phenotypes of nonphosphorylatable A mutants (Fig 6A). The T287D:S26A, T287D:S182A, and T287D:T311A mutants lost the effect of the D substitution, supporting the idea that phosphorylation of S26, S182, and T311 cancel the sleep-inducing effect of the coexisting T287 phosphorylation, in terms of sleep duration and/or *P*_*WS*_ alteration. On the other hand, the sleep phenotypes of T287D:T177A and T287D:T516A mice were similar to those of T287D:T177D, T287D:T516D, and WT mice. This indicates that both A and D substitutions for these residues disturb sleep inducing effect of coexisting T287D mutation and thus the effect of D mutant may not rely on its phosphomimetic property.

Among these sleep-canceling mutations, S26D and S182D can also antagonize the sleep maintenance effect of T287D:T306D:T307D mutant. Inclusion of S26A or S182A to the T287D:T306D:T307D recapitulated the sleep maintenance function observed in T287D:T306D:T307D mutant (Fig 6B and 6C), with decreased *P*_*SW*_ and a prolonged sleep episode duration. On the other hand, the substitution of S26 or S182 to D resulted in the loss of the sleep maintenance function. The mutant with the T311D substitution added to the T287D:T306D:T307D retained sleep maintenance activity (S12A Fig). In summary, the sleep induction and maintenance effect of CaMKIIβ elicited by T287 phosphorylation followed by T306 and T307 phosphorylation appears to be terminated by S26 and S182 phosphorylation, and phosphorylation of T311 may only cancel the sleep induction activity elicited by T287 phosphorylation.

Consistent with the kinase activity–dependent sleep promotion, phosphorylation of these sleep cancellation residues may inhibit the kinase activity of CaMKIIβ. We used cell lysates of 293T cells expressing the CaMKIIβ mutants, which can be used to measure the kinase activity of expressed CaMKII protein [39]. The cell lysates showed kinase activity towards conventional peptide substrate for CaMKII depending on both ectopic CaMKIIβ expression and addition of Ca^2+^/CaM, suggesting that the effects of endogenous CaMKII and other kinases are negligible. The expression levels of CaMKIIβ mutants were quantified and equalized for kinase assay (S12B Fig). The relative kinase activity measured by the level of phosphorylated substrate suggests that S26D, S182D, and T311D mutation leads to the loss of kinase activity similar to the kinase-dead K43R mutant (S12C Fig). The reason for the reduced kinase activity for each mutant is currently unknown. At least, S182D mutant showed no kinase activity because of the markedly reduced expression level.

### The sleep-controlling CaMKIIβ residues can be the target of autophosphorylation

Because CaMKII is known to switch its biochemical property through the autophosphorylation, it may be possible that some of the multisite phosphorylation status proposed above (i.e., multisite phosphorylations at S26, S182, T287, T306, T307, or T311) can be achieved through autophosphorylation. Indeed, T306 and T307 are well-established autophosphorylation target [19–21]. S26 (or S25 in CaMKIIα) and T311 are also suggested to be the target of autophosphorylation at least in cultured cell [22]. We also confirmed that those residues can be the target of autophosphorylation in a purified kinase system. We analyzed the time course changes in the phosphorylation levels of each sleep-controlling residues in CaMKIIβ (S26, S182, T287, T306, T307, and T311). The purified CaMKIIβ was incubated with CaM under various Ca^2+^ conditions within the reaction buffer. S12D Fig indicates that T287 phosphorylation occurs in the presence of 0.5 mM Ca^2+^ (conditions #3 and #4) but not in the absence of explicitly added Ca^2+^ in the reaction buffer (conditions #1 and #2). Addition of Ca^2+^ also promoted the S26 and S182 phosphorylations following to T287 phosphorylation. Note that it is hard to clearly separate the chromatogram of the peptide with S182 phosphorylation and that with the adjacent T177 phosphorylation (S12E Fig), so the quantification value of pS182 presented below include the signal from pT177 peptides.

T306 and T307 are effectively phosphorylated in the presence of low (condition #1, #2) or temporal (condition #4) Ca^2+^ in the reaction buffer. This is consistent with previous studies suggesting that the stable binding of Ca^2+^/CaM renders T306 and T307 inaccessible to the kinase domain of CaMKIIβ, and their phosphorylation requires the temporal removal of Ca^2+^/CaM from the kinase [40–43] or gradual phosphorylation of T306 and T307 occurs in the absence of apparent Ca^2+^ in the reaction buffer [44,45]. The low Ca^2+^ concentration condition also promotes T311 phosphorylation, which is spatially close to T306 and T307. The time course of T311 phosphorylation in condition #2 is different from that of T306 and T307 phosphorylation: The phosphorylation of T311 peaked 5 min after CaM addition and then decreased, presumably because of the progressive phosphorylation of T306 and T307.

In summary, the biochemical analysis suggests that S26, S182, T306, T307, T307, and T311 can be the target of autophosphorylation at least in our in vitro experimental condition. Time course of phosphorylation at each residue indicates that these autophosphorylation events proceed slower than T287 autophosphorylation (S12D Fig). Although we could not completely exclude the possibility that any contaminated kinase phosphorylated each residue especially for the unconventional autophosphorylation residues, it is unlikely that a misregulated or contaminated Ca^2+^/CaM-independent kinase activity phosphorylated S26, S182, and T311 residues because these phosphorylation are suppressed in the absence of Ca^2+^ (condition #4). We also confirmed that the phosphorylation of S26, T306, T307, T307, and T311 are depending on the kinase activity of CaMKIIβ in cultured cell. The phosphorylation levels of these residues are markedly increased in the constitutive kinase–active T287D mutant but not so in the WT or kinase-dead form of T287D mutant (i.e., K43R:T287D) expressed in the 293T cells (S12F Fig). This result also supports that these residues are the target of autophosphorylation at least in 293T cells, although we do not exclude the possibility that other kinases play a prominent role for the phosphorylation of these residues especially in the brain. We cannot quantify the S182 phosphorylation in the 293T condition because of our technical limitations in the detection of this peptide in crude samples.

## Discussion

In this study, we demonstrated that the conditional induction or inhibition of CaMKIIβ kinase activity could bidirectionally increase or decrease mammalian sleep duration. The bidirectional effect as well as the near 2-fold difference in sleep duration caused by the activation (e.g., 936.7 ± 22.6 min; Fig 3A) and inhibition (e.g., 593.1 ± 20.4 min; Fig 2E) of CaMKIIβ further supports the role of CaMKIIβ as a core sleep regulator, rather than auxiliary inputs that either induce or inhibit sleep upon environmental responses. Assuming the role of CaMKIIβ as one of the core kinases in the sleep control, the next question would be how CaMKIIβ relates to other phosphorylated enzymes, such as CaMKIIα [2], SIK1/SIK2/SIK3 [7,8], and ERK1/ERK2 [9], to shape the phosphorylation signaling network for sleep regulation.

The postnatal conditional expression of CaMKIIβ and its inhibitor changes the sleep phenotype, which rules out, at least in part, neuronal developmental abnormality potentially caused by the embryonic knockout of *Camk2b* [46]. Although the embryonic DKO of *Camk2a*/*Camk2b* caused developmental effects [33], the sleep reduction caused by the conditional expression of CaMKII inhibitor AIP2 supports that the reduction of kinase activity reduced sleep duration in the *Camk2a* KO and *Camk2b* KO mice [2], not the neuronal structural abnormality potentially caused by the gene knockout. Given the inducible adult deletion of both *Camk2a* and *Camk2b* resulted in lethal phenotype [33], our AIP2 expression condition would only partially inhibit the kinase activity of CaMKIIα and CaMKIIβ. *Camk2b* knockout mice have been also shown to have abnormal motor functions [47]. We do not completely exclude the possibility that such effects on motor functions especially during the awake phase indirectly affect the sleep phenotypes. However, it is unlikely that the observed sleep phenotypes are due to an artifact associated with such locomotor abnormalities because the EEG-EMG recording and respiration recording, both modalities independent of locomotor activity, confirmed a consistent sleep phenotype.

The effect of AIP2, kinase-inhibitory CaMKIIβ mutants (e.g., K43R and S26D) and the sleep-promoting effect of the truncated CaMKIIβ kinase domain indicate that the sleep-promoting effect of activated CaMKIIβ comes from the enzymatic activity of CaMKIIβ (Fig 2). Based on the observation that the expression level of CaMKIIβ with the AAV-based approach appeared to be low compared to the endogenous one. Thus, at least for the constitutive kinase–active and full-length CaMKIIβ mutants (e.g., T287D and T287D:T306D:T307D), it is reasonable to assume that the expressed CaMKIIβ mutants promote sleep by the formation of dodecamers with abundant endogenous CaMKIIα/β. On the other hand, the lower level of AAV-based CaMKIIβ expression may be not enough to induce the dominant negative effect expected for the kinase-dead form of CaMKIIβ (e.g., K43R:T287D) or CaM-inaccessible form of CaMKIIβ (e.g., single T306D mutant). It is possible that single T306D or T307D mutant can take a phosphorylation status similar to the T287D:T306D:T307D by taking phosphorylation at T287 residue from the neighboring kinase-active CaMKIIα/CaMKIIβ subunit. However, the result of the initial screening showed that neither the expression of single T306D nor T307D mutant exhibit sleep promotion activity. Thus, we speculate that the phosphorylation at T287 residue may not occur effectively in the single T306D or T307D mutant CaMKIIβ in our experimental conditions. It is also reasonable to assume that some part of single T287D mutant undergoes further autophosphorylation towards other residues, leading to the multiple phosphorylation states that cannot be recapitulated by the mutants tested in this study. Such complex phosphorylation states might be the reason for phenotype variation observed in T287D mice (e.g., *P*_*SW*_ variation between the initial screening (Fig 1D) and subsequent verification (Fig 1E)) Another phenotype variation observed in our initial screening (Fig 1D) and verification is extended sleep duration observed in S109D and S114D. The kinase assay in 293T cell lysate showed high kinase activity in the absence of CaM (S12C Fig), suggesting that S109D retains constitutive kinase activity similar to the T287D. However, the unstable and minor sleep promotion by S109D in vivo indicates that the kinase assay using a conventional peptide substrate does not fully account for the quantitative level of sleep induction observed in vivo. In addition, there are several mutants, of which kinase activities in cell lysates are not matched with the sleep phenotypes in vivo (e.g., T242D), suggesting the existence of an additional layer of regulation such as substrate specificity or protein localization. As for the S114D, this mutant showed a reduced expression level in 293T (S12B Fig). This result suggests the reduced protein stability at least in the 293T cells, which might lead to the sleep phenotype variation for this mutant.

Comparing phosphorylation-mimicking mutants and nonphosphorylation-mimicking mutants allowed us to attribute the effect of phosphorylation to the negative charge mimicked by the D residue or to any other effect caused by the mutation. As observed in the SIK3 phosphorylation site S551 [48], D and A mutations sometimes yield similar results (e.g., increased sleep), making it difficult to conclude that the D mutation mimics phosphorylation. For residues shown in Fig 6D, we showed that A and D mutants had different effects in sleep regulation in vivo, suggesting that the phosphorylation states of these sites in CaMKIIβ can regulate sleep.

Other than sleep control, phosphorylation-mimicking or nonphosphorylatable-mimicking mutants of CaMKII have been shown to exhibit defects in neuronal plasticity and some type of learning [49,50]. Because it is well understood that the sleep–wake cycle affects the learning process, CaMKIIβ-expressing mice with changes in sleep phenotype may also have changes in learning phenotype. In this case, it would be interesting to ask whether the changes in learning phenotype are simply due to sleep abnormalities. Alternatively, it is equally possible that the possible effect of CaMKIIβ mutants on neuronal plasticity is not just the functional output of sleep abnormalities, but changes in the sleep phenotypes are the behavioral phenotype associated with such changes in the neuronal functions.

The sleep-promoting effect observed with *Camk2a* promoter and *Vglut2-Cre* (Fig 3) suggests that CaMKIIβ promotes sleep by acting on excitatory neurons. However, these data do not exclude the possibility of the contribution of nonexcitatory neurons and glial cells for CaMKIIβ-dependent sleep regulation because the Cre-expression specificity may not be perfectly selective to desired cell types. Future research will have to precisely elucidate where CaMKIIβ exerts its sleep function in terms of both neuronal cell types and brain regions as well subcellular localization. It should be noted that focused expression of T287D to *Vglut2*-*Cre*-positive cells (Fig 3H) might induce the sleep maintenance activity (i.e., extended sleep duration and low *P*_*SW*_) in addition to the sleep induction activity (i.e., high *P*_*WS*_), suggesting that different types of neurons might be involved in the sleep induction or maintenance activities to different degrees. Notably, homeostatic regulation of sleep/wake-associated neuronal firing was recapitulated in cultured neuron/glial cells [51,52]. Given the ubiquitous and abundant expression of CaMKIIβ in neurons, investigating the relationship between sleep homeostasis in cultured neurons/glial cells and CaMKIIβ phosphorylation states would reveal valuable information about the ubiquitous and cell type–specific function of CaMKIIβ in the sleep control.

Multisite phosphorylation encodes complex biochemical systems such as the sequential triggering of multiple events and the integration of multiple signals (such as AND logic gates) [53,54]. We found that combining phosphomimetic mutations of T306D and T307D to T287D (i.e., T287D:T306D:T307D) does not affect sleep duration (compared with the T287D single mutation) but causes unexpected differences in sleep maintenance and sleep induction relationship (Fig 5). T287D mutant has a higher *P*_*WS*_, suggesting that T287 phosphorylation plays a role in sleep induction. On the other hand, the T287D:T306D:T307D mutant has a lower *P*_*SW*_, suggesting that T287/T306/T307 phosphorylation plays a role in sleep maintenance. It is well studied the sequential autophosphorylation from T287 to T306/T307 [19–21]. Thus, the expected sleep regulation sequence is physiologically reasonable: the increased transition rate from awake to sleep phase promoted by T287 phosphorylation, and then the induced sleep is stabilized by subsequent multisite T287/T306/T307 phosphorylation (Fig 6D). Indeed, Wang and colleagues phosphoproteomics analysis indicated that sleep deprivation increases the phosphorylation levels of both T287 and T306/T307 residues [4].

The autophosphorylation of T306 and T307 has a well-known inhibitory effect on the CaMKIIβ–CaM interaction [19,20]. CaMKIIβ del mutant shares some properties with T287D:T306D:T307D; both mutants lost the CaMKIIβ–CaM interaction and have the CaM-independent kinase activity. Nevertheless, T287D:T306D:T307D has the sleep maintenance activity while the del mutant shows sleep induction activity (Fig 2B). Thus, the mechanism of sleep maintenance by T287D:T306D:T307D may not be attributed to the loss of CaMKIIβ–CaM interaction itself. Recent studies suggested that the phosphorylation of T305/T306 of CaMKIIα promotes the dissociation of CaMKIIα dodecamer [55]. Another study demonstrated that the same phosphorylation promotes the translocation of CaMKIIα from the spine to dendrite [56]. It is plausible that different patterns of multisite phosphorylation or combination of D mutants of CaMKIIβ affect sleep induction/maintenance through the different interactions of endogenous CaMKIIα/CaMKIIβ and neuronal proteins. At least, the present study excludes the core circadian transcription repressors as downstream factors of CaMKIIβ sleep induction (Fig 4) and future research should be required for identifying downstream targets of CaMKIIβ in sleep control. Although our SRM method is not compatible with proteomics research, recent state-of-the-arts phosphoproteomics datasets would provide candidate phosphorylation substrates for the CaMKIIβ-dependent sleep promotion [4,5]. These datasets include known CaMKII’s substrate/binding proteins that show different phosphorylation levels in response to the sleep–wake cycle or sleep deprivation, such as NMDA receptors and Synapsin-1 protein. It will be also important to investigate the location in neurons where the CaMKII exerts its sleep-promoting effect.

We also showed that other residues (such as S26, S182, and T311) may contribute to the cancellation of sleep-promoting effect of CaMKIIβ (Fig 6D). S26 autophosphorylation occurs in CaMKIIγ [57] and suppresses the kinase activity potentially disrupting the interaction with ATP [58]. The expected loss of kinase activity is also supported by our cell lysate–based assay (S12C Fig). The other phosphoproteomics study identified S25 autophosphorylation in CaMKIIα [22]. Although the level of phosphorylation at S25 was indicated for approximately 5% of total CaMKIIα at 4-min incubation time [22], it is possible that the level of this phosphorylation continuously increases given the slow dynamics of autophosphorylation at S26 of CaMKIIβ found in this study. We also note that peptide phosphorylated with S26 can be found in vivo brain sample [4] (S13 Fig), although it is unable to distinguish CaMKII isoforms because of the identical sequence around the S26 phosphorylation site. The enhanced sleep promoting activity found in S26A:T287D:T306D:T307D versus T287D (Fig 6B) might reflect that part of the sleep canceling effect carried out by S26 phosphorylation is disrupted by S26A mutation. These lines of evidence suggest that loss of kinase activity elicited by S26 phosphorylation is involved in the physiological mechanisms that control the timing and/or the level of CaMKIIβ activity.

Reports suggest that T311 autophosphorylation occurs in CaMKIIβ [22] and that the phosphorylation level of the corresponding residue in CaMKIIα was reduced during the dark phase (mostly awake phase in mice) [3]. The T311 phosphorylation was also detected in the other set of phosphoproteomic analyses of in vivo mice brains [4], although sequence identity around the T311 residue makes it difficult to distinguish CaMKIIα and CaMKIIβ. These phosphoproteomics analyses support the possible role of phosphorylation at S26 or T311 in the regulation of CaMKIIα/CaMKIIβ in mice brains in vivo. Since S182 phosphorylation has not been detected in culture cells/in vivo partly because of the unsuitable sequence around the S182 for conventional trypsin-based proteomics technique, its physiological role is currently unclear. The reduced protein expression level of S182D mutant in 293T cells might be related to CaMKIIβ control based on the enhanced proteolysis. It should be tested whether the reduced S182D can be recovered by the inhibition of specific proteolysis pathways.

We should note that although our AAV-based screening covers every possible phosphorylation mimicking mutation for single mutants, the possible combinations of multisite phosphorylation in CaMKIIβ are far more numerous than those tested in this study. A pioneering dataset on daily cycling of synaptic phosphoproteins includes several residues spanning from 338 to 381 amino acids in CaMKIIβ [5]. These residues did not exhibit significant sleep phenotypes in our single/double-phosphomimetic screening but may have a role in sleep–wake control in other combinations of multisite phosphorylation of CaMKIIβ. In parallel with these sleep-controlling phosphorylation residues in CaMKIIβ, PP1 and PP2 have been shown to be involved in the dephosphorylation of CaMKIIα T286 phosphorylation in the postsynapse [59,60]. The role of such phosphatases in CaMKII-dependent sleep control needs to be investigated.

In summary, we propose that multisite phosphorylation status of CaMKIIβ controls different aspects of sleep-promoting effect of CaMKIIβ including sleep induction, maintenance, and potential cancellation effect of the sleep-promoting effect. Our results provide testable and quantifiable molecular candidates responsible for the induction and maintenance of sleep, which will be an important insight for the molecular validation of several models of sleep regulation. For example, a landmark idea of the synaptic homeostasis hypothesis assumes that neuronal excitation during the awake phase leads to increased synaptic connectivity, which, in turn, induces sleep [61,62]. However, in terms of mechanistic insight, it is still unclear why the awaking brain does not end up with saturated excitability and connectivity but turns into the sleep state. On the other hand, our study demonstrated that the kinase-active form (T287D) of CaMKIIβ has a sleep induction activity and the kinase activation marked by the phosphorylation of T287 residue is promoted by extended awake duration, providing a reasonable working hypothesis connecting the awake-associated neuronal activity and the induction of sleep. It should be rigorously tested whether the abovementioned CaMKIIβ residues play a role in the regulation of normal sleep regulation in vivo through the knockout/knockdown rescue experiment by reexpressing the nonphosphorylatable A mutations at corresponding residues. Through such rescue experiments, it would be possible to approach the question not covered by the current study: Whether the sleep cancellation effect is related to the transition from sleep to awake phase in the natural sleep–wake cycle or the cancellation of additional sleep needs upon unusual input such as sleep deprivation.

## Materials and methods

### Plasmids

Mouse *Camk2b* cDNA (NM_007595) was subcloned into the pMU2 vector [63] that expresses genes under the CMV promoter. The cloned sequence corresponds to the β variant with 94 residue linker (exon 13, 14b, 16, 17, 18). Note that the FLAG-tag involved in the original pMU2 vector was removed in the construct used in this study. Mutagenesis of pMU2-*Camk2b* was conducted by inverse PCR with Mighty Cloning Reagent Set (Blunt End) (Takara Bio, Japan) following to the manufacturer’s protocol.

For pAAV construction, the *Camk2b* sequence was transferred into the pAAV vector (kindly provided by Dr. Hirokazu Hirai) along with the *hSyn1* promoter [64], FLAG tag, *Camk2b* 3′ UTR, WPRE, and SV40 polyA sequences as illustrated in Fig 1B. For the *Camk2b* 3′ UTR used in this study, the evolutionarily conserved approximately 350 bp (chr11:5,971,489–5,971,827, GRCm38/mm10) and approximately 650 bp (chr11:5,969,672–5,970,313, GRCm38/mm10) regions in the mouse *Camk2b* 3′ UTR was cloned and assembled tandemly. For DIO constructs, the inverted FLAG-*Camk2b* sequence flanked by lox2272 and loxP was inserted between the *hSyn1* promoter and the *Camk2b* 3′ UTR of the pAAV vector as illustrated in Fig 3G. For more targeted gene expression, the *hSyn1* promoter was replaced with other promoters. A vector containing *Camk2a* promoter sequence was a kind gift from Drs. Masamitsu Iino and Yohei Okubo (The University of Tokyo). For the *Camk2b* promoter, approximately 1,300 bp region (chr11:6,065,706–6,066,972, GRCm38/mm10) upstream of the TSS of the *Camk2b* gene was cloned using a pair of primers (5′-AGCACTCTGTCAAATGTACCTTTAG-3′; 5′-AGATCTGCTCGCTCTGTCCC-3′) as illustrated in S9D Fig.

The mCherry-AIP2 was constructed by fusing the AIP2 sequence (KKKLRRQEAFDAL) to the C-terminus of mCherry via a (GGGGS)x3 linker. To construct pAAV, the mCherry-AIP2 sequences were inserted into the pAAV vector with the *hSyn1* promoter, dendritic targeting element (DTE) of mouse *Map2* gene, WPRE, and SV40 polyA sequences. The DTE of *Map2* were amplified and cloned from C57BL/6N mouse genomic DNA [65].

pUCmini-iCAP-PHP.eB for PHP.eB production was a gift from Dr. Viviana Gradinaru (Addgene plasmid # 103005).

### Animals and sleep phenotyping

All experimental procedures and housing conditions were approved by the Institutional Animal Care and Use Committee of RIKEN Center for Biosystems Dynamics Research and the University of Tokyo. All the animals were cared for and treated humanely in accordance with the Institutional Guidelines for Experiments using Animals. All mice had ad libitum access to food and water, and were maintained at ambient temperature and humidity conditions under a 12-h LD cycle. The timing of switching from dark to light environment was set as ZT0. All C57BL/6N mice were purchased from CLEA Japan (Tokyo, Japan). The mice used in each experiment were randomly chosen from colonies. EEG/EMG recording for the *Camk2b* KO mice were conducted at the University of Tokyo. Other animal experiments were performed in the RIKEN Center for Biosystems Dynamics Research.

### Mass spectrometry and western blotting of mice brain samples

C57BL/6N mice (CLEA Japan, Japan) were housed in a light–dark controlling rack (Nippon Medical & Chemical instruments, Japan) and habituated to a 12-h LD cycle for at least 1 week. At 8 weeks old, half of the mice were subjected to the sleep deprivation protocol from ZT0 to ZT6. The sleep deprivation was conducted by gentle handling and cage changing [28] at every 2 h. The other mice were housed under ad lib sleep conditions. At ZT6, the mice were killed by cervical dislocation and their forebrain was immediately frozen in liquid nitrogen. The brain samples were stored at −80 °C. The frozen brains were cryo-crushed with a Coolmil (Tokken, Japan) precooled in liquid nitrogen, and the brain powders were stored at −80 °C.

The brain powders were then lysed and digested according to the phase-transfer surfactant (PTS) method [66]. Approximately 10 mg of brain powder was added to the 500 μL of Solution B (12 mM sodium deoxycholate, 12 mM N-lauroylsarcosine sodium salt, 50 mM ammonium hydrogen carbonate) containing phosphatase inhibitors (1 mM sodium orthovanadate, 1 mM β-glycerophosphoric acid disodium salt pentahydrate, 4 mM sodium (+)-tartrate dihydrate, 2.5 mM sodium fluoride, 1.15 mM disodium molybdate (VI) dihydrate) preheated at 98 °C and sonicated extensively. After further incubation at 98 °C for 30 min, the samples were reduced with 10 mM dithiothreitol (FUJIFILM Wako Pure Chemical, Japan) at room temperature for 30 min, and then alkylated with 100 mM iodoacetamide (Sigma-Aldrich, USA) at room temperature for 30 min. The samples were then diluted to 5-fold by adding Solution A (50 mM ammonium hydrogen carbonate) and digested them by adding 5 μg of lysyl endopeptidase (Lys-C) (FUJIFILM Wako Pure Chemical, Japan). After 37 °C overnight incubation, 5 μg of trypsin (Roche, Switzerland) was added and the mixture was further incubated at 37 °C overnight. After the digestion, an equal volume of ethyl acetate was added to the sample, which was acidified with 0.5% TFA and well mixed to transfer the detergents to the organic phase. The sample was then centrifuged at 2,380 × *g* for 15 min at room temperature, and an aqueous phase containing peptides was collected and dried with a SpeedVac (Thermo Fisher Scientific, USA).

The dried peptides were solubilized in 1 mL of 2% acetonitrile and 0.1% TFA. We prepared an internal control by mixing 500 μL of each peptide solution. The individual samples were the remaining 500 μL of each peptide solution. The internal control and individual samples were trapped and desalted on a Sep-Pak C18 cartridge (Waters, USA). Dimethyl-labeling was then applied to the peptides on the cartridge as previously described [67]. Formaldehyde (CH_2_O, Nacalai Tesque, Japan) and NaBH_3_CN (Sigma-Aldrich, USA) were added to the individual samples (light label), and isotope-labeled formaldehyde (CD_2_O, Cambridge Isotope Laboratories, USA) and NaBH_3_CN (Sigma-Aldrich, USA) were added to the internal control mixture (medium label). The dimethyl-labeled peptides on the Sep-Pak cartridge were eluted with an 80% acetonitrile and 0.1% TFA solution. Then, equal amount of medium-labeled internal control mixture was added to each light-labeled individual sample. This allowed us to compare the relative amount of peptides in the individual samples with each other using the equally added medium-labeled internal control mixture as a standard.

A one-hundredth of the mixture underwent LC-MS analysis to quantify the amount of CaMKIIα/β and total proteins. The remaining mixture was applied to High-Select Fe-NTA Phosphopeptide Enrichment Kit (Thermo Fisher Scientific, USA) to enrich the phosphorylated peptides following the manufacturer’s protocol.

All analytical samples were dried with a SpeedVac (Thermo Fisher Scientific, USA) and dissolved in 2% acetonitrile and 0.1% TFA. Mass spectrometry–based quantification of CaMKIIα/β-derived peptides was carried out by SRM analysis using a TSQ Quantiva triple-stage quadrupole mass spectrometer (Thermo Fisher Scientific, USA). The following parameters were selected: positive mode, Q1 and Q3 resolutions of 0.7 full width of half maximum (FWHM), cycle time of 2 s, and gas pressure of 1.5 Torr. The mass spectrometer was equipped with an UltiMate 3000 RSLC nano nano-high-performance liquid chromatography (HPLC) system (Thermo Fisher Scientific, USA), and a PepMap HPLC trap column (C18, 5 μm, 100 A; Thermo Fisher Scientific, USA) for loading samples. Samples were separated by reverse-phase chromatography using a PepMap rapid separation liquid chromatography (RSLC) EASY-Spray column (C18, 3 μm, 100 A, 75 μm × 15 cm; Thermo Fisher Scientific, USA) using mobile phases A (0.1% formic acid/H2O) and B (0.1% formic acid and 100% acetonitrile) at a flow rate of 300 nl/min (4% B for 5 min, 4% to 35% B in 55 min, 35% to 95% B in 1 min, 95% B for 10 min, 95% to 4% B in 0.1 min, and 4% B for 9.9 min). The eluted material was directly electrosprayed into the MS. The SRM transitions of the target peptides were determined based on the preanalysis of several samples including mice brains, 293T cells expressing CaMKIIβ, and synthesized peptides, and optimized using Pinpoint software, version 1.3 (Thermo Fisher Scientific, USA) (S2 and S3 Tables). The Quan Browser of the Quan Browser data system, version 3.0.63 (Thermo Fisher Scientific, USA) was used for data processing and quantification.

To estimate the relative amount of total peptides involved in each brain sample, approximately half of the light/medium mixture sample without the enrichment of phosphopeptides was analyzed by data-dependent MS/MS with a mass spectrometer (Q-Exactive Mass Spectrometer, Thermo Fisher Scientific, USA) equipped with an HPLC system containing nano HPLC equipment (Advance UHPLC, Bruker Daltonics, USA) and an HTC-PAL autosampler (CTC Analytics, Switzerland) with a trap column (0.3 × 5 mm, L-column, ODS, Chemicals Evaluation and Research Institute, Japan). An analytical sample was loaded into the LC-MS system to be separated by a gradient using mobile phases A (0.1% formic acid) and B (0.1% formic acid and 100% acetonitrile) at a flow rate 300 nL/min (4% to 32% B in 190 min, 32% to 95% B in 1 min, 95% B for 2 min, 95% to 4% B in 1 min, and 2% B for 6 min) with a homemade capillary column (200 mm length, 100 μm inner diameter) packed with 2 μm C18 resin (L-column2, Chemicals Evaluation and Research Institute, Japan). The eluted peptides were then electrosprayed (1.8 to 2.3 kV) and introduced into the MS equipment (positive ion mode, data-dependent MS/MS). MS data were analyzed by Proteome Discoverer version 2.2 (Thermo Fisher Scientific, USA) with the Swiss-Prot section of UniProtKB mouse database (as of August 9, 2018). The relative amount of CaMKIIα/β protein was normalized to the median of all quantified proteins for each sample, with the effect derived from different amounts of start materials being excluded.

For the western blotting analysis, brain powder was lysed in the 3× Laemmli sample buffer (20% glycerol, 2.25% sodium dodecyl sulphate (SDS), 187.5 mM Tris-HCl at pH 6.8, 0.015% bromophenol blue) preheated at 98 °C and sonicated extensively. Approximately 0.1 mg of brain powder (approximately 10 μg protein) was subjected to each lane of handmade polyacrylamide gel. The samples were separated by SDS–polyacrylamide gel electrophoresis and then transferred to a polyvinylidene difluoride (PVDF) membrane (Hybond-P PVDF membranes, Merck, Germany) by a wet-transfer apparatus (Vep-3, Thermo Fisher Scientific, USA). The membrane was washed by TTBS (0.9% NaCl, 0.1% Tween-20, and 100 mM Tris-HCl at pH 7.5) and nonspecific protein binding was blocked by incubating with Blocking One solution (Nacalai Tesque, Japan) for 1 h at room temperature. FLAG-tagged protein was detected by an anti-FLAG M2 antibody conjugated with horseradish peroxidase (A8592, Sigma-Aldrich, USA). CaMKIIβ and α-Tublin were detected by primary antibodies anti-CaMK2β (#139800, Thermo Fisher Scientific, USA) or anti-alpha Tubulin [DM1A] (ab7291, Abcam), respectively, followed by the incubation with a secondary antibody anti-mouse IgG HRP conjugate (W4021, Promega, USA). All the primary antibodies were diluted 1/3,000 in 10% Blocking One/TTBS (50 mM Tris, 0.5 M NaCl, 0.05% Tween-20 (pH 7.4)) and incubated with the membrane for overnight at 4 °C. The secondary antibody was diluted 1/2,000 in 10% Blocking One/TTBS (50 mM Tris, 0.5 M NaCl, 0.05% Tween-20 (pH 7.4)) and incubated with the membrane for 1 h at room temperature. Immunoreactivities were detected with Clarity Western ECL Substrate for Chemiluminescent Western Blot Detection (Bio-rad, U.S.A.) and ChemiDoc XRS+ system (Bio-rad, USA).

### Tissue clearing and LSFM imaging

AAV-administrated mice were perfusion fixed under anesthesia, and brains were isolated. Isolated brains were fixed overnight in 4% PFA and then washed with PBS. For clearing the mouse brain, second-generation CUBIC protocols were used. The detailed protocol can be found in a previous report [68]. For delipidation, the brain was treated with CUBIC-L (10% (w/w) N-butyldiethanolamine and 10% (w/w) Triton X-100) solution at 37 °C for 5 days. For nuclear staining, the brain was rinsed with PBS and incubated in 1:250 diluted RedDot2 (Biotium, 40061) in staining buffer (10% (w/w) Triton X-100, 10% (w/w) Urea, 5% (w/w) *N*,*N*,*N′*,*N′*-Tetrakis(2-hydroxypropyl)ethylenediamine, 500 mM NaCl) for 3 days at 37 °C. The stained brain sample was washed with PBS and then treated in CUBIC-R+ solution (45% (w/w) antipyrine, 30% (w/w) nicotinamide, 0.5% (v/v) *N*-butyldiethanolamine) for 3 days at 25 °C for RI matching. For whole-brain imaging, the cleared brain sample was embedded in a CUBIC-R+ gel, which contains 2% (w/w) agarose in the CUBIC-R+ solution and set in a customized light sheet microscopy (LSFM) [68]. Dual-colored images were simultaneously acquired with illumination objective lens (MVPLAPO 1×, Olympus, Japan), 10× detection objective lens (XLPLN10XSVMP, Olympus, Japan), Dichroic mirror (DMSP650L, Thorlabs, USA) and following laser and fluorescence filters: RedDot2 [Ex: 594 nm, Em: 700 nm bandpass (FB700-40, Thorlabs, USA)], mCherry [Ex: 594 nm, Em: 625 nm bandpass (ET625/30m, Chroma Technology, USA)]. Stacked brain images were reconstructed and visualized by the Imaris software (Bitplane).

### Mass spectrometry of purified CaMKIIβ

The spike peptides were synthesized for each target phosphorylated peptide with a peptide synthesizer Syro Wave (Biotage, Sweden) using Fmoc solid-phase chemistry. The synthesized phosphorylated peptides were treated with dithiothreitol and iodoacetamide as described above. The peptides were desalted by using handmade C18 StageTips [69]. The desalted peptides on the StageTips were subjected to dimethyl-labeling with isotope-labeled formaldehyde (^13^CD_2_O, ISOTEC, USA) and NaBD_3_CN (Cambridge Isotope Laboratories, USA) (heavy label) as described previously [67]. The dimethyl-labeled spike peptides were eluted with an 80% acetonitrile and 0.1% TFA solution and dried with a SpeedVac (Thermo Fisher Scientific, USA).

For the time course sampling for autophosphorylation detection, one time point sample contains 0.3 μM purified GST-CaMKIIβ protein (Carna Biosciences, Japan), 50 mM HEPES-NaOH (pH 7.6), 150 mM NaCl, 1 mM MgCl_2_, 0.25% (v/v) NP-40, and 2.5 mM ATP. The sample without CaM was sampled and used as “0 min” time point. Then, 0.5 mM CaCl_2_ and 10 mM EGTA were added to the indicated conditions shown in S12D Fig. The kinase reaction was initiated by adding 0.5 μM CaM to each sample. During the time course sampling, 10 mM EGTA was added for the condition named “0.5 mM Ca^2+^, 10 mM EGTA at 5 min (condition #4)”. Note that for the quantification of S182 phosphorylation, 10-fold higher concentration of purified GST-CaMKIIβ and CaM were used because of the low signal sensitivity of the corresponding phosphorylated peptide.

The kinase reaction was terminated by adding an equal volume of Solution B and incubating at 98 °C for 30 min. The samples were reduced, alkylated, and digested by proteases according to the PTS method [66] as described above except that 1 μg of Lys-C and 1 μg of trypsin were used for most of the samples, and 1 μg of Lys-C and 1 μg of Glu-C (Promega, USA) were used for the sample for quantifying S182 phosphorylation.

The dried peptides were solubilized in 1 mL of a 2% acetonitrile and 0.1% TFA solution and trapped on C18 StageTips [69]. The trapped peptides were subjected to dimethyl-labeling with formaldehyde (light label) as described above. An additional GST-CaMKIIβ sample independent from the time course sampling were prepared as an internal control reference for nonphosphorylated peptides and subjected to dimethyl-labeling with CD_2_O (medium label). The dimethyl-labeled peptides on the tip were eluted with an 80% acetonitrile and 0.1% TFA solution. Then, 1/30 volume of the light-labeled samples were isolated and mixed with equal amounts of medium label peptides. This allowed us to compare the relative amount of GST-CaMKIIβ in the individual time course samples with each other using the medium-labeled internal control.

The remainder of the light-labeled samples were mixed with the mixture of heavy labeled spike phosphorylated peptides and applied to High-Select Fe-NTA Phosphopeptide Enrichment Kit (Thermo fisher Scientific, USA) to enrich the phosphorylated peptides. This allowed us to compare the relative amount of phosphorylated peptides in the individual time course samples with each other using the heavy labeled spike peptides.

All analytical samples were dried with a SpeedVac (Thermo Fisher Scientific, USA) and dissolved in a 2% acetonitrile and 0.1% TFA solution. Mass spectrometry–based quantification was carried out by SRM analysis using a TSQ Quantiva triple-stage quadrupole mass spectrometer (Thermo Fisher Scientific, USA) as described above. The amount of each phosphorylated peptide was normalized to the amount of total GST-CaMKIIβ quantified using the average amounts of several nonphosphorylated peptides.

### Kinase assay using CaMKIIβ-expressed 293T lysates

293T cells were grown in a culture medium consisting of Dulbecco’s Modified Eagle Medium (DMEM) (high glucose, Thermo Fisher Scientific), 10% FBS (Sigma-Aldrich) and 100 U/ml penicillin-streptomycin (Thermo Fisher Scientific) at 37 **°**C with 5% CO_2_. The cells were plated at 2 × 10^4^ cells per well in 24-well plates 24 h before transfection. The cells in each well were transfected with 1.6 μg PEI (Polyethylenimine, Linear, MW 25000, Polysciences) and 400 ng of pMU2-*Camk2b* plasmids. The medium in each well was replaced with fresh culture medium 24 h after the transfection. The cells were stayed for another 48 h and collected by removing all the culture medium. The remaining cells on the 24-well plate were stored at **−**80 °C.

The cells were lysed with 200 μL of cell lysis buffer (50 mM HEPES-NaOH (pH 7.6), 150 mM NaCl, 0.5 mM CaCl_2_, 1 mM MgCl_2_, and 0.25% (v/v) NP-40) containing protease inhibitors (100 mM phenylmethanesulfonyl fluoride, 0.1 mM Aprotinin, 2 mM Leupeptin hemisulfate, 1 mM Pepstatin A, and 5 mM Bestatin). Followed by extensive sonication, the cell lysates were collected and stored at **−**80 °C.

The relative expression levels of CaMKIIβ in each cell lysate were estimated by dot blot. A PVDF membrane (Hybond-P PVDF membranes, Merck) was immersed in 100% methanol (Nacalai Tesque) and then soaked in water for at least 10 min. Excess water was removed from the membrane; 2 μL of cell lysate was spotted on the membrane. The membrane was then dried completely, immersed in 100% Methanol, and equilibrated in water. The membrane was incubated in Blocking One solution (Nacalai Tesque) for 1 h at room temperature. After the blocking reaction, the membrane was incubated for 2 h with the primary antibody anti-CaMK2β (#139800, Thermo Fisher Scientific) diluted at 1/3,000 in 10% Blocking One/TTBS (50 mM Tris, 0.5 M NaCl, 0.05% Tween-20 (pH 7.4)). The membrane was washed with TTBS and incubated for 1 h with the second antibody anti-mouse IgG HRP conjugate (W4021, Promega) diluted at 1/3,000 in 10% Blocking One/TTBS. Immunoreactivities of the blotted proteins were detected with Clarity Western ECL Substrate for Chemiluminescent Western Blot Detection (Bio-rad) and ChemiDoc XRS+ system (Bio-rad). The images were analyzed with Image Lab software (version 6.01, Bio-rad). For each dot blot experiment, serial dilution of cell lysate expressing the WT CaMKIIβ was spotted to confirm that the quantification of the dot blot signal was within the linear range of detection.

The kinase activity of CaMKIIβ-expressed cell lysate was calibrated as follows. First, a serial dilution of cell lysate expressing WT CaMKIIβ was prepared. Then, 5 μL of each diluted cell lysate was mixed with 15 μL of cell lysis buffer containing 0.33 mM ATP and 5 μM ProfilerPro Kinase Peptide Substrate 11 5-FAM-KKLNRTLSVA-COOH (PerkinElmer) in the presence or absence of 0.66 μM CaM (Sigma-Aldrich). After incubating at 37 °C for 10 min, the reaction was stopped by incubating at 98 °C for 10 min. A volume of 100 μL of 2% ACN/0.1% TFA was added to the reaction mixture, and the mixture was analyzed by mobility shift assay (LabChip EZ Reader II; PerkinElmer). The kinase activity is the percentage of phosphorylated peptide signal over the total substrate peptide signal. Based on the kinase activity obtained from the serial dilution of cell lysate, we determined a dilution rate of cell lysate that gives the approximately 50% kinase activity in the calibration curve in the presence of CaM.

Using the dot blot data, the cell lysate expressing each mutant CaMKIIβ was diluted so that the relative expression levels between WT and mutant CaMKIIβ are to be the same. A lysate of the cells treated with the PEI transfection procedure without vector plasmid was used to dilute each CaMKIIβ-expressed cell lysate. Thus, the background kinase activity, if any, originated from endogenous 293T proteins would be the same among cell lysates. As for the mutants with 25% or lower expression levels compared to WT CaMKIIβ, we could not adjust the relative expression level compared to WT, and mutant cell lysates without dilution were used for the kinase assay.

The quantification of kinase activity was carried out by mixing 5 μL of cell lysates (diluted as described above) and 15 μL of cell lysis buffer containing 0.33 mM ATP and 5 μM ProfilerPro Kinase Peptide Substrate 11 5-FAM-KKLNRTLSVA-COOH (PerkinElmer), in the presence or absence of 0.66 μM CaM (Sigma-Aldrich). After incubating at 37 °C for 10 min, the reaction was stopped by incubating at 98 °C for 10 min. A volume of 100 μL of 2% ACN/0.1% TFA was added to the reaction mixture, and the mixture was analyzed by mobility shift assay (LabChip EZ Reader II and operation software version 2.2.126.0; PerkinElmer).

### Mass spectrometry of CaMKIIβ expressed in 293T cells

293T cells expressing CaMKIIβ WT, T287D, or K43R:T287D were prepared as described in the “Kinase assay using CaMKIIβ-expressed 293T lysates” section except for that 1 × 10^5^ 293T cells were plated in a 35-mm dish and transfected with 8 μg PEI (Polyethylenimine, Linear, MW 25000, Polysciences) and 2 μg of pMU2-*Camk2b* plasmids.

The SRM-based mass spectrometry analysis was carried out the similar process described in the “Mass spectrometry and western blotting of mice brain samples” section. In brief, 200 μL of Solution B containing phosphatase inhibitors was added to the 293T cells cultured in a 35-mm dish. The samples were reduced, alkylated, and then digested by 2 μg of lysyl endopeptidase (Lys-C) (FUJIFILM Wako Pure Chemical, Japan) followed by further digestion by 2 μg of trypsin (Roche, Switzerland).

The dried peptides were solubilized in 200 μL of 2% acetonitrile and 0.1% TFA. We prepared an internal control by mixing 100 μL of each peptide solution. The individual samples were the remaining 100 μL of each peptide solution. The internal control and individual samples were desalted and subjected to dimethyl-labeling on C18 StageTips [69]. Light label was applied to the individual sample, and medium label was applied to the internal control. An equal amount of medium-labeled internal control mixture was added to each light-labeled individual sample. A one-tenth of the mixture underwent LC-MS analysis to quantify the amount of expressed CaMKIIβ. The remaining mixture was applied to High-Select Fe-NTA Phosphopeptide Enrichment Kit (Thermo Fisher Scientific, USA) to enrich the phosphorylated peptides following the manufacturer’s protocol. Mass spectrometry–based quantification of CaMKIIβ-derived peptides was carried out by SRM analysis using a TSQ Quantiva triple-stage quadrupole mass spectrometer (Thermo Fisher Scientific, USA).

### Production of *Camk2b* KO mice

*Camk2b* KO mice were generated using the triple-target CRISPR method described previously [16]. C57BL/6N females (4 to 6 weeks old, CLEA Japan, Japan) were superovulated and mated with C57BL/6N males (CLEA Japan, Japan). The fertilized eggs were collected from the ampulla of the oviduct of plugged C57BL/6N females by microdissection and kept in KSOM medium (Merck, Germany or ARK Resource, Japan) in a 5% CO_2_ incubator at 37 °C. The design of gRNAs for *Camk2b* was previously shown as set 1 in a previous study [2]. In the previous study, an independent set of gRNA called set 2 was also tested. A significant decrease in the sleep duration was observed both in set 1 and set 2 gRNA-injected mice, suggesting that at least a major part of sleep phenotype is not due to the off-target effect of injected gRNAs [2]. The synthesized gRNAs for *Camk2b* (150 ng/μL in total) and *Cas9* mRNA (100 ng/μL) were coinjected into the cytoplasm of fertilized eggs in M2 medium (Merck, Germany or ARK Resource, Japan) at room temperature. After microinjection, the embryos were cultured for 1 h in KSOM medium (Merck, Germany, or ARK Resource, Japan) in a 5% CO_2_ incubator at 37 °C. Approximately 15 to 30 embryos were then transferred to the oviducts of pseudopregnant female ICR mice.

Genotyping of KO mice was conducted with the same protocol described previously [16]. qPCR was performed using genomic DNA purified from tails of WT and KO mice and primers, which were annealed to the target sequences. The target site abundance was calculated using a standard curve obtained from WT genomic DNA. The amount of *Tbp* [70] was quantified with a pair of primers (5′-CCCCCTCTGCACTGAAATCA-3′; 5′-GTAGCAGCACAGAGCAAGCAA-3′) and used as an internal control. When the amplified intact DNA by qPCR is less than 0.5% of WT genome, we judged that the target DNA is not detectable. When any of 3 targets was not detected, we classified the animal as a KO. When we could not confirm KO genotype by the qPCR, we performed another qPCR using the alternative primer pairs, which was independent of the initial qPCR. In the case of *Camk2b* KO used in this study, first and second targets of triple CRISPR gRNA were judged as not detectable by another qPCR. The result of qPCR is shown in S8A Fig, and the primer list used for the qPCR is shown below.

qPCR primer pairs #1:

Target #1
  - Forward: 5′-CCACAGGGGTGATCCTGTATATCCTGC-3′
  - Reverse: 5′-CTGCTGGTACAGCTTGTGTTGGTCCTC-3′
Target #2
  - Forward: 5′- GGAAAATCTGTGACCCAGGCCTGAC-3′
  - Reverse: 5′- TCTGTGGAAATCCATCCCTTCGACC-3′
Target #3
  - Forward: 5′- GAACCCGCACGTGCACGTCATTGGC-3′
  - Reverse: 5′- CCCTGGCCATCGATGTACTGTGTG-3′

qPCR primer pairs #2:

Target #1
  - Forward: 5′- CAGAAAGGTGGGTAGCCCACCAGCAGG-3′
  - Reverse: 5′- CTATGCTGCTCACCTCCCCATCCACAG-3′
Target #2
  - Forward: 5′- GCCTGAAGCTCTGGGCAACCTGGTCG-3′
  - Reverse: 5′- CCACCCCAGCCTTTTCACTCACGGTTCTC-3′
Target #3
  - Forward: 5′- GCATCGCCTACATCCGCCTCACAC-3′
  - Reverse: 5′- CGGTGCCACACACGGGTCTCTTCGGAC-3′

### Production of *Camk2b*^*FLAG/FLAG*^ mice

FLAG-tag sequence was inserted into the endogenous *Camk2b* locus (prior to the stop codon) by single-stranded oligodeoxynucleotide (ssODN) and CRISPR/Cas9-mediated knock-in. The gRNA target sequence and a donor sequence were selected according to previous study [71]. Preparation of gRNA and Cas9 and general procedures for obtaining the genetically modified mouse were conducted according to previous study [2]. Following primer sequences were used to produce gRNA targeting the *Camk2b* locus.

*Camk2b-*FLAG gRNA primer forward #1

- 5′-CACTATAGGCAGTGGCCCCGCTGCAGTGGTTTTAGAGCTAGAAATAGC -3′

*Camk2b-*FLAG gRNA primer forward #2

- 5′- GGGCCTAATACGACTCACTATAGGCAGTGGCCCCGCTGCAGTGG -3′

*Camk2b-*FLAG gRNA primer reverse #1

- 5′- AAAAGCACCGACTCGGTGCC -3′
- A donor ssODN (*sequence*) was synthesized by Integrated DNA Technologies.

*Camk2b-*FLAG ssODN (capital letter: FLAG tag sequence)

- 5′- aagagacccgtgtgtggcaccgccgcgacggcaagtggcagaatgtacatttccactgctcgggcgct
- ccagtggccccgctgcagGACTACAAGGACGACGATGACAAGtgaggtgagtccctgcggt
- gtgcgtagggcagtgcggcatgcgtgggacagtgcagcgtgcatggggtgtggcccagtgcagcgtgc -3′

Approximately 1 to 2 pL of RNase free water (**Nacalai Tesque)** containing 100 ng/μl gRNA, 100 ng/μl Cas9 mRNA, and 100 ng/μl ssODN was injected into the cytoplasm of fertilized eggs in M2 medium (Merck, Germany or ARK Resource, Japan) at room temperature. After microinjection, the embryos were cultured for 1 h in KSOM medium (Merck, Germany, or ARK Resource, Japan) in a 5% CO_2_ incubator at 37 °C. Approximately 15 to 30 embryos were then transferred to the oviducts of pseudopregnant female ICR mice.

Genomic DNA of F_0_ mice tails was extracted with NucleoSpin Tissue kit (Takara Bio, Japan) according to the manufacturer’s protocol. The genotyping PCR was conducted by using following primer pairs to select heterozygous or homozygous FLAG knock-in offspring. Genotyping was based on the size and direct sequencing of the PCR amplicon. The obtained heterozygous or homozygous FLAG knock-in F_0_ mice were crossed with wildtype C57BL/6N mice to obtain heterozygous FLAG knock-in F_1_ mice.

*Camk2b*-FLAG genotyping primer pairs:

Pair #1
  - Forward: 5′- ACGACCAACTCCATTGCTGAC -3′
  - Reverse: 5′- CTACATCCGCCTCACACAGTACATC -3′
Pair #2
  - Forward: 5′- ACGACCAACTCCATTGCTGAC -3′
  - Reverse: 5′- GACTACAAGGACGACGATGACAAG -3′
Pair #3
  - Forward: 5′- CTTGTCATCGTCGTCCTTGTAGTC -3′
  - Reverse: 5′- CTACATCCGCCTCACACAGTACATC -3′

### Sleep measurement with the SSS

The SSS system enables fully automated and noninvasive sleep/wake phenotyping [16]. The SSS recording and analysis were carried out according to the protocol described previously [16]. The light condition of the SSS rack was set to LD (12 h periods) or DD. Mice had ad libidum access to food and water. In the normal measurement, 8-week-old mice were placed in the SSS chambers for 1 to 2 weeks for sleep recordings. For data analysis, we excluded the first day and used 6 days of measurement data. For the *Cry1/2* DKO and *Per1/2* DKO mutant mice, recordings were performed under LD conditions for 2 weeks followed by DD conditions for 2 weeks. For data analysis, we excluded the first day and used 4 days of measurement data under each light condition. Sleep staging was performed in every 8-s epoch.

Sleep parameters, such as sleep duration, *P*_*WS*_, and *P*_*SW*,_ were defined previously [16]. In the SSS, sleep staging was performed every 8 s, which is the smallest unit called “epoch.” When we focus on 2 consecutive epochs, there are 4 combinations: keeping awake state (wake to wake), keeping sleep state (sleep to sleep), transition from wakefulness to sleep (wake to sleep), and transition from sleep to wakefulness (sleep to wake). Transition probabilities were calculated from all 2 consecutive epochs in the measurement period. The definition of transition probabilities are as follows: *P*_*WS*_ (transition probability from wake to sleep) is defined as *P*_*WS*_ = *N*_*WS*_ / (*N*_*WS*_ + *N*_*WW*_), and *P*_*SW*_ (transition probability from sleep to wake) is defined as *P*_*SW*_ = *N*_*SW*_ / (*N*_*SW*_ + *N*_*SS*_), where *N*_*mn*_ is the number of transitions from state m to n (m, n ∈ {sleep, wake}) in the observed period. The balance between *P*_*WS*_ and *P*_*SW*_ determines the total sleep time, i.e., mice with longer sleep time tend to have increased *P*_*WS*_ and/or decreased *P*_*SW*_. *P*_*WS*_ and *P*_*SW*_ are independent of each other, and it can be deduced from the definition that *P*_*WS*_ + *P*_*WW*_ = 1 and *P*_*SW*_ + *P*_*SS*_ = 1. The sleep episode duration is the average of the time spent in each consecutive sleep phase during the observed period.

### Sleep measurement with EEG/EMG recording

For EEG/EMG recording, AAV-administrated 6-week-old C57BL/6N mice were used for surgery. For the recording of *Camk2b* KO mice, 16- to 17-week-old *Camk2b* KO mice and WT control mice at the same age were used for surgery. Wired and wireless recording method are used in parallel for EEG/EMG measurements, and we have confirmed that these 2 methods give qualitatively comparable results.

For wireless recordings, anesthetized mice were implanted a telemetry transmitter (DSI, USA). As EEG electrodes, 2 stainless steel screws were connected with lines from the transmitter and embedded in the skull of the cortex (anteroposterior, +1.0 mm; right, +1.5 mm from bregma or lambda). As EMG electrodes, 2 lines from the transmitter were placed in the trapezius muscles. After the surgery, the mice were allowed to recover for at least 10 days. EEGs and EMGs were recoded wirelessly. The mice had access to food and water. The sampling rate was 100 Hz for both EEG and EMG. The detailed methods were described previously [72].

For wired recordings, mice were implanted with EEG and EMG electrodes for polysomnographic recordings. To monitor EEG signals, 2 stainless steel EEG recording screws with 1.0 mm in diameter and 2.0 mm in length were implanted on the skull of the cortex (anterior, +1.0 mm; right, +1.5 mm from bregma or lambda). EMG activity was monitored through stainless steel, Teflon-coated wires with 0.33 mm in diameter (AS633, Cooner Wire, California, USA) placed into the trapezius muscle. The EEG and EMG wires were soldered to miniature connector with 4 pins in 2 mm pitch (Hirose Electric, Japan). Finally, the electrode assembly was fixed to the skull with dental cement (Unifast III, GC Corporation, Japan). After 10 days of recovery, the mice were placed in experimental cages with a connection of spring supported recording leads. The EEG/EMG signals were amplified (Biotex, Japan), filtered (EEG, 0.5 to 60 Hz; EMG, 5 to 128 Hz), digitized at a sampling rate of 128 Hz, and recorded using VitalRecorder software (KISSEI Comtec, Japan).

For the sleep staging, we used the FASTER method [72] with some modifications to automatically annotate EEG and EMG data. A total of 24 h of recording data were used for the analysis. Sleep staging was performed every 8-s epoch. Finally, the annotations were manually checked.

The power spectrum density was calculated for each epoch by fast Fourier transformation (FFT) with Welch’s averaging method. Briefly, each 8-s segment was further divided into 8 overlapping sequences. The overlapping length was 50% of each sequence. The Hamming window was applied onto the sequences before the FFT, and the obtained spectrum was averaged over the 8 sequences. The dirty segments were excluded from the subsequent processes [72]. The power spectrum of each behavioral state (Wake, NREM, and REM) was calculated by averaging the power spectra (1 to 50 Hz) of segments within each state over the observation period. The calculated power spectra were normalized by the total power. The power density in typical frequency domains was calculated as the summation of the powers in each frequency domain (slow, 0.5 to 1 Hz; delta, 0.5 to 4 Hz; theta, 6 to 10 Hz).

Transition probabilities between wakefulness, NREM sleep, and REM sleep were calculated same as previously reported [73]. For example, *P*_*NW*_ = *N*_*NW*_ / (*N*_*NW*_ +*N*_*NR*_ *+ N*_*NN*_), where *N*_*mn*_ is the number of transitions from state m to n (m, n ∈ {wake, NREM sleep, REM sleep}) in the observed period.

### Estimation of EEG delta power dynamics

The simulation and estimation of time constants for the daily increase and decrease of NREM-EEG delta power were carried out as described in the previous study [27]. In brief, Franken and colleagues’ method assumes that the dynamics of EEG delta power in each epoch (S) increases according to Eq (1) during awake and REM sleep phases and decreases according to Eq (2) during NREM sleep.


St+1=UA−UA−St⋅e−dtτi (1)

St+1=LA+St−LA⋅e−dtτd (2)

We simulated 72-h EEG recording composed of consecutive 8-s epochs (*dt* = 8 s). The percentage of delta power for each epoch is used for the calculation and estimation of each parameter. *UA* is the 99% level of the delta power percentage distribution in the NREM epochs, which is estimated by fitting a gaussian distribution to the histogram of the NREM delta power percentage. *LA* is defined as the intersection of the distributions of delta power percentage in NREM and REM epochs. *τ*_i_ and *τ*_d_ were estimated by comparing the simulated *S* and median delta power percentage of sustained (>5 min) NREM episodes. Best *τ*_i_ and *τ*_d_ were explored through the Bayesian optimization method implemented by using the GPyOpt python library (https://sheffieldml.github.io/GPyOpt/) with maximum iteration = 250 for each mouse data.

### Cage change experiment

For cage change experiment, AAV-administrated mice (9-week-old) were placed in the SSS chambers and habituated to the environment for 3 days. On the fourth day, the SSS chamber was replaced with a new one at ZT0. The sleep data of the fourth day were analyzed. The data of the first 3 days were used for baseline calculation.

### ES-mice production

Genetically modified mice were produced using the previously reported ES-mouse method, which allows us to analyze the behavior of F0 generation mice without crossing [74,75]. Mouse ES cells (ESCs) were established from blastocysts in 3i medium culture conditions as described previously [76]. Mouse strains used for the ESC establishment were as follows: *Cry1*^−/−^:*Cry2*^−/−^, *Cry1*^−/−^:*Cry2*^−/−^ mouse [35]; *Per1*^−/−^:*Per2*^−/−^, *Per1*^−/−^:*Per2*^−/−^ mouse [37]; *Vglut2*-*Cre*, heterozygous *Slc17a6*^*tm2(cre)Lowl*^/J mouse (The Jackson Laboratory, JAX stock #016963) [77].

Male ESCs were cultured as described previously [74,75]. Before cultivation, PURECoat amine dishes (Beckton-Dickinson, NJ, USA) was treated with a medium containing LIF plus 6-bromoindirubin-30-oxime (BIO) [78] for more than 5 h at 37 °C with 5% CO_2_. ESCs were seeded at 1 × 10^5^ cells per well and maintained at 37 °C in 5% CO_2_ under humidified conditions with a 3i culture medium (Y40010, Takara Bio, Japan) without feeder cells. The expanded ESCs were collected by adding 0.25% trypsin-EDTA solution and prepared as a cell suspension. Approximately 10 to 30 ESCs were injected into each ICR (CLEA Japan, Japan) 8-cell stage embryo, and the embryos were transferred into the uterus of pseudopregnant ICR female mice (SLC, Japan). We determined the contribution of the ESCs in an obtained ES-mouse by its coat color following a previously reported protocol [74,75]. The ES mice uncontaminated with ICR-derived cells were used for the experiment.

### AAV production

The protocol for AAV production was based on the previously reported protocol [79] with some modifications. AAV pro 293T (Takara Bio, Japan) was cultured in 150 mm dishes (Corning, USA) in a culture medium containing DMEM (high glucose) (Thermo Fisher Scientific, USA), 10% (v/v) FBS, and penicillin-streptomycin (Thermo Fisher Scientific, USA) at 37 °C in 5% CO_2_ under humidified conditions. pAAV, pUCmini-iCAP-PHPeB, and pHelper plasmid (Agilent, USA) were transfected into cells at 80% to 90% confluency using polyethyleneimine (Polysciences, USA). We employed a pAAV:pUCmini-iCAP-PHPeB:pHelper plasmid ratio of 1:4:2 based on micrograms of DNA (e.g., 5.7 μg of pAAV, 22.8 μg of pUCmini-iCAP-PHP, and 11.4 μg of pHelper). On the day following the transfection, the culture medium was replaced with 20 ml of a culture medium containing DMEM (high glucose, Glutamax) (Thermo Fisher Scientific, USA), 2% (v/v) FBS, MEM Non-Essential Amino Acids solution (NEAA) (Thermo Fisher Scientific, USA), and penicillin-streptomycin. On the third day following the transfection, the culture medium was collected and replaced with 20 ml of new culture medium containing DMEM (high glucose, Glutamax), 2% (v/v) FBS, MEM NEAA, and penicillin-streptomycin. The collected culture medium was stored at 4 °C. On the fifth day following the transfection, the cells and the culture medium were collected and combined with the stored medium. The suspension was separated into supernatant and cell pellet by centrifugation (2,000 × *g*, 20 min). From the supernatant, AAVs were concentrated by adding polyethylene glycol at a final concentration of 8% followed by centrifugation. From the cells, AAVs were extracted in a Tris-MgCl_2_ buffer (10 mM Tris (pH 8.0), 2 mM MgCl_2_) by repetitive freeze–thaw cycles. The obtained extract containing AAV was treated with Benzonase (100 U/ml) in a Tris-MgCl_2_ buffer, and then AAVs were purified by ultracentrifugation at 350,000 × *g* for 2 h 25 min (himac CP80WX and P70AT rotor, HITACHI, Japan) with Iodixanol density gradient solutions (15%, 25%, 40%, and 60% (wt/vol)). Viral particles were contained in a 40% solution, and this solution was ultrafiltered with an Amicon Ultra-15 device (100 kDa, Merck, Germany) to obtain the AAV stock solution for administration to mice.

To determine the AAV titer, virus solution was treated with Benzonase (50 U/ml, 37 °C, 1 h) followed by Proteinase K (0.25 mg/μL, 37 °C, 1 h). Subsequently, the viral genome was obtained by phenol-chloroform-isoamyl alcohol extraction followed by isopropanol precipitation. The AAV titer (vg/ml) was calculated by quantifying the number of WPRE sequences in the sample by qPCR using pAAV plasmid as a standard. The standard plasmids were prepared with PureYield Plasmid Midiprep System (Promega, USA) from SURE2 competent cells (Agilent, USA). The qPCR protocol was 60 s at 95 °C for preheating (initial denaturation) and 45 cycles from 10 s at 95 °C to 30 s at 60 °C using TB Green *Premix Ex Taq* GC (Takara Bio, Japan).

### Retro orbital injection of AAV to mice

Six-week-old male mice were anesthetized with 2% to 4% isoflurane and injected with 100 μL of AAV in their retro orbital sinus. S1 Table summarizes the AAVs used in this study and their administration conditions. The AAV-administrated mice were subjected to sleep phenotyping at 8-week-old.

### Estimation of transduction efficiency

Transduction efficiency was estimated based on previous reports [80,81]. After the sleep phenotyping, the brain hemisphere except for the olfactory bulb and cerebellum was collected from the AAV-administrated mouse. Brain DNA was purified using an Agencourt DNAdvance (Beckman Coulter, USA). The copy numbers of both the AAV vector genomes and mouse genomic DNA were quantified with a standard curve generated from known amounts of DNA. Vector genomes per cell were calculated by dividing the copy number of AAV vector genomes by diploid copies of the *Tbp* gene in the sample. The copy number of the AAV vector genomes and the *Tbp* gene were determined with WPRE-binding primers (5′-CTGTTGGGCACTGACAATTC-3′, 5′-GAAGGGACGTAGCAGAAGGA-3′) and Tbp-binding primers (5′-CCCCCTCTGCACTGAAATCA-3′; 5′-GTAGCAGCACAGAGCAAGCAA-3′) [70], respectively. The qPCR protocol was 60 s at 95 °C for preheating (initial denaturation) and 45 cycles from 10 s at 95 °C to 30 s at 60 °C using a TB Green *Premix Ex Taq* GC (Takara Bio, Japan).

### Statistics

No statistical method was used to predetermine the sample size. The sample sizes were determined based on previous experiences and reports. Experiments were repeated at least 2 times with the independent sets of the animals or independently prepared biochemical samples. The series of single/double-phosphomimetic screening was not repeated, but the mutants we focused on from the screening results were further analyzed in detail through additional independent experiments. In the sleep analysis, individuals with abnormal measurement signals or weakened individuals were excluded from the sleep data analyses because of their difficulties in accurate sleep phenotyping.

Statistical analyses were performed by Microsoft Excel and R version 3.5.2. Statistical tests were performed two-sided. For all figures, colors were selected using the khroma library (R version 3.5.2) considering color blindness. To compare 2 unpaired samples, the normality was tested using the Shapiro test at a significance level of 0.05. When the normality was not rejected in both groups, the homogeneity of variance was tested using the *F* test at a significance level of 0.05. When the null hypothesis of a normal distribution with equal variance for the 2 groups was not rejected, a Student *t* test was used. When the normality was not rejected but the null hypothesis of equal variance was rejected, a Welch *t* test was used. Otherwise, a 2-sample Wilcoxon test was applied.

To compare more than 2 samples against an identical sample, the normality was tested with the Kolmogorov–Smirnov test at a significance level of 0.05. When the normality was not rejected in all groups, the homogeneity of variance was tested with Bartlett test at a significance level of 0.05. When the null hypothesis of a normal distribution with equal variance was not rejected for all groups, Dunnett test was used. Otherwise, Steel test was applied.

For multiple comparisons between each group, the Tukey–Kramer test was used when the null hypothesis of a normal distribution with equal variance was not rejected for all groups. Otherwise, Steel–Dwass test was applied.

In this study, *p* < 0.05 was considered significant (*p < 0.05, **p < 0.01, ***p < 0.001, and n.s. for not significant). S1 Fig summarizes the workflow for selecting statistical method and the statistical analyses used in each experiment of this study and *p*-values.

## Supporting information

S1 Fig Workflow for selecting the statistical method. Workflow for selecting the statistical test methods used in this study. Based on the purpose of the comparison, normality and equality of variance were checked, and appropriate statistical method was selected. Details are provided in the Methods section.
(PDF)

S2 Fig Expression of the CaMKIIβ throughout the brain by AAV-PHP.eB. Volume-rendered and single-plane images of the brain expressing H2B-mCherry under *hSyn1* promoter by the AAV (mCherry, green) counterstained with RD2 (red). A volume-rendered image is shown in the center. Single-plane and magnified images are shown for cerebral cortex, thalamus, hippocampus, midbrain, cerebellum, striatum, and olfactory bulb. Scale bar in the center image, 3 mm; other scale bars, 100 μm. AAV, adeno-associated virus; CaMKIIβ, calmodulin-dependent protein kinase IIβ; *hSyn1*, human *synapsin-1*.
(TIFF)

S3 Fig Robust sleep induction by CaMKIIβ T287D mutant. **(A)** Expression levels of endogenous CaMKIIβ and AAV-mediated transduced CaMKIIβ in the brain. *Camk2b*^FLAG/FLAG^ represents homo knock-in mice in which the FLAG tag was inserted into the endogenous *Camk2b* locus. PBS: PBS-administrated mice. Immunoblotting against FLAG-tagged protein indicates that AAV-mediated expression of CaMKIIβ is lower than the expression level of endogenous CaMKIIβ. **(B)** Calculated transduction efficiency plotted against sleep duration. Transduction efficiency is an estimation of the number of AAV vector genomes present per cell in a mouse brain. After the SSS measurements, we purified the AAV vector genomes from the mice brains and then quantified them with a WPRE-specific primer set and normalized to mouse genomes. **(C)** Sleep transition profiles of mice expressing CaMKIIβ T287-related mutants shown in Fig 1F. The shaded areas represent SEM. **(D)** Sleep parameters during light or dark period of mice expressing CaMKIIβ T287-related mutants shown in Fig 1F. Multiple comparison tests were performed between all individual groups in each phase. **(E, F)** Sleep/wake parameters of mice expressing S114-related CaMKIIβ mutants **(C)** and S109-related CaMKIIβ mutants **(D)**, averaged over 6 days. The shaded areas represent SEM. Multiple comparison tests were performed between all individual groups and resulted in no significant differences. The underlying numerical data can be found in S1 Data, and uncropped or raw image files for S3A Fig are provided in S2 and S3 Data files. Error bars: SEM, *p < 0.05, **p < 0.01, ***p < 0.001, n.s.: no significance. AAV, adeno-associated virus; CaMKIIβ, calmodulin-dependent protein kinase IIβ; SSS, snappy sleep stager.
(PDF)

S4 Fig Characterization of CaMKIIβ T287D mutant based on EEG/EMG measurements. **(A)** Sleep parameters of mice expressing CaMKIIβ WT or the T287 mutant shown in Fig 1G–1I during light or dark periods**. (B, C)** EEG power spectra **(B)** and NREM power density in typical frequency domains **(C)** of mice expressing CaMKIIβ WT or the T287D mutant under the *hSyn1* promoter. **(D)** Estimated time constants for the increase of EEG delta power during awake/REM periods (τ_i_) and the decrease of NREM EEG delta power during the NREM period (τ_d_). The values are shown as mean ± SEM. CaMKIIβ WT or T287D were expressed under *hSynI* promoter. Other sleep parameters were shown in Fig 1G–1I. **(E, F)** Sleep profiles **(E)** and total sleep duration from ZT0 to ZT2 **(F)** of mice expressing WT CaMKIIβ (WT, *n* = 6) and the CaMKIIβ T287D mutants (T287D, *n* = 4) after cage change at ZT0. PBS: PBS-injected control mice (*n* = 6). “Basal” represents the sleep duration from ZT0 to ZT2 averaged over 3 days before the day of the cage change. The underlying data can be found in S1 Data. Error bars: SEM, *p < 0.05, **p < 0.01, ***p < 0.001, n.s.: no significance. CaMKIIβ, calmodulin-dependent protein kinase IIβ; EEG, electroencephalogram; EMG, electromyogram; *hSyn1*, human *synapsin-1*; NREM, nonrapid eye movement; REM, rapid eye movement; WT, wild-type; ZT, zeitgeber time.
(PDF)

S5 Fig CaMKIIβ T287 phosphorylation level increases upon sleep deprivation. **(A)** Sleep deprivation and peptide quantification procedures. The brains of the sleep-deprived and control mice were collected for MS-based peptide quantification. **(B)** Total CaMKIIβ and T287-phosphorylated peptides from brains of sleep-deprived and control mice, analyzed by SRM quantitative mass spectrometry. **(C)** Total CaMKIIα and T286-phosphorylated peptides from brains of sleep-deprived and control mice, analyzed by SRM quantitative mass spectrometry. Error bars: SEM. **(D)** Correlation of phosphorylation of CaMKIIα T286 and CaMKIIβ T287 in each brain. Each point corresponds to the quantification value obtained from individual mouse brain. The underlying data can be found in S1 Data. Error bars: SEM. See also S2 Table. CaMKII, calmodulin-dependent protein kinase II; MS, mass spectrometry; SRM, selected reaction monitoring.
(PDF)

S6 Fig Time-of-day analyses for sleep parameters of mice with perturbed CaMKII activity. **(A)** Sleep transition profiles of mice expressing the CaMKIIβ del mutant under *hSyn1* promoter shown in Fig 2B and 2C. The shaded areas represent SEM. **(B)** Sleep parameters of mice expressing the CaMKIIβ del mutants shown in Fig 2B and 2C during light or dark period. Multiple comparison tests were performed between all individual groups in each phase. **(C)** Sleep transition profiles of mice expressing AIP2 or RARA mutant under *hSyn1* promoter shown in Fig 2E and 2F. The shaded areas represent SEM. PBS: PBS-injected mice (*n* = 6). **(D)** Sleep parameters of mice expressing AIP2 or RARA mutant shown in Fig 2E and 2F during light or dark period. Multiple comparison tests were performed between all individual groups in each phase. The underlying data can be found in S1 Data. Error bars: SEM, *p < 0.05, **p < 0.01, ***p < 0.001, n.s.: no significance. AIP2, autocamtide inhibitory peptide 2; CaMKII, calmodulin-dependent protein kinase II; del, deletion; *hSyn1*, human *synapsin-1*;
(PDF)

S7 Fig EEG/EMG measurement-based analyses of the effects of CaMKII inhibitors on sleep phenotypes. **(A)** Sleep parameters of mice expressing AIP2 or the RARA mutant shown in Fig 2G–2I during light or dark periods**. (B, C)** EEG power spectra **(B)** and NREM power density in delta and theta domains **(C)** of mice expressing AIP2 or the RARA mutant. **(D)** Power densities in slow domains of mice expressing AIP2 or the RARA mutant. The underlying data can be found in S1 Data. Error bars: SEM, *p < 0.05, **p < 0.01, ***p < 0.001, n.s.: no significance. AIP2, autocamtide inhibitory peptide 2; CaMKII, calmodulin-dependent protein kinase II; EEG, electroencephalogram; EMG, electromyogram; NREM, nonrapid eye movement.
(PDF)

S8 Fig Sleep phenotypes of *Camk2b* KO mice. **(A)** The genotyping of *Camk2b* KO mice. The relative amount of intact DNA for each target sequence was normalized to be 100% for WT mouse. The qPCR was performed with 2 independent primer pairs #1 and #2 for the 3 target sites. When the 0.5% criteria were met in either set, the mouse was considered a KO mouse. The qPCR results indicate that triple gRNA sequences used for this KO effectively induced deletion mutations in the chromosomal target-1 and target-2 regions covered by the alternative primer pair #2. The persistent presence of target-3 signals amplified by both primer pairs #1 and #2 suggests that the chromosomal target-3 region was not effectively deleted by the gRNA used in this experiment. All the mice (*n* = 5) were confirmed as KO mice by the qPCR with primer pair #2. **(B-D)** Sleep parameters **(B and D)** and sleep profiles **(C)** measured by EEG/EMG recordings for *Camk2b* KO mice and WT C57BL/6N mice. **(E, F)** Normalized EEG power spectra **(E)** and NREM power density in typical frequency domains **(F)** of *Camk2b* KO mice and WT C57BL/6N mice. EEG power was normalized relative to the total power in each frequency band. **(G, H)** EEG power spectra **(G)** and NREM power density in typical frequency domains **(H)** of *Camk2b* KO mice and WT C57BL/6N mice without normalization. The underlying data can be found in S1 Data. Error bars: SEM, *p < 0.05, **p < 0.01, ***p < 0.001, n.s.: no significance. EEG, electroencephalogram; EMG, electromyogram; KO, knockout; NREM, nonrapid eye movement; WT, wild-type.
(PDF)

S9 Fig Phosphorylation of CaMKIIβ regulates sleep induction and sleep needs. **(A-B)** EEG power spectra **(A)** and NREM power density in typical frequency domains **(B)** of mice expressing WT CaMKIIβ or the T287D mutant under the *Camk2a* promoter. **(C)** Estimated time constants for the increase of EEG delta power during awake/REM periods (τ_i_) and the decrease of NREM EEG delta power during the NREM period (τ_d_). The values are shown as mean ± SEM. CaMKIIβ WT or T287D were expressed under *Camk2a* promoter. Other sleep parameters were shown in Fig 3C–3E. **(D)** Genomic locus obtained as *Camk2b* promoter sequence, mapped onto GRCm38/mm10 using UCSC genome Browser (http://genome.ucsc.edu/) [82]. **(E, F)** Sleep/wake parameters **(E)** and sleep profiles **(F)** measured by SSS, averaged over 6 days, for mice expressing WT CaMKIIβ or the T287D mutant (T287D) under the *Camk2b* promoter. The underlying data can be found in S1 Data. Error bars: SEM, *p < 0.05, **p < 0.01, ***p < 0.001, n.s.: no significance. CaMKIIβ, calmodulin-dependent protein kinase IIβ; EEG, electroencephalogram; NREM, nonrapid eye movement; SSS, snappy sleep stager; WT, wild-type.
(PDF)

S10 Fig Sleep maintenance activity elicited by T287D:T306D:T307D CaMKIIβ mutant. **(A)** Calculated transduction efficiency plotted against sleep duration. The calculation was conducted same as S3 Fig. **(B)** Sleep duration and correlation diagram of daily *P*_*WS*_ and *P*_*SW*_ of mice expressing double-phopshomimetic mutants with sleep maintenance activity. Measurements are independent from those in Fig 5A. For the comparisons of sleep duration, multiple testing was performed against WT CaMKIIβ-expressing mice. In the *P*_*WS*_-*P*_*SW*_ diagram, residues marked in red indicate mutants with low lower *P*_*WS*_ and *P*_*SW*_ (i.e., higher sleep maintenance activity). **(C, D)** Profiles of sleep and transition probability, averaged over 6 days, of mice expressing the double/triple CaMKIIβ mutants shown in S8B (C) Fig and Fig 5C and 5D. **(E)** Sleep episode duration, averaged over 6 days, of mice expressing CaMKIIβ mutants with D or A substitutions of sleep-stabilizing residues shown in Fig 5C. Comparisons were performed between the 2 corresponding mice groups. **(F)** Sleep parameters during light or dark periods, averaged over 6 days, of mice expressing the CaMKIIβ mutants with D or A substitutions of sleep-stabilizing residues shown in Fig 5C. Comparisons were performed between the 2 corresponding mice groups. The underlying data can be found in S1 Data. Error bars: SEM, *p < 0.05, **p < 0.01, ***p < 0.001, n.s.: no significance. CaMKIIβ, calmodulin-dependent protein kinase IIβ; WT, wild-type.
(PDF)

S11 Fig Characterization of the CaMKIIβ T287D:T306D:T306D mutant based on EEG/EMG measurements. **(A)** Sleep parameters of mice expressing CaMKIIβ WT or the T287D:T306D:T307D mutant shown in Fig 5D–5G during light or dark periods. **(B, C)** EEG power spectra **(B)** and NREM power density in typical frequency domains **(C)** of mice expressing WT CaMKIIβ and the T287D:T306D:T307D mutant shown in Fig 5D–5G. The underlying data can be found in S1 Data. Error bars: SEM, *p < 0.05, **p < 0.01, ***p < 0.001, n.s.: no significance. CaMKIIβ, calmodulin-dependent protein kinase IIβ; EEG, electroencephalogram; EMG, electromyogram; NREM, nonrapid eye movement; WT, wild-type.
(PDF)

S12 Fig Multisite phosphorylation of CaMKIIβ underlies multistep sleep regulation. **(A)** Sleep/wake parameters of mice expressing the quadruple-phosphomimetic CaMKIIβ mutants related to T311. Multiple comparison tests were performed between all individual groups. Error bars: SEM, *p < 0.05, **p < 0.01, ***p < 0.001. **(B)** Expression levels of each mutant in the 293T analyzed by the dot blotting of cell lysates. The values are shown in the mean ± SD (*n* = 2, independent experiments). **(C)** In vitro kinase activity of CaMKIIβ phosphomimetic mutants. Phosphorylation (%) indicates the percentage of the phosphorylated substrate relative to the total peptide in the presence or absence of CaM. The represented values are the mean ± SD (*n* = 2, independent experiments). The relative amount of CaMKIIβ in the cell lysates was adjusted to be the same level as WT. The mutants with blue labels exhibited <25% lower expression than the WT in both trials (see black and orange dashed lines in panel **B**). The mutants with green labels exhibited <25% lower expression than the WT in one of the 2 trials. When the expression level is <25% compared to WT, the relative amount of the CaMKIIβ mutant in the kinase assay was lower than that of the WT. **(D)** Time series changes of sleep-controlling residues phosphorylation under different Ca^2+^ conditions in vitro. The represented values are the mean ± SD (*n* = 2 independent experiments). The signal intensity of the detected peptides was normalized to the maximum value in the time series. The quantified values at 0 min were obtained from the sample before adding CaM and were shared in every Ca^2+^ conditions. The dashed lines trace the dynamics of T287 phosphorylation in the 0.5 mM Ca^2+^ condition. **(E)** Example chromatogram of SRM measurement for pS182 peptide. The red line indicates the retention time of a peptide phosphorylated at S182. The blue line indicates the retention time of a peptide phosphorylated at T177. **(F)** S26-, T306-, T307-, or T311-phosphorylated peptides from 293T cell lysates. The 293T cells were transfected with plasmids overexpressing CaMKIIβ WT, T287D, or K43R:T287D. Each peptide was quantified by SRM methods and normalized to the level of total CaMKIIβ, which was also quantified by SRM methods for analyzing the CaMKIIβ-derived unphosphorylated peptides. The values are shown as mean ± SD. The underlying data can be found in S1 Data. Quantified values of CaMKIIβ peptides for S12D Fig are provided in S4 Data file. See also S3 Table. CaM, calmodulin; CaMKIIβ, calmodulin-dependent protein kinase IIβ; SRM, selected reaction monitoring; WT, wild-type.
(PDF)

S13 Fig CaMKIIβ S26/CaMKIIα S25 is phosphorylated in vivo. Phosphorylation of S26 (CaMKIIβ) or S25 (CaMKIIα) residue in mice brain. Mice brain samples shown in S5 Fig were also subjected to SRM analysis with mass spectrometry method for analyzing the phosphorylation of S26 (CaMKIIβ) or S25 (CaMKIIα) residues. Representative chromatograms shown in left indicated that a synthesized and heavy-labeled phosphorylated peptide, of which sequence is identical to a trypsin-digested peptide sequence corresponding to S26 (CaMKIIβ) or S25 (CaMKIIα) was detected at retention time approximately 20 min. Medium-labeled peptide sample (derived from internal control mixture) and light-labeled peptide sample (derived from individual samples) also showed a peak at retention time approximately 20 min. The product ion spectrum on the right shows that each product ion from the 5 different transitions in the 3 samples has a similar intensity distribution. These results suggest that a peptide corresponding to phosphorylated S26 (CaMKIIβ) or S25 (CaMKIIα) was included in trypsin-digested mice brain samples. CaMKIIβ, calmodulin-dependent protein kinase IIβ; SRM, selected reaction monitoring.
(PDF)

S1 Table Summary of AAV applications and conditions. (XLSX)

S2 Table SRM transition list for the quantification of CaMKII-derived peptides from brain samples. (XLSX)

S3 Table SRM transition list for the quantification of CaMKII-derived peptides from in vitro samples. (XLSX)

S1 Data Numerical data and statistical analyses. (XLSX)

S2 Data Uncropped image of western blotting data; related to S3A Fig. (PDF)

S3 Data Raw image files of western blotting data; related to S3A Fig. (ZIP)

S4 Data Quantified values of peptides derived from purified CaMKIIβ; related to S12D Fig. (XLSX)

## Abbreviations

AAV: adeno-associated virus
AIP2: autocamtide inhibitory peptide 2
CaM: calmodulin
CaMKII: calmodulin-dependent protein kinase II
DD: constant dark
del: deletion
DIO: double-floxed inverted open reading frame
DKO: double knockout
DMEM: Dulbecco’s Modified Eagle Medium
DTE: dendritic targeting element
EEG: electroencephalogram
EMG: electromyogram
ESC: ES cell
FFT: fast Fourier transformation
HPLC: high-performance liquid chromatography
hSyn1: human *synapsin-1*
LD: light/dark
NREM: nonrapid eye movement
PTS: phase-transfer surfactant
SRM: selected reaction monitoring
SSS: snappy sleep stager
WT: wild-type
