# Supplementary material for: Distinct phosphorylation states of mammalian CaMKIIβ control the induction and maintenance of sleep
Source: PLoS Biol. 2022 Oct 4;20(10):e3001813. doi: 10.1371/journal.pbio.3001813 (PMC9531794; doi:10.1371/journal.pbio.3001813)
Supplement: S1 Fig — Workflow for selecting the statistical test methods used in this study. Based on the purpose of the comparison, normality and equality of variance were checked, and appropriate statistical method was selected. Details are provided in the Methods section. (PDF) [file pbio.3001813.s001.pdf]

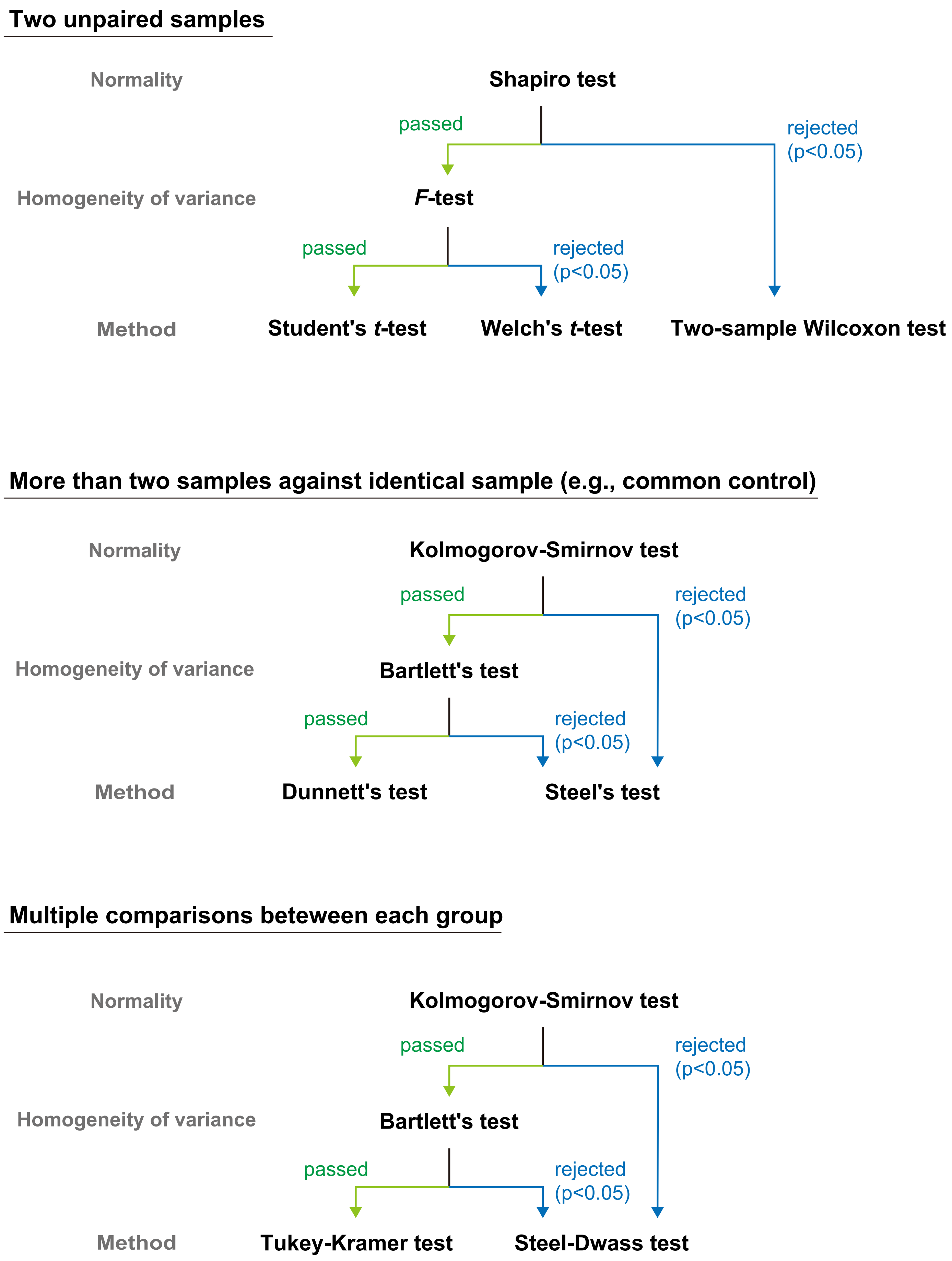
Two unpaired samples
Normality
Shapiro test
passed
rejected (p<0.05)
Homogeneity of variance
F-test
passed
rejected (p<0.05)
Method
Student's t-test
Welch's t-test
Two-sample Wilcoxon test
More than two samples against identical sample (e.g., common control)
Normality
Kolmogorov-Smirnov test
passed
rejected (p<0.05)
Homogeneity of variance
Bartlett's test
passed
rejected (p<0.05)
Method
Dunnett's test
Steel's test
Multiple comparisons beteween each group
Normality
Kolmogorov-Smirnov test
passed
rejected (p<0.05)
Homogeneity of variance
Bartlett's test
passed
rejected (p<0.05)
Method
Tukey-Kramer test
Steel-Dwass test

Figure S1
