# Supplementary figures and images for: Distinct phosphorylation states of mammalian CaMKIIβ control the induction and maintenance of sleep
Source: PLoS Biol. 2022 Oct 4;20(10):e3001813. doi: 10.1371/journal.pbio.3001813 (PMC9531794; doi:10.1371/journal.pbio.3001813)

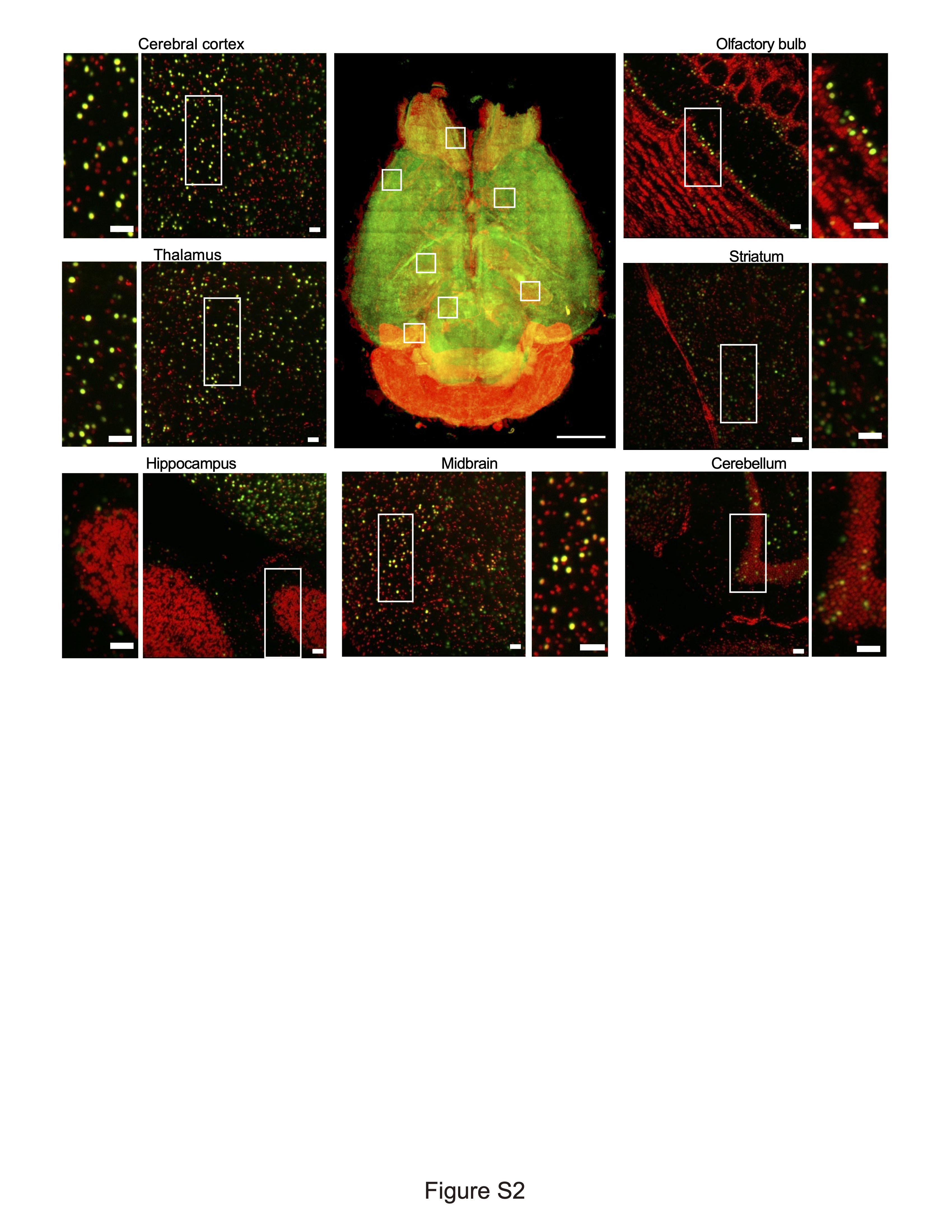

Supplement: S2 Fig — Volume-rendered and single-plane images of the brain expressing H2B-mCherry under hSyn1 promoter by the AAV (mCherry, green) counterstained with RD2 (red). A volume-rendered image is shown in the center. Single-plane and magnified images are shown for cerebral cortex, thalamus, hippocampus, midbrain, cerebellum, striatum, and olfactory bulb. Scale bar in the center image, 3 mm; other scale bars, 100 μm. AAV, adeno-associated virus; CaMKIIβ, calmodulin-dependent protein kinase IIβ; hSyn1, human synapsin-1. (TIFF) [file pbio.3001813.s002.tiff]

**A**

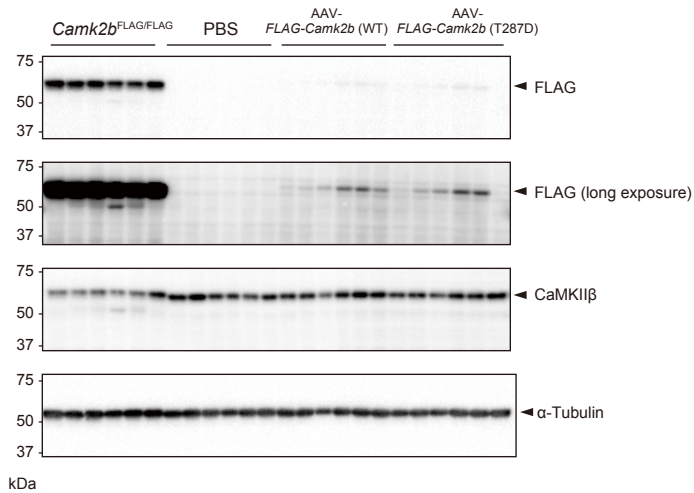

**C**

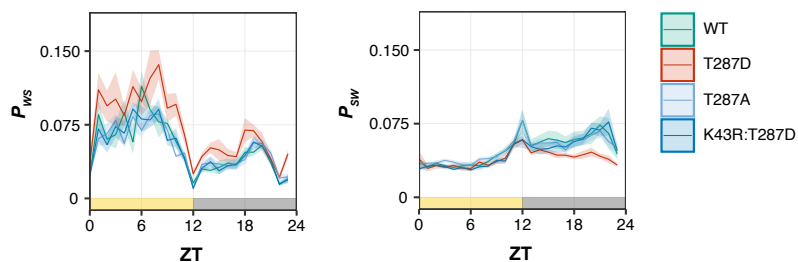

D

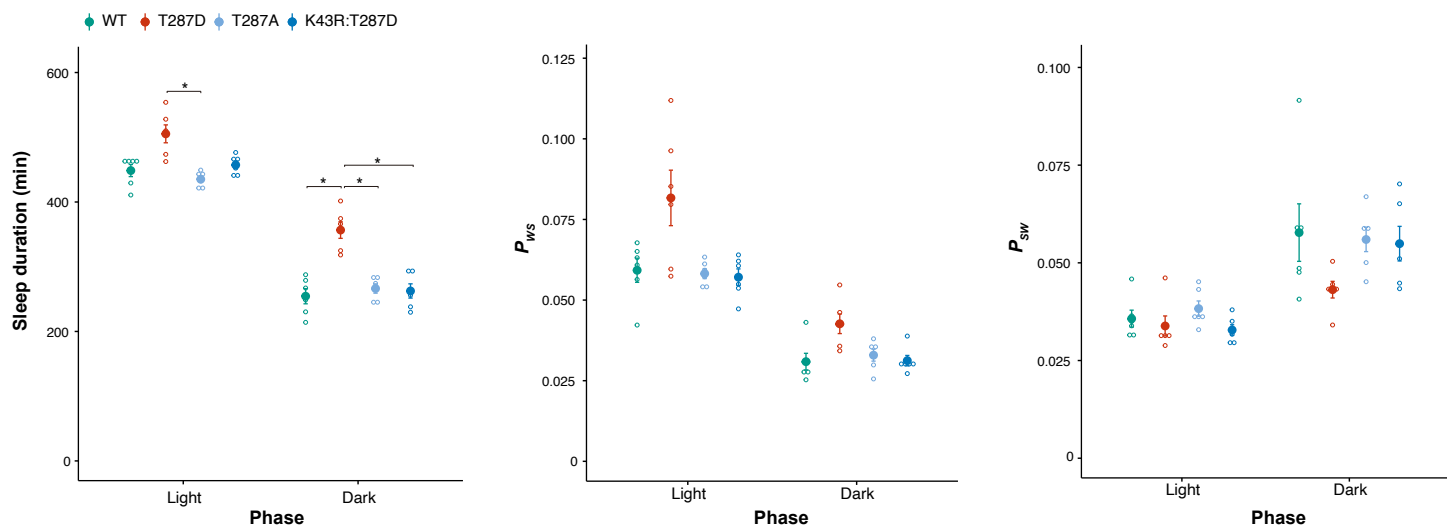

# E

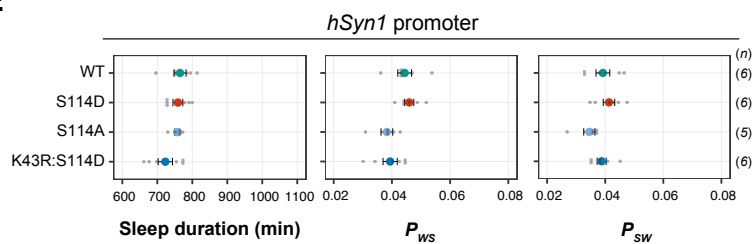

# B

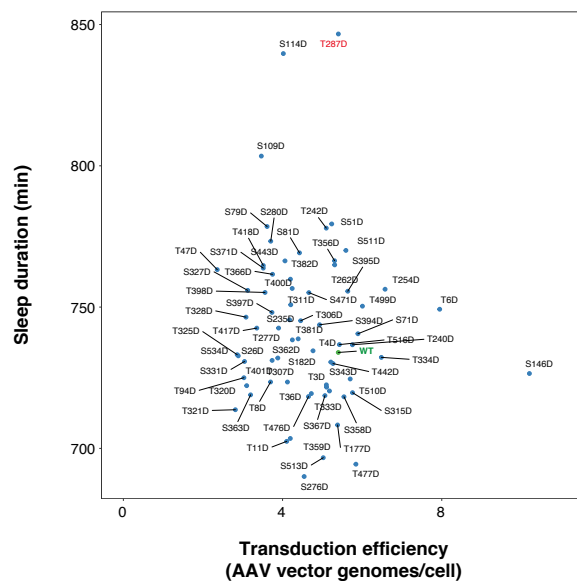**F**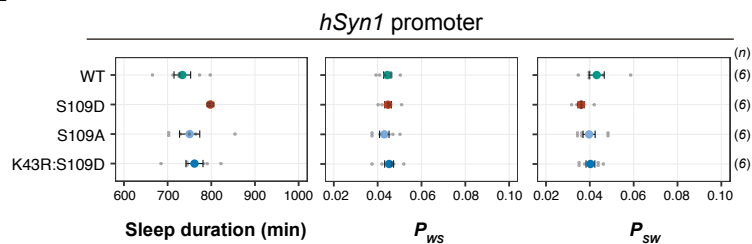

Figure S3

Supplement: S3 Fig — (A) Expression levels of endogenous CaMKIIβ and AAV-mediated transduced CaMKIIβ in the brain. Camk2bFLAG/FLAG represents homo knock-in mice in which the FLAG tag was inserted into the endogenous Camk2b locus. PBS: PBS-administrated mice. Immunoblotting against FLAG-tagged protein indicates that AAV-mediated expression of CaMKIIβ is lower than the expression level of endogenous CaMKIIβ. (B) Calculated transduction efficiency plotted against sleep duration. Transduction efficiency is an estimation of the number of AAV vector genomes present per cell in a mouse brain. After the SSS measurements, we purified the AAV vector genomes from the mice brains and then quantified them with a WPRE-specific primer set and normalized to mouse genomes. (C) Sleep transition profiles of mice expressing CaMKIIβ T287-related mutants shown in Fig 1F. The shaded areas represent SEM. (D) Sleep parameters during light or dark period of mice expressing CaMKIIβ T287-related mutants shown in Fig 1F. Multiple comparison tests were performed between all individual groups in each phase. (E, F) Sleep/wake parameters of mice expressing S114-related CaMKIIβ mutants (C) and S109-related CaMKIIβ mutants (D), averaged over 6 days. The shaded areas represent SEM. Multiple comparison tests were performed between all individual groups and resulted in no significant differences. The underlying numerical data can be found in S1 Data, and uncropped or raw image files for S3A Fig are provided in S2 and S3 Data files. Error bars: SEM, *p < 0.05, **p < 0.01, ***p < 0.001, n.s.: no significance. AAV, adeno-associated virus; CaMKIIβ, calmodulin-dependent protein kinase IIβ; SSS, snappy sleep stager. (PDF) [file pbio.3001813.s003.pdf]

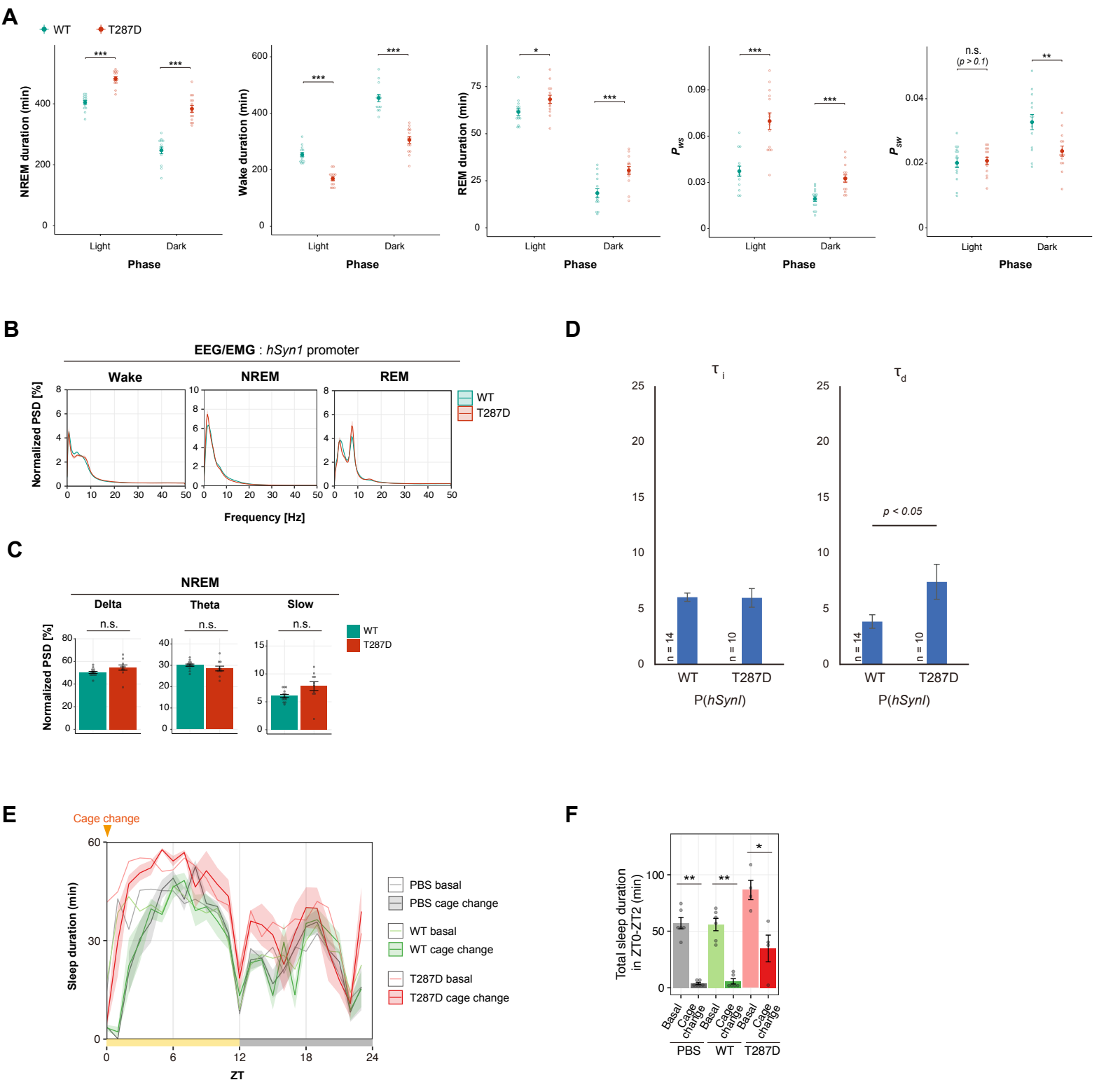

Figure S4

Supplement: S4 Fig — (A) Sleep parameters of mice expressing CaMKIIβ WT or the T287 mutant shown in Fig 1G–1I during light or dark periods. (B, C) EEG power spectra (B) and NREM power density in typical frequency domains (C) of mice expressing CaMKIIβ WT or the T287D mutant under the hSyn1 promoter. (D) Estimated time constants for the increase of EEG delta power during awake/REM periods (τi) and the decrease of NREM EEG delta power during the NREM period (τd). The values are shown as mean ± SEM. CaMKIIβ WT or T287D were expressed under hSynI promoter. Other sleep parameters were shown in Fig 1G–1I. (E, F) Sleep profiles (E) and total sleep duration from ZT0 to ZT2 (F) of mice expressing WT CaMKIIβ (WT, n = 6) and the CaMKIIβ T287D mutants (T287D, n = 4) after cage change at ZT0. PBS: PBS-injected control mice (n = 6). “Basal” represents the sleep duration from ZT0 to ZT2 averaged over 3 days before the day of the cage change. The underlying data can be found in S1 Data. Error bars: SEM, *p < 0.05, **p < 0.01, ***p < 0.001, n.s.: no significance. CaMKIIβ, calmodulin-dependent protein kinase IIβ; EEG, electroencephalogram; EMG, electromyogram; hSyn1, human synapsin-1; NREM, nonrapid eye movement; REM, rapid eye movement; WT, wild-type; ZT, zeitgeber time. (PDF) [file pbio.3001813.s004.pdf]

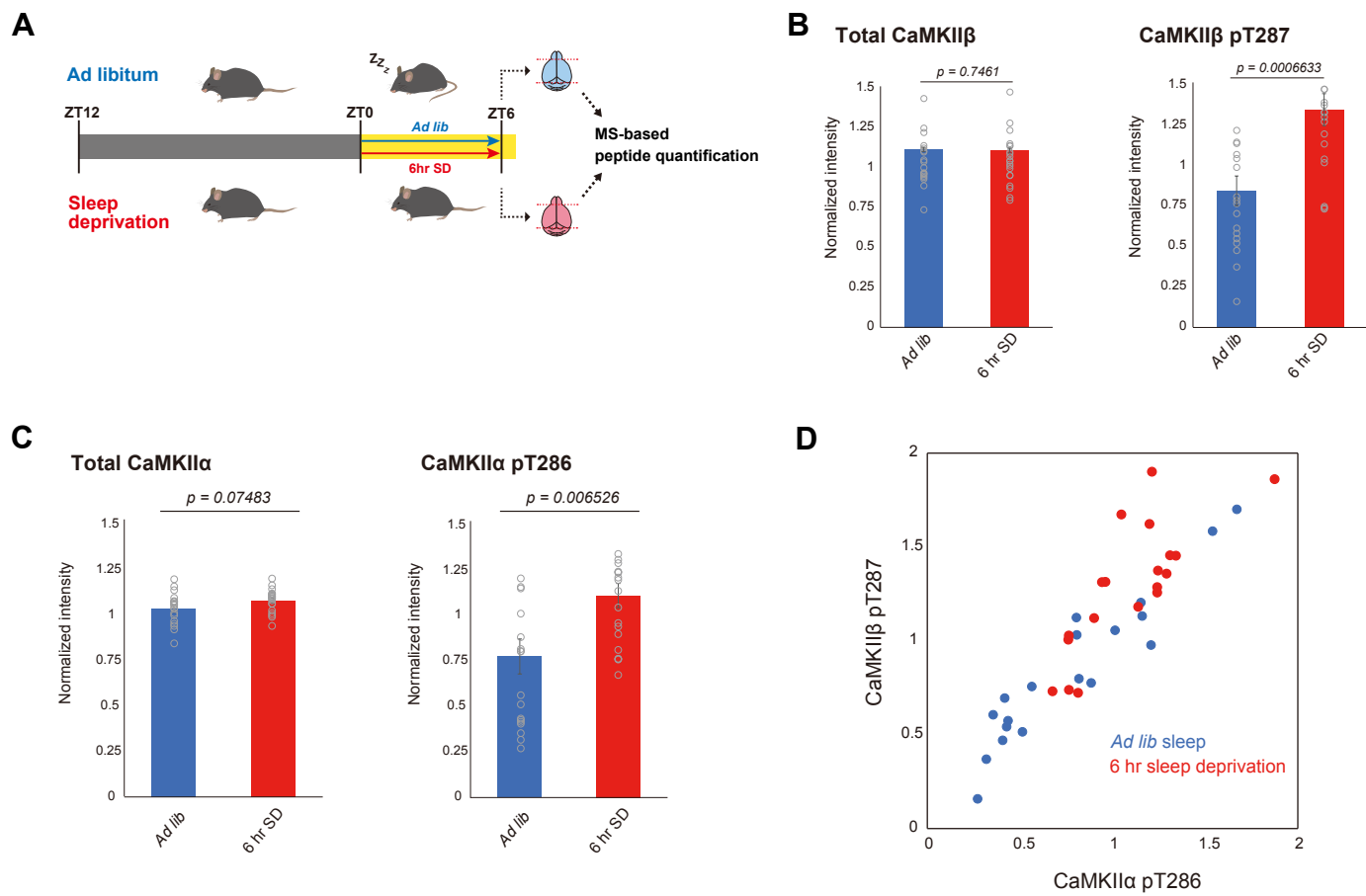

Figure S5

Supplement: S5 Fig — (A) Sleep deprivation and peptide quantification procedures. The brains of the sleep-deprived and control mice were collected for MS-based peptide quantification. (B) Total CaMKIIβ and T287-phosphorylated peptides from brains of sleep-deprived and control mice, analyzed by SRM quantitative mass spectrometry. (C) Total CaMKIIα and T286-phosphorylated peptides from brains of sleep-deprived and control mice, analyzed by SRM quantitative mass spectrometry. Error bars: SEM. (D) Correlation of phosphorylation of CaMKIIα T286 and CaMKIIβ T287 in each brain. Each point corresponds to the quantification value obtained from individual mouse brain. The underlying data can be found in S1 Data. Error bars: SEM. See also S2 Table. CaMKII, calmodulin-dependent protein kinase II; MS, mass spectrometry; SRM, selected reaction monitoring. (PDF) [file pbio.3001813.s005.pdf]

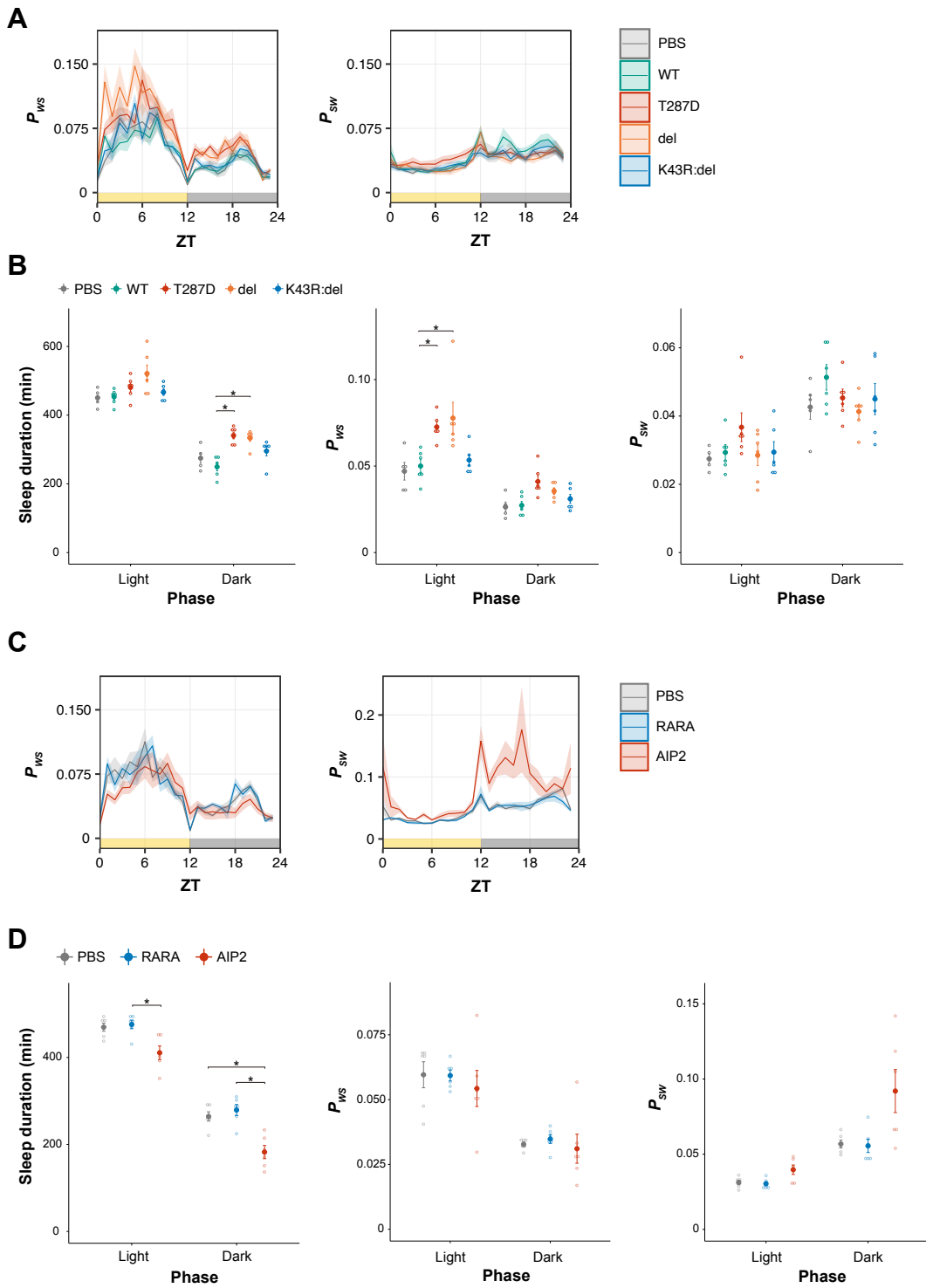

Figure S6

Supplement: S6 Fig — (A) Sleep transition profiles of mice expressing the CaMKIIβ del mutant under hSyn1 promoter shown in Fig 2B and 2C. The shaded areas represent SEM. (B) Sleep parameters of mice expressing the CaMKIIβ del mutants shown in Fig 2B and 2C during light or dark period. Multiple comparison tests were performed between all individual groups in each phase. (C) Sleep transition profiles of mice expressing AIP2 or RARA mutant under hSyn1 promoter shown in Fig 2E and 2F. The shaded areas represent SEM. PBS: PBS-injected mice (n = 6). (D) Sleep parameters of mice expressing AIP2 or RARA mutant shown in Fig 2E and 2F during light or dark period. Multiple comparison tests were performed between all individual groups in each phase. The underlying data can be found in S1 Data. Error bars: SEM, *p < 0.05, **p < 0.01, ***p < 0.001, n.s.: no significance. AIP2, autocamtide inhibitory peptide 2; CaMKII, calmodulin-dependent protein kinase II; del, deletion; hSyn1, human synapsin-1; (PDF) [file pbio.3001813.s006.pdf]

**A**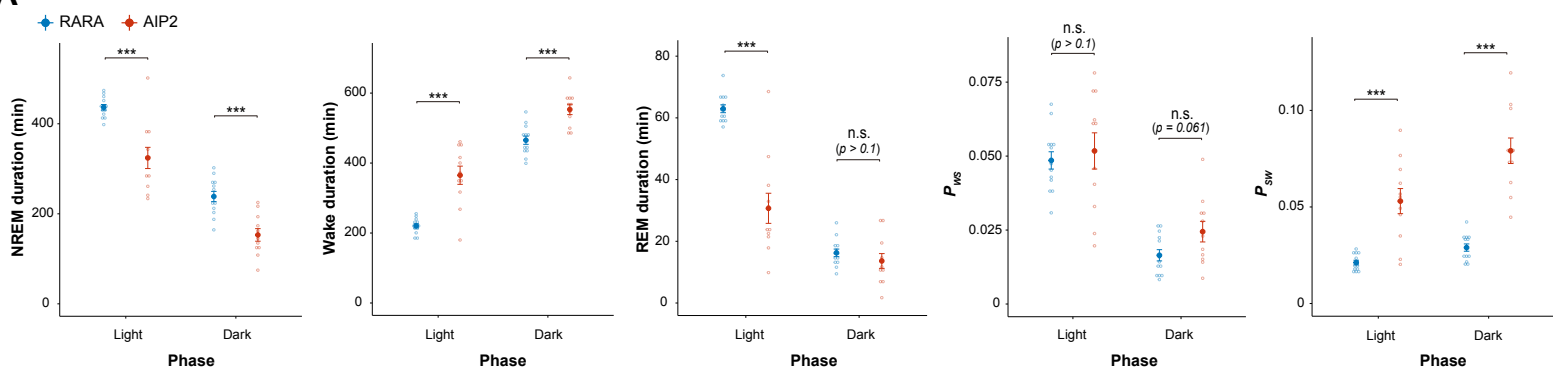**B**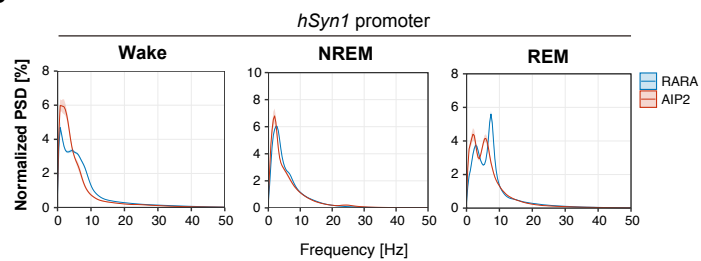**C**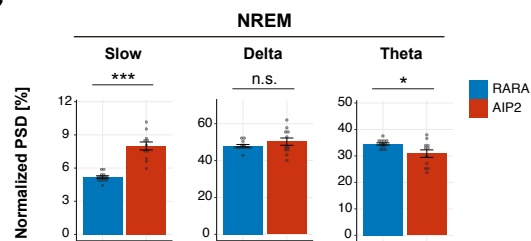**D**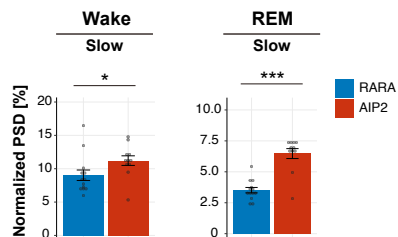

Figure S7

Supplement: S7 Fig — (A) Sleep parameters of mice expressing AIP2 or the RARA mutant shown in Fig 2G–2I during light or dark periods. (B, C) EEG power spectra (B) and NREM power density in delta and theta domains (C) of mice expressing AIP2 or the RARA mutant. (D) Power densities in slow domains of mice expressing AIP2 or the RARA mutant. The underlying data can be found in S1 Data. Error bars: SEM, *p < 0.05, **p < 0.01, ***p < 0.001, n.s.: no significance. AIP2, autocamtide inhibitory peptide 2; CaMKII, calmodulin-dependent protein kinase II; EEG, electroencephalogram; EMG, electromyogram; NREM, nonrapid eye movement. (PDF) [file pbio.3001813.s007.pdf]

A

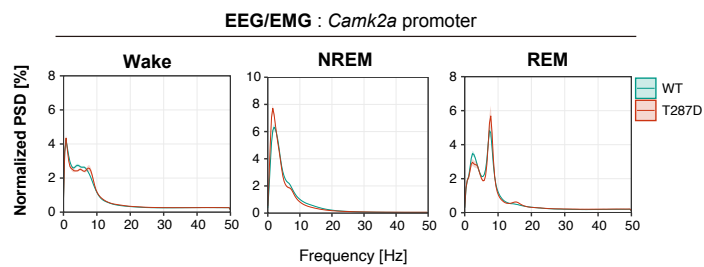

B

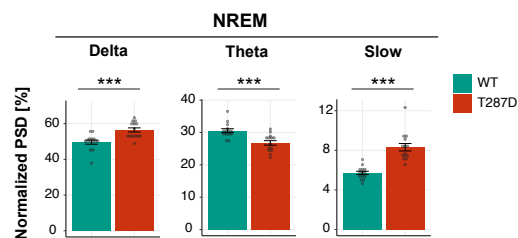

C

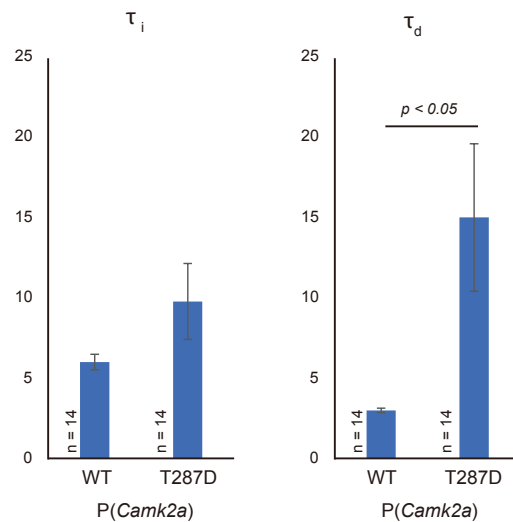

D

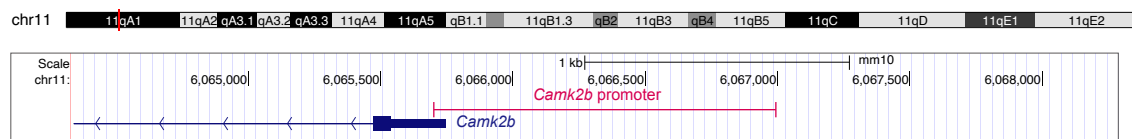

E

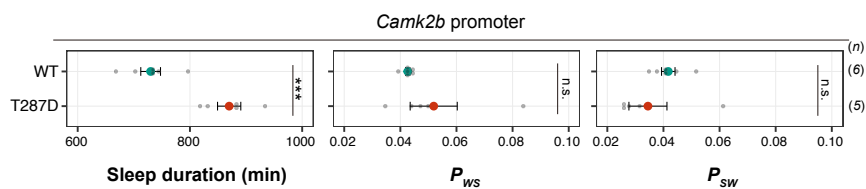

F

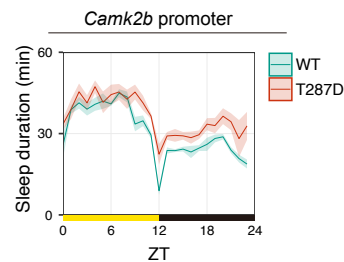

Figure S9

Supplement: S9 Fig — (A-B) EEG power spectra (A) and NREM power density in typical frequency domains (B) of mice expressing WT CaMKIIβ or the T287D mutant under the Camk2a promoter. (C) Estimated time constants for the increase of EEG delta power during awake/REM periods (τi) and the decrease of NREM EEG delta power during the NREM period (τd). The values are shown as mean ± SEM. CaMKIIβ WT or T287D were expressed under Camk2a promoter. Other sleep parameters were shown in Fig 3C–3E. (D) Genomic locus obtained as Camk2b promoter sequence, mapped onto GRCm38/mm10 using UCSC genome Browser (http://genome.ucsc.edu/) [82]. (E, F) Sleep/wake parameters (E) and sleep profiles (F) measured by SSS, averaged over 6 days, for mice expressing WT CaMKIIβ or the T287D mutant (T287D) under the Camk2b promoter. The underlying data can be found in S1 Data. Error bars: SEM, *p < 0.05, **p < 0.01, ***p < 0.001, n.s.: no significance. CaMKIIβ, calmodulin-dependent protein kinase IIβ; EEG, electroencephalogram; NREM, nonrapid eye movement; SSS, snappy sleep stager; WT, wild-type. (PDF) [file pbio.3001813.s009.pdf]

**A**

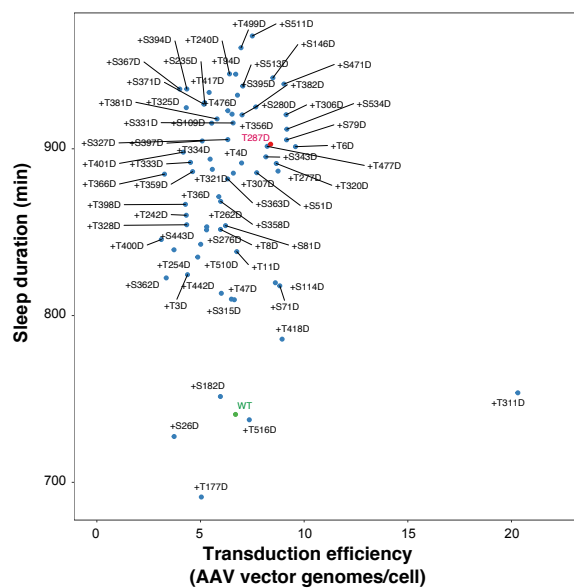

# B

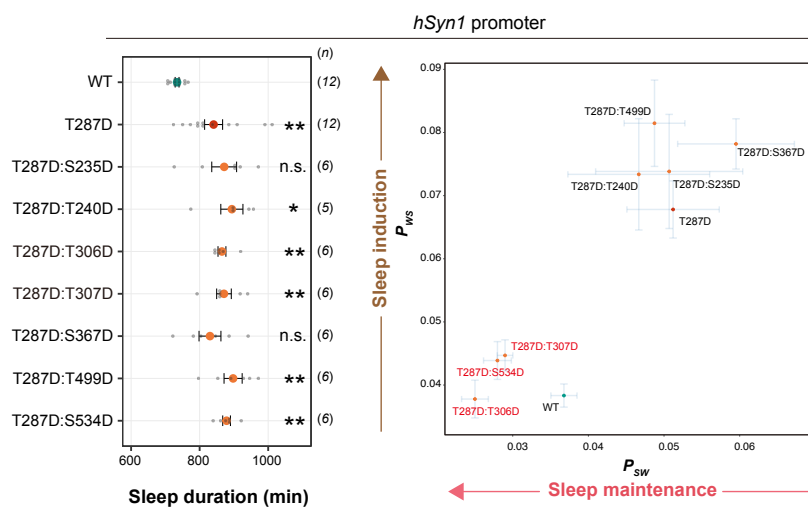

**C**

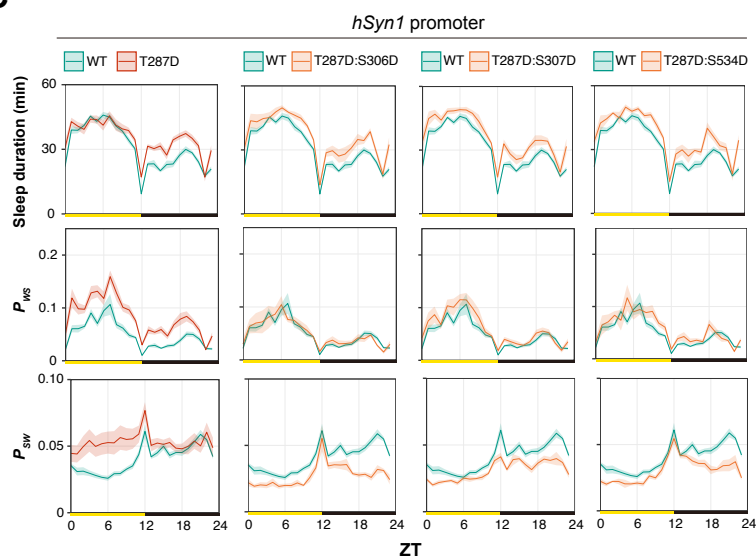

# D

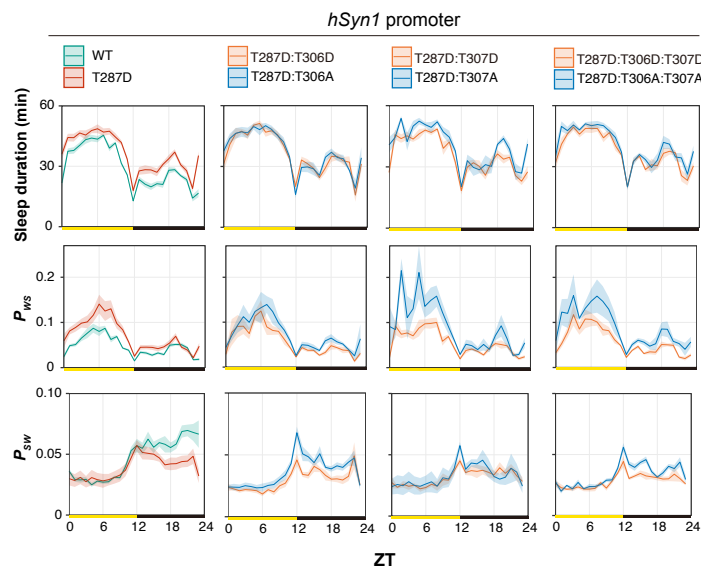

# E

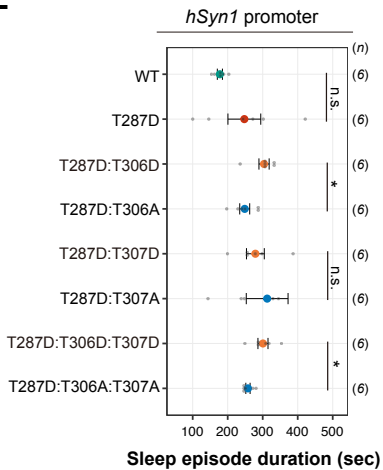

**F**

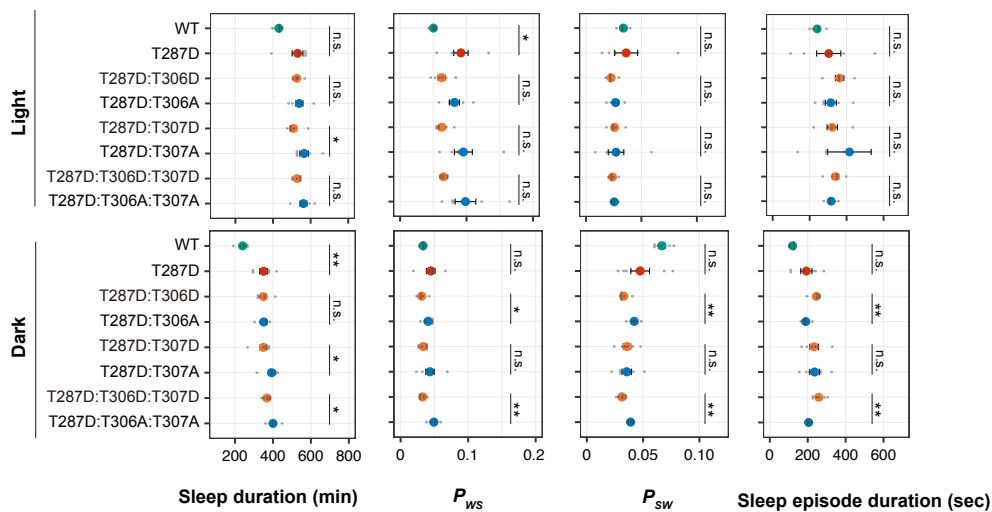

Figure S10

Supplement: S10 Fig — (A) Calculated transduction efficiency plotted against sleep duration. The calculation was conducted same as S3 Fig. (B) Sleep duration and correlation diagram of daily PWS and PSW of mice expressing double-phopshomimetic mutants with sleep maintenance activity. Measurements are independent from those in Fig 5A. For the comparisons of sleep duration, multiple testing was performed against WT CaMKIIβ-expressing mice. In the PWS-PSW diagram, residues marked in red indicate mutants with low lower PWS and PSW (i.e., higher sleep maintenance activity). (C, D) Profiles of sleep and transition probability, averaged over 6 days, of mice expressing the double/triple CaMKIIβ mutants shown in S8B (C) Fig and Fig 5C and 5D. (E) Sleep episode duration, averaged over 6 days, of mice expressing CaMKIIβ mutants with D or A substitutions of sleep-stabilizing residues shown in Fig 5C. Comparisons were performed between the 2 corresponding mice groups. (F) Sleep parameters during light or dark periods, averaged over 6 days, of mice expressing the CaMKIIβ mutants with D or A substitutions of sleep-stabilizing residues shown in Fig 5C. Comparisons were performed between the 2 corresponding mice groups. The underlying data can be found in S1 Data. Error bars: SEM, *p < 0.05, **p < 0.01, ***p < 0.001, n.s.: no significance. CaMKIIβ, calmodulin-dependent protein kinase IIβ; WT, wild-type. (PDF) [file pbio.3001813.s010.pdf]

**A**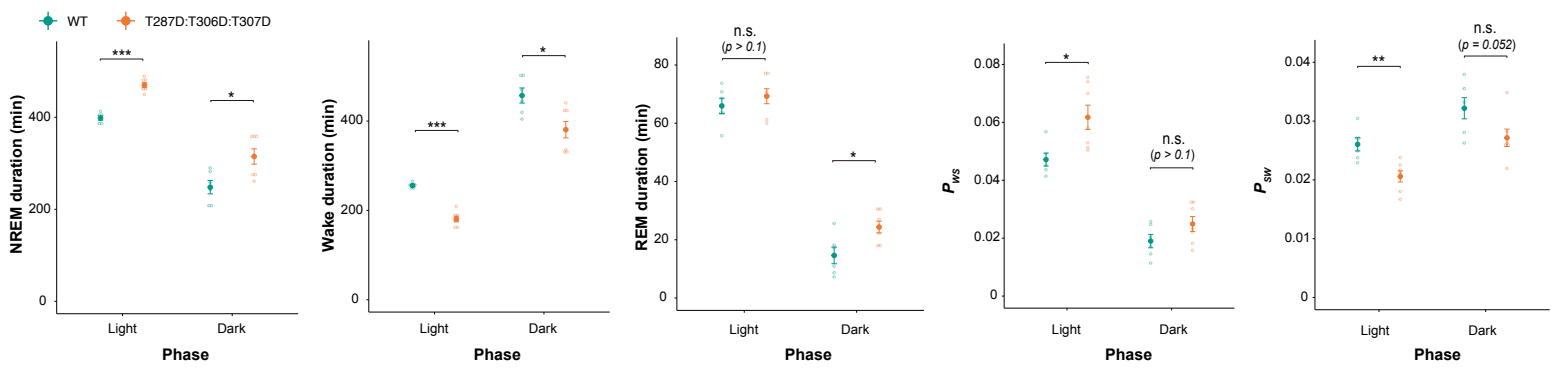**B**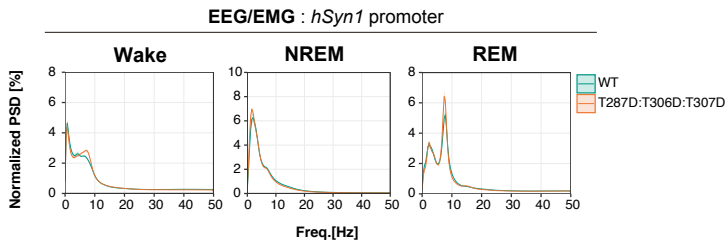**C**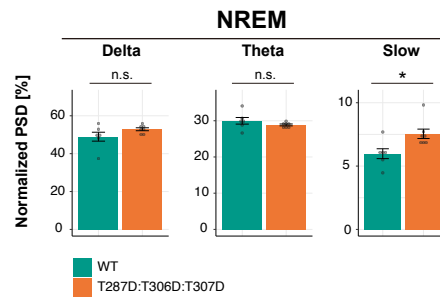

Figure S11

Supplement: S11 Fig — (A) Sleep parameters of mice expressing CaMKIIβ WT or the T287D:T306D:T307D mutant shown in Fig 5D–5G during light or dark periods. (B, C) EEG power spectra (B) and NREM power density in typical frequency domains (C) of mice expressing WT CaMKIIβ and the T287D:T306D:T307D mutant shown in Fig 5D–5G. The underlying data can be found in S1 Data. Error bars: SEM, *p < 0.05, **p < 0.01, ***p < 0.001, n.s.: no significance. CaMKIIβ, calmodulin-dependent protein kinase IIβ; EEG, electroencephalogram; EMG, electromyogram; NREM, nonrapid eye movement; WT, wild-type. (PDF) [file pbio.3001813.s011.pdf]

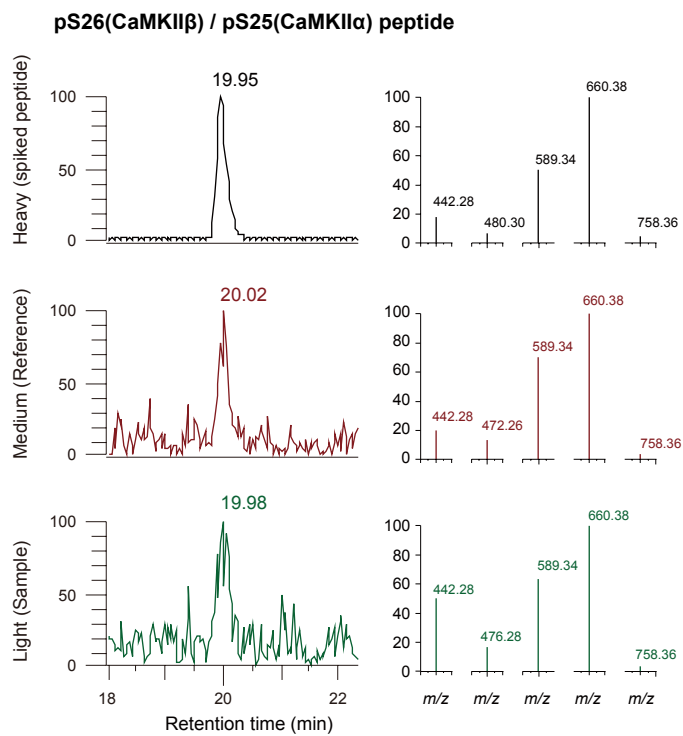

Figure S13

Supplement: S13 Fig — Phosphorylation of S26 (CaMKIIβ) or S25 (CaMKIIα) residue in mice brain. Mice brain samples shown in S5 Fig were also subjected to SRM analysis with mass spectrometry method for analyzing the phosphorylation of S26 (CaMKIIβ) or S25 (CaMKIIα) residues. Representative chromatograms shown in left indicated that a synthesized and heavy-labeled phosphorylated peptide, of which sequence is identical to a trypsin-digested peptide sequence corresponding to S26 (CaMKIIβ) or S25 (CaMKIIα) was detected at retention time approximately 20 min. Medium-labeled peptide sample (derived from internal control mixture) and light-labeled peptide sample (derived from individual samples) also showed a peak at retention time approximately 20 min. The product ion spectrum on the right shows that each product ion from the 5 different transitions in the 3 samples has a similar intensity distribution. These results suggest that a peptide corresponding to phosphorylated S26 (CaMKIIβ) or S25 (CaMKIIα) was included in trypsin-digested mice brain samples. CaMKIIβ, calmodulin-dependent protein kinase IIβ; SRM, selected reaction monitoring. (PDF) [file pbio.3001813.s013.pdf]

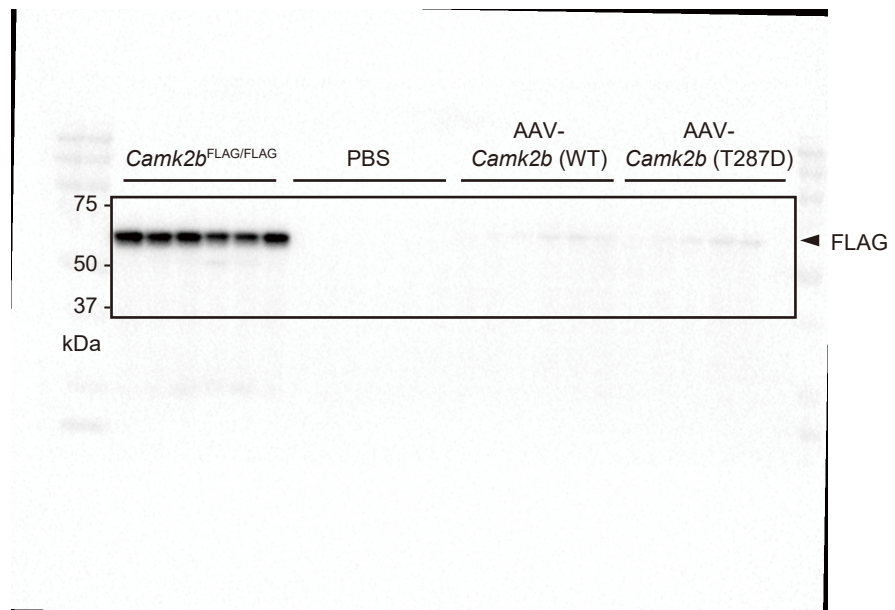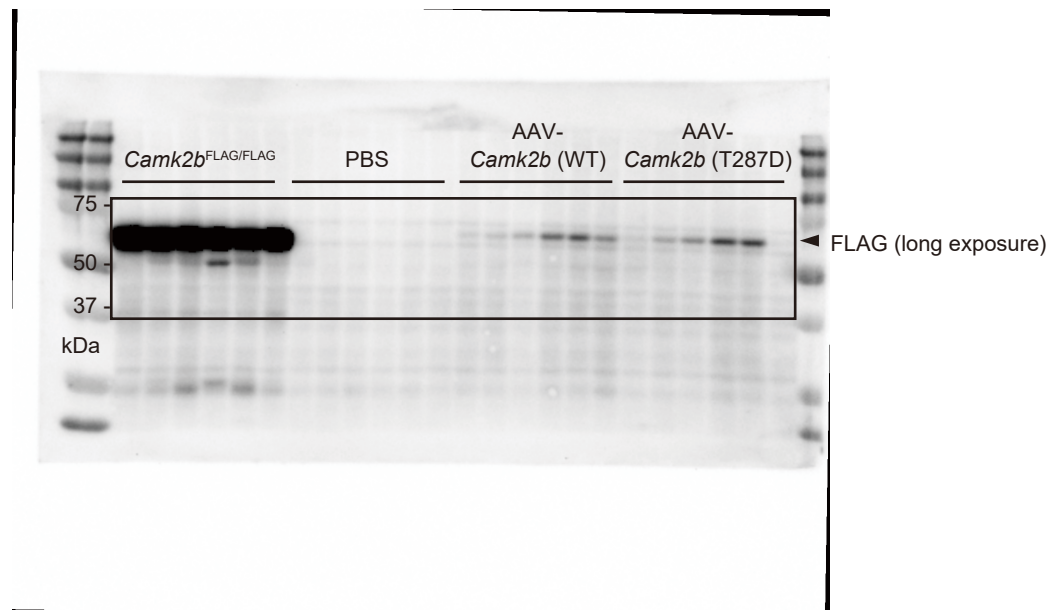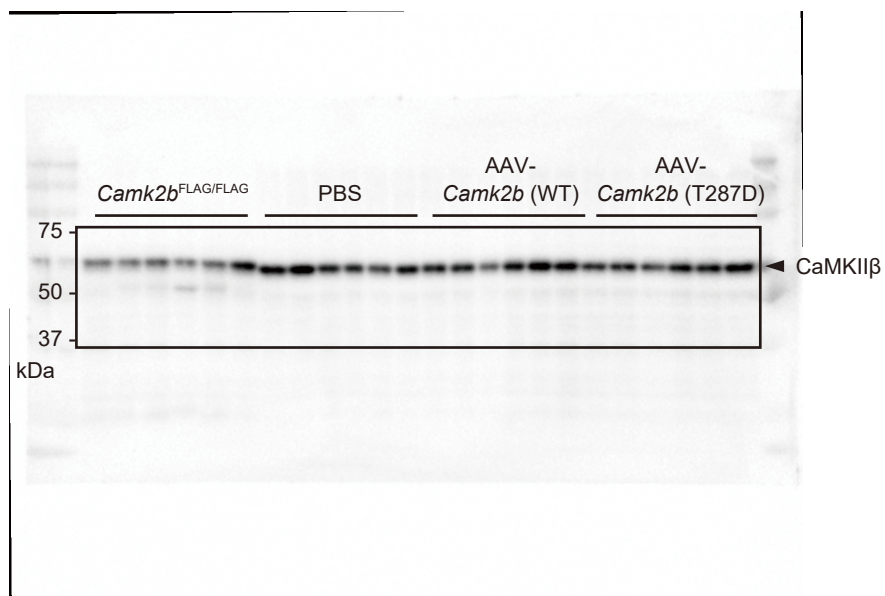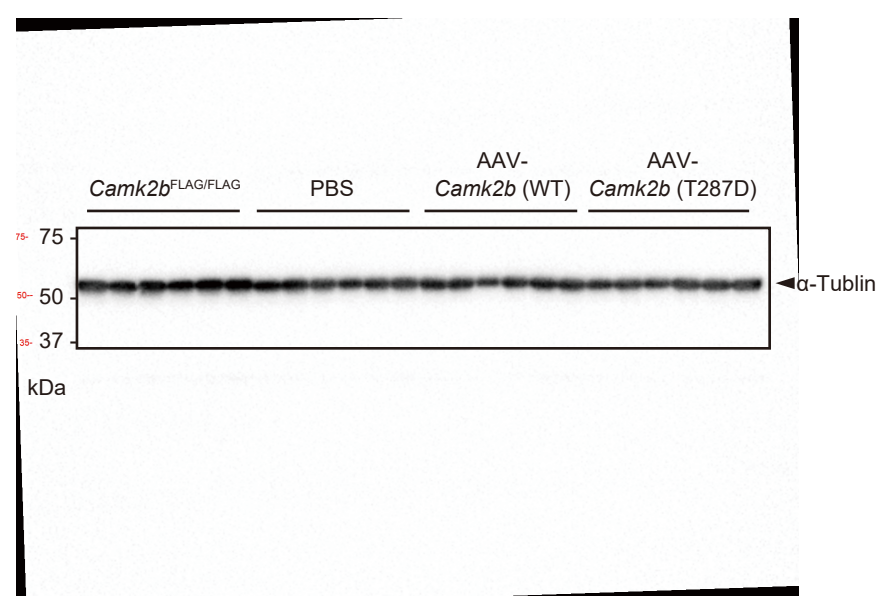

Supplement: S2 Data — (PDF) [file pbio.3001813.s018.pdf]

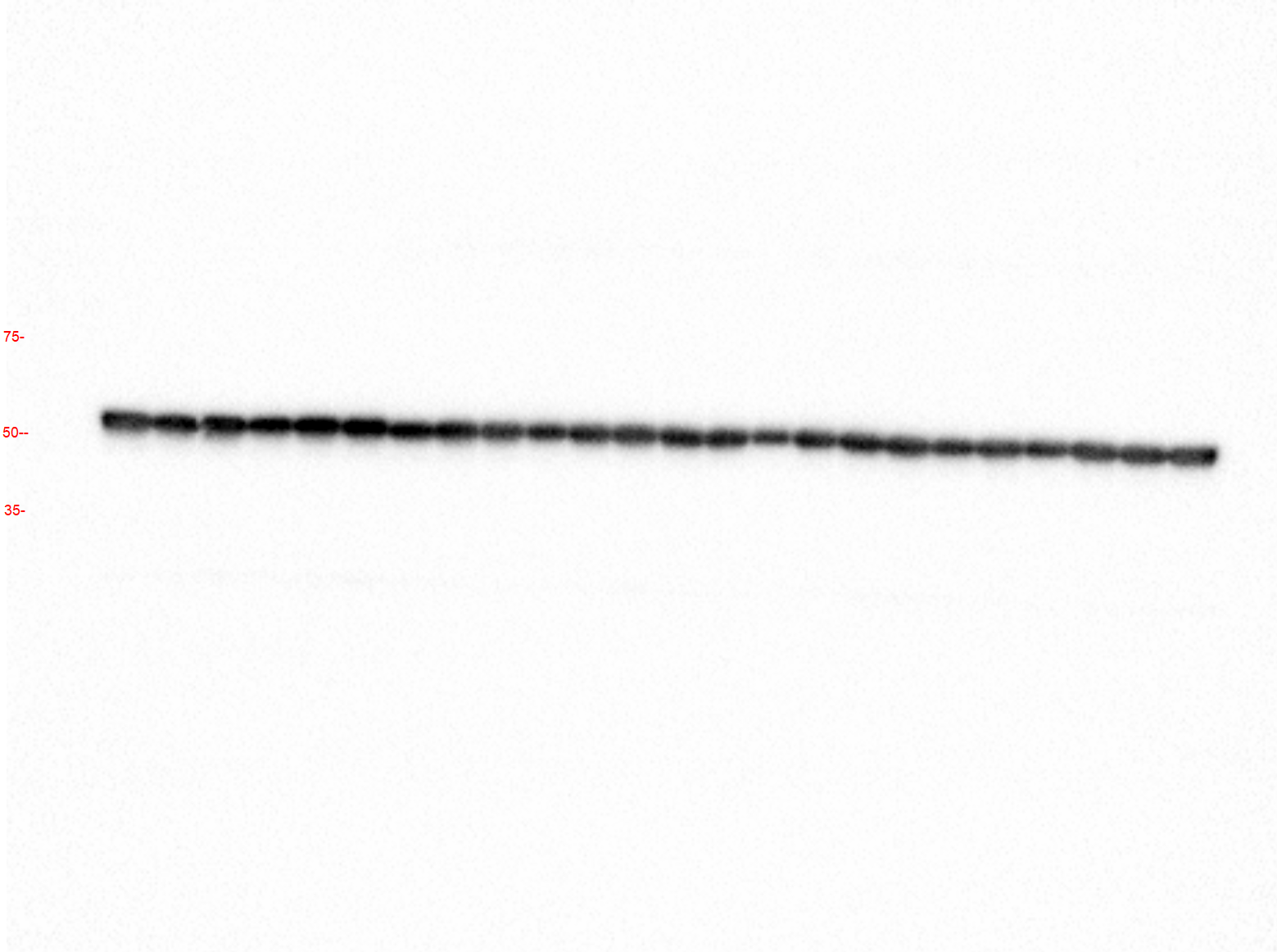

Supplement: S3 Data — (ZIP) [file pbio.3001813.s019.zip › Sourcedata3/Tublin.tif]

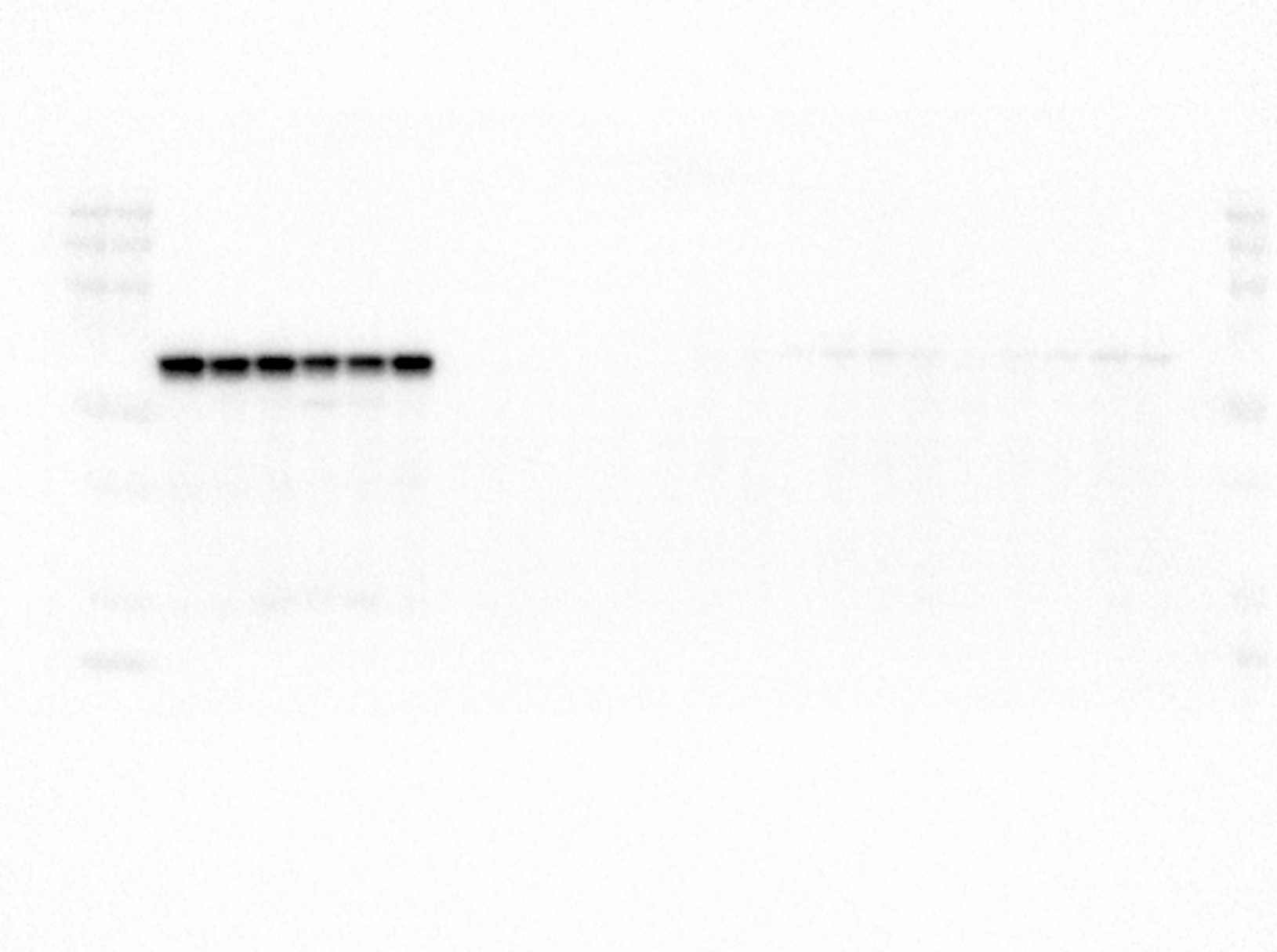

Supplement: S3 Data — (ZIP) [file pbio.3001813.s019.zip › Sourcedata3/FLAG.tif]

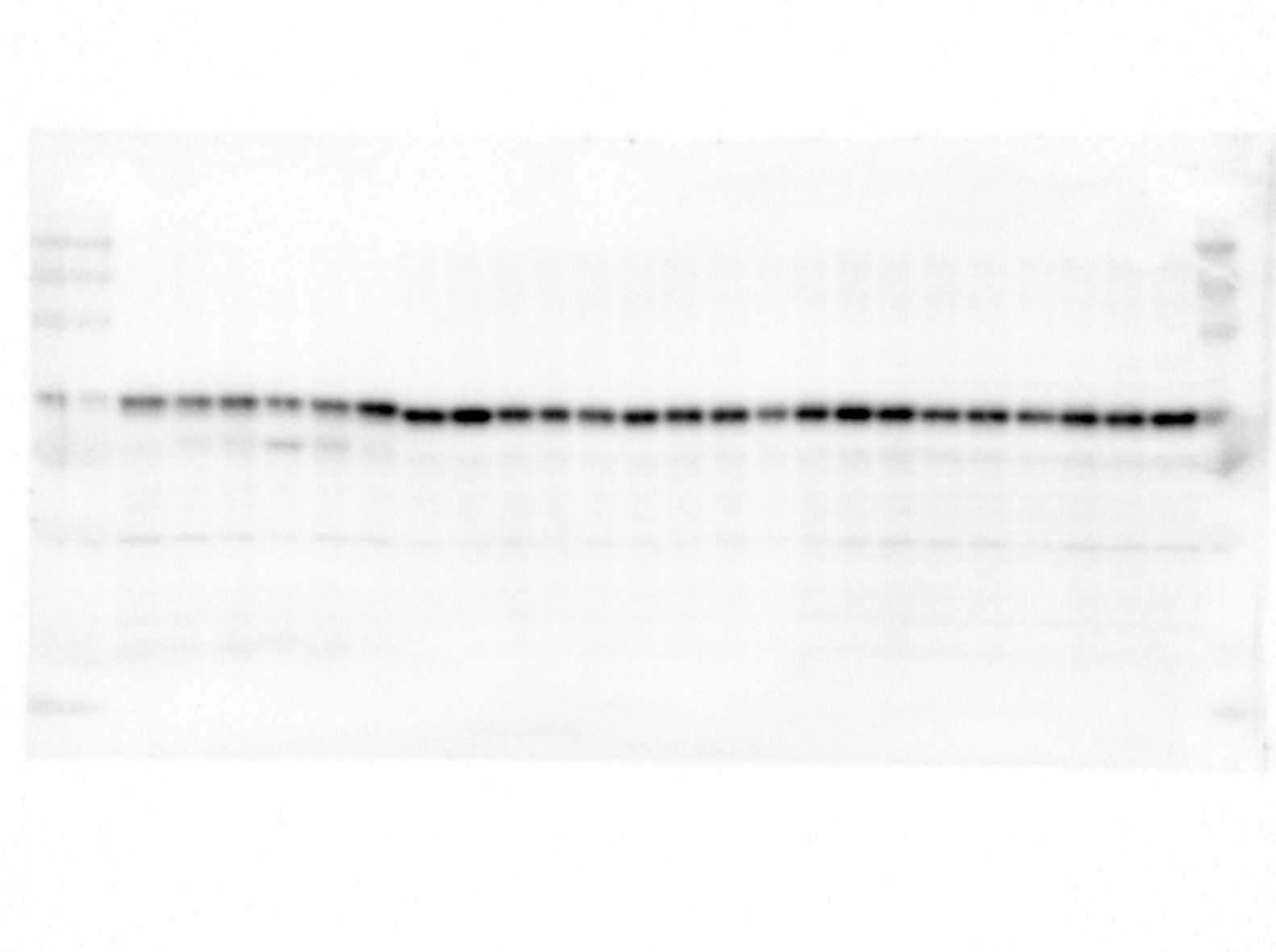

Supplement: S3 Data — (ZIP) [file pbio.3001813.s019.zip › Sourcedata3/Camk2b.tif]

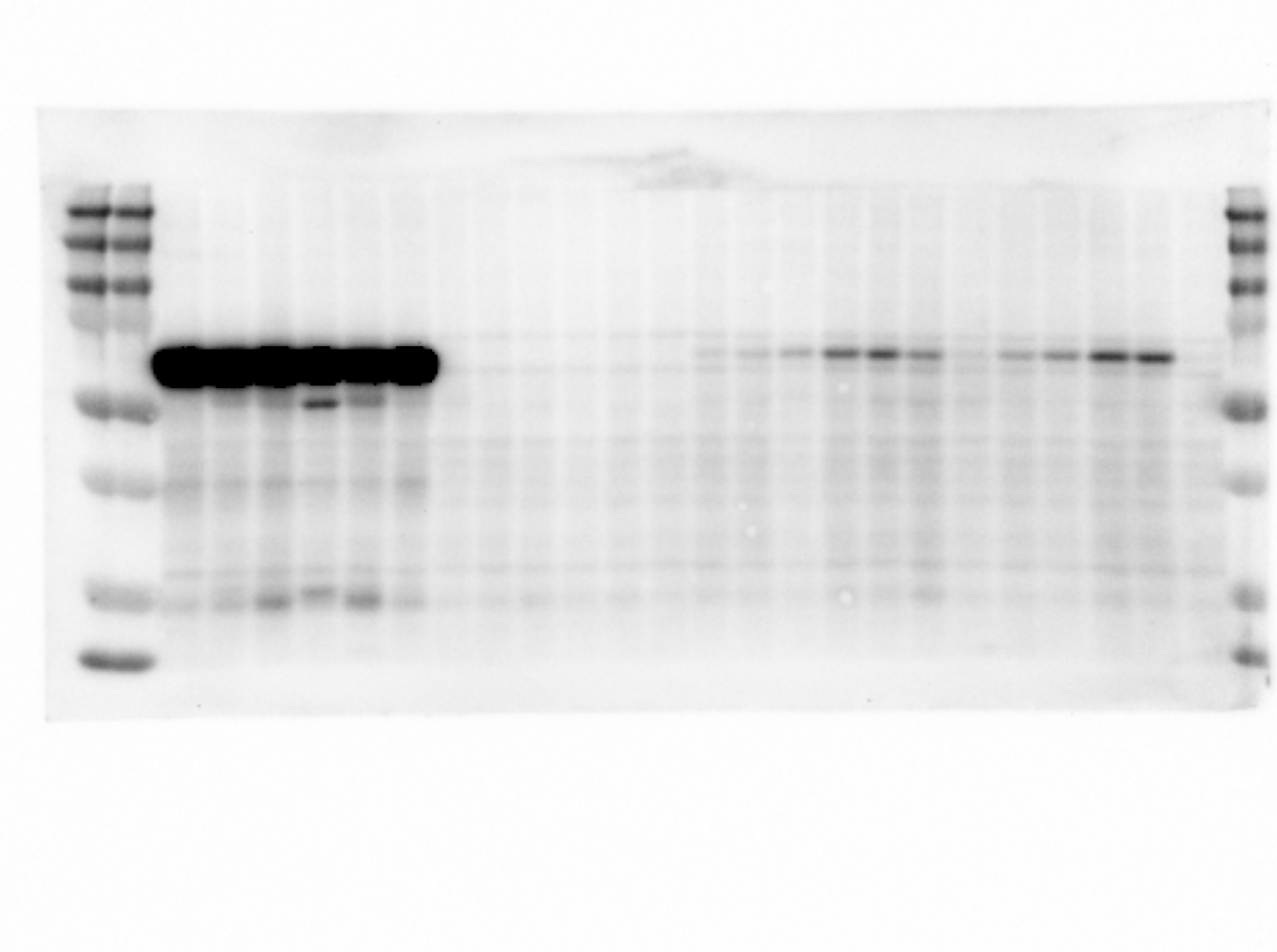

Supplement: S3 Data — (ZIP) [file pbio.3001813.s019.zip › Sourcedata3/FLAG-long-exposure.tif]
