# Supplementary material for: Distinct phosphorylation states of mammalian CaMKIIβ control the induction and maintenance of sleep
Source: PLoS Biol. 2022 Oct 4;20(10):e3001813. doi: 10.1371/journal.pbio.3001813 (PMC9531794; doi:10.1371/journal.pbio.3001813)
Supplement: S8 Fig — (A) The genotyping of Camk2b KO mice. The relative amount of intact DNA for each target sequence was normalized to be 100% for WT mouse. The qPCR was performed with 2 independent primer pairs #1 and #2 for the 3 target sites. When the 0.5% criteria were met in either set, the mouse was considered a KO mouse. The qPCR results indicate that triple gRNA sequences used for this KO effectively induced deletion mutations in the chromosomal target-1 and target-2 regions covered by the alternative primer pair #2. The persistent presence of target-3 signals amplified by both primer pairs #1 and #2 suggests that the chromosomal target-3 region was not effectively deleted by the gRNA used in this experiment. All the mice (n = 5) were confirmed as KO mice by the qPCR with primer pair #2. (B-D) Sleep parameters (B and D) and sleep profiles (C) measured by EEG/EMG recordings for Camk2b KO mice and WT C57BL/6N mice. (E, F) Normalized EEG power spectra (E) and NREM power density in typical frequency domains (F) of Camk2b KO mice and WT C57BL/6N mice. EEG power was normalized relative to the total power in each frequency band. (G, H) EEG power spectra (G) and NREM power density in typical frequency domains (H) of Camk2b KO mice and WT C57BL/6N mice without normalization. The underlying data can be found in S1 Data. Error bars: SEM, *p < 0.05, **p < 0.01, ***p < 0.001, n.s.: no significance. EEG, electroencephalogram; EMG, electromyogram; KO, knockout; NREM, nonrapid eye movement; WT, wild-type. (PDF) [file pbio.3001813.s008.pdf]

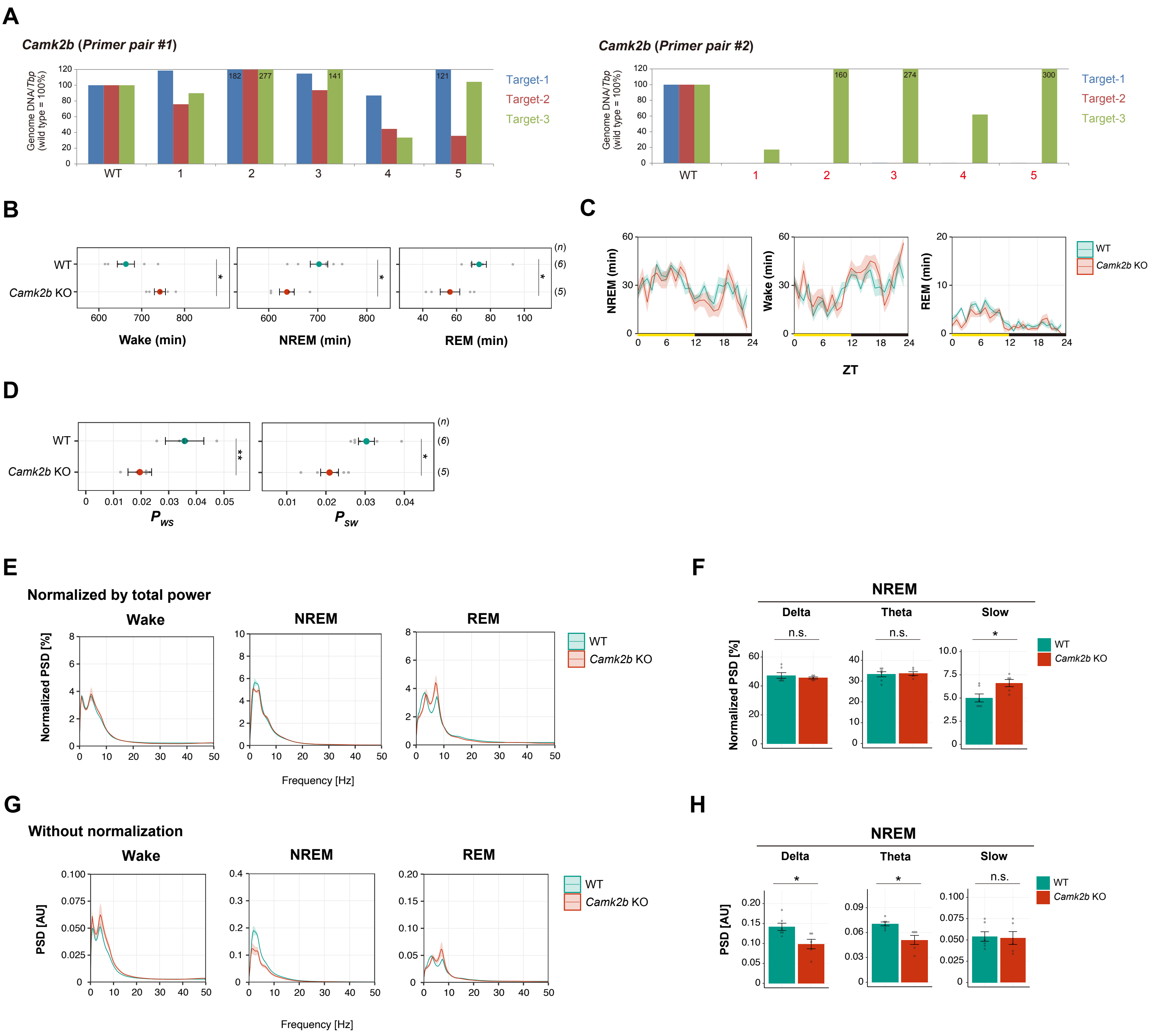

A
Camk2b (Primer pair #1)
Camk2b (Primer pair #2)
Genome DNA/Tbp (wild type = 100%)
Target-1
Target-2
Target-3
WT
B
C
Wake (min)
NREM (min)
REM (min)
Camk2b KO
ZT
D
P_{WS}
P_{SW}
E
Normalized by total power
Wake
NREM
REM
Normalized PSD [%]
Frequency [Hz]
F
Delta
Theta
Slow
n.s.
G
Without normalization
PSD [AU]
H

Figure S8
