# Supplementary material for: Distinct phosphorylation states of mammalian CaMKIIβ control the induction and maintenance of sleep
Source: PLoS Biol. 2022 Oct 4;20(10):e3001813. doi: 10.1371/journal.pbio.3001813 (PMC9531794; doi:10.1371/journal.pbio.3001813)
Supplement: S12 Fig — (A) Sleep/wake parameters of mice expressing the quadruple-phosphomimetic CaMKIIβ mutants related to T311. Multiple comparison tests were performed between all individual groups. Error bars: SEM, *p < 0.05, **p < 0.01, ***p < 0.001. (B) Expression levels of each mutant in the 293T analyzed by the dot blotting of cell lysates. The values are shown in the mean ± SD (n = 2, independent experiments). (C) In vitro kinase activity of CaMKIIβ phosphomimetic mutants. Phosphorylation (%) indicates the percentage of the phosphorylated substrate relative to the total peptide in the presence or absence of CaM. The represented values are the mean ± SD (n = 2, independent experiments). The relative amount of CaMKIIβ in the cell lysates was adjusted to be the same level as WT. The mutants with blue labels exhibited <25% lower expression than the WT in both trials (see black and orange dashed lines in panel B). The mutants with green labels exhibited <25% lower expression than the WT in one of the 2 trials. When the expression level is <25% compared to WT, the relative amount of the CaMKIIβ mutant in the kinase assay was lower than that of the WT. (D) Time series changes of sleep-controlling residues phosphorylation under different Ca2+ conditions in vitro. The represented values are the mean ± SD (n = 2 independent experiments). The signal intensity of the detected peptides was normalized to the maximum value in the time series. The quantified values at 0 min were obtained from the sample before adding CaM and were shared in every Ca2+ conditions. The dashed lines trace the dynamics of T287 phosphorylation in the 0.5 mM Ca2+ condition. (E) Example chromatogram of SRM measurement for pS182 peptide. The red line indicates the retention time of a peptide phosphorylated at S182. The blue line indicates the retention time of a peptide phosphorylated at T177. (F) S26-, T306-, T307-, or T311-phosphorylated peptides from 293T cell lysates. The 293T cells were transfected with plasmids ove [file pbio.3001813.s012.pdf]

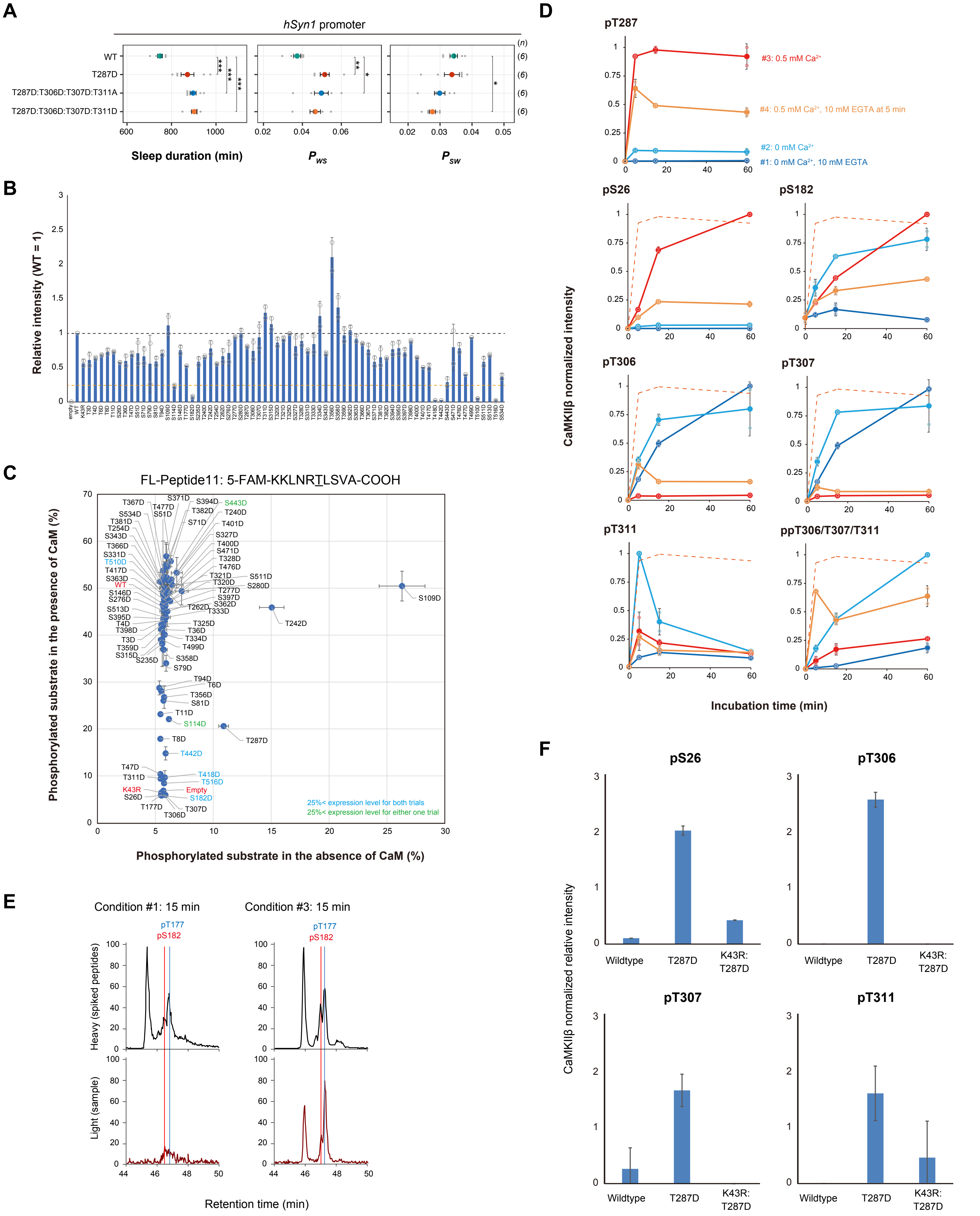

A
hSyn1 promoter
WT
T287D
T287D:T306D:T307D:T311A
T287D:T306D:T307D:T311D
Sleep duration (min)
Pws
Psw
B
Relative intensity (WT = 1)
C
FL-Peptide11: 5-FAM-KKLNRTLSVA-COOH
Phosphorylated substrate in the presence of CaM (%)
Phosphorylated substrate in the absence of CaM (%)
25%< expression level for both trials
25%< expression level for either one trial
D
pT287
#3: 0.5 mM Ca2+
#4: 0.5 mM Ca2+, 10 mM EGTA at 5 min
#2: 0 mM Ca2+
#1: 0 mM Ca2+, 10 mM EGTA
pS26
pS182
pT306
pT307
pT311
ppT306/T307/T311
CaMKIIβ normalized intensity
Incubation time (min)
E
Condition #1: 15 min
Condition #3: 15 min
pT177
pS182
Heavy (spiked peptides)
Light (sample)
Retention time (min)
F
pS26
pT306
pT307
pT311
Wildtype
T287D
K43R: T287D
CaMKIIβ normalized relative intensity


Figure S12
